# C_sp_, C_sp_^2^, and C_sp_^3^ Hydrocarbyl Group Migration from Pd(II) to P(III): Accessing Metallophosphoranes via Nonspectator Ligand Reactivity

**DOI:** 10.1002/anie.202519496

**Published:** 2025-11-13

**Authors:** Lily Ueh‐Hsi Wang, Akira Tanushi, Peter Müller, Alexander T. Radosevich

**Affiliations:** ^1^ Department of Chemistry Massachusetts Institute of Technology 77 Massachusetts Avenue Cambridge Massachusetts 02139 USA

**Keywords:** Chelate, Cooperativity, Ligand migration, Phosphorane, Phosphorus ligand

## Abstract

Migration of palladium‐bound hydrocarbyl ligands to tricoordinate phosphorus ligand P(N(o‐N(2‐pyridyl)C_6_H_4_)_2_) (**L**) is demonstrated across a series of Pd(II) organometallic complexes bearing C_sp_, C_sp_^2^, and C_sp_^3^ groups. Treatment of ligand **L** with *cis*‐[(TMEDA)PdI(C_6_H_5_)], *cis*‐[(TMEDA)PdBr(CH_2_C_6_H_5_)], [(η^3^‐C_3_H_5_)PdCl]_2_, and *trans*‐[PdBr(C≡C─C_6_H_5_)(PPh_3_)_2_], respectively, results in migration of the hydrocarbyl group from Pd to P, yielding isolable (σ^4^‐P)─Pd palladaphosphoranes: **L^Allyl^•Pd^Cl^**, **L^Bn^•Pd^Br^**, **L^Ph^•Pd^I^**, and **L^CCPh^•Pd^Br^**. The mechanistic pathway of the palladaphosphorane formation was investigated by in situ NMR experiments and DFT calculations, suggesting an α‐migration mechanism. Halide exchange with NaBr or NaI affords the corresponding bromide and iodide congeners without disrupting the palladaphosphorane connectivity. ^31^P NMR chemical shifts correlate systematically with the identity and hybridization of the hydrocarbyl group, and electronic structure analyses attribute observed trends to variations in the s/p hybrid compositions of the local P─C bond orbitals. This work establishes an underappreciated facet in the reactivity landscape of Pd complexes bearing tricoordinate phosphorus (σ^3^─P) ligands by demonstrating their ability to undergo nonspectator metal‐to‐ligand group transfer, with implications for designing bifunctional ligand architectures capable of cooperative catalysis.

## Graphical abstract

Well‐defined migration of C_sp_, C_sp_^2^, and C_sp_^3^ hydrocarbyls from Pd(II) compounds to a nontrigonal P(III) ligand scaffold is reported. The structural and spectroscopic features of the resulting complexes reveal a conserved Pd─P interaction. The results demonstrate the nonspectator role of structurally constrained tricoodinate P(III) ligands in palladium chemistry.

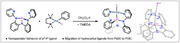

Phosphorus(III) ligands are foundational in coordination chemistry and homogeneous catalysis, modulating the steric and electronic properties of metal complexes.^[^ ^1^, ^2^, ^3^ ^]^ Although conventionally cast as spectators with limited direct involvement in bond‐forming or ‐breaking events (Figure 1a),^[^ ^4^ ^]^ a substantial—albeit underappreciated—body of literature implies a more active role of σ^3^─P ligands in late transition metal‐mediated processes. Among these reports, the scrambling and exchange of aryl substituents between σ^3^─P ligands and late metals, often in conjunction with catalyst deactivation,^[^ ^5^, ^6^ ^]^ are well‐documented.^[^ ^7^, ^8^, ^9^, ^10^ ^]^ Although frequently attributed to lower‐coordinate metal‐phosphide (σ^2^─P)─M intermediates^[^ ^11^, ^12^, ^13^, ^14^, ^15^, ^16^ ^]^ as by Kong and Cheng^[^ ^17^ ^]^ (Figure 1b), the involvement of higher‐coordinate phosphorus intermediates—metallophosphoranes (σ^4^─P)–M^[^ ^18^, ^19^, ^20^, ^21^, ^22^, ^23^, ^24^, ^25^, ^26^, ^27^, ^28^, ^29^, ^30^, ^31^, ^32^, ^33^, ^34^, ^35^, ^36^ ^]^— in such processes has also been suggested. For instance, Grushin reasoned that aryl exchange in (Ph_3_P)_2_Pd(Ar)(F) evolves through P‐aryl palladaphosphoranes (Figure 1b),^[^ ^37^, ^38^ ^]^ a conjecture that is buoyed by DFT results from MacGregor.^[^ ^39^, ^40^ ^]^ Indeed, Riess and Miyamoto have independently isolated late metal metallophosphoranes (Fe/Ru and Ir, respectively) that undergo a 1,2‑aryl shift from phosphorus to metal, providing experimental support for P‑aryl→M migration via a σ^4^‑P manifold. However, direct evidence and isolation of palladaphosphoranes from aryl migration reactions have not been reported, presumably because a systematic approach to their preparation, design, and study has been lacking.^[^ ^18^, ^19^, ^20^, ^21^, ^22^, ^23^, ^24^, ^25^, ^26^, ^27^, ^28^, ^29^, ^30^, ^31^, ^32^, ^33^, ^34^, ^35^, ^36^ ^]^

**Figure 1:**
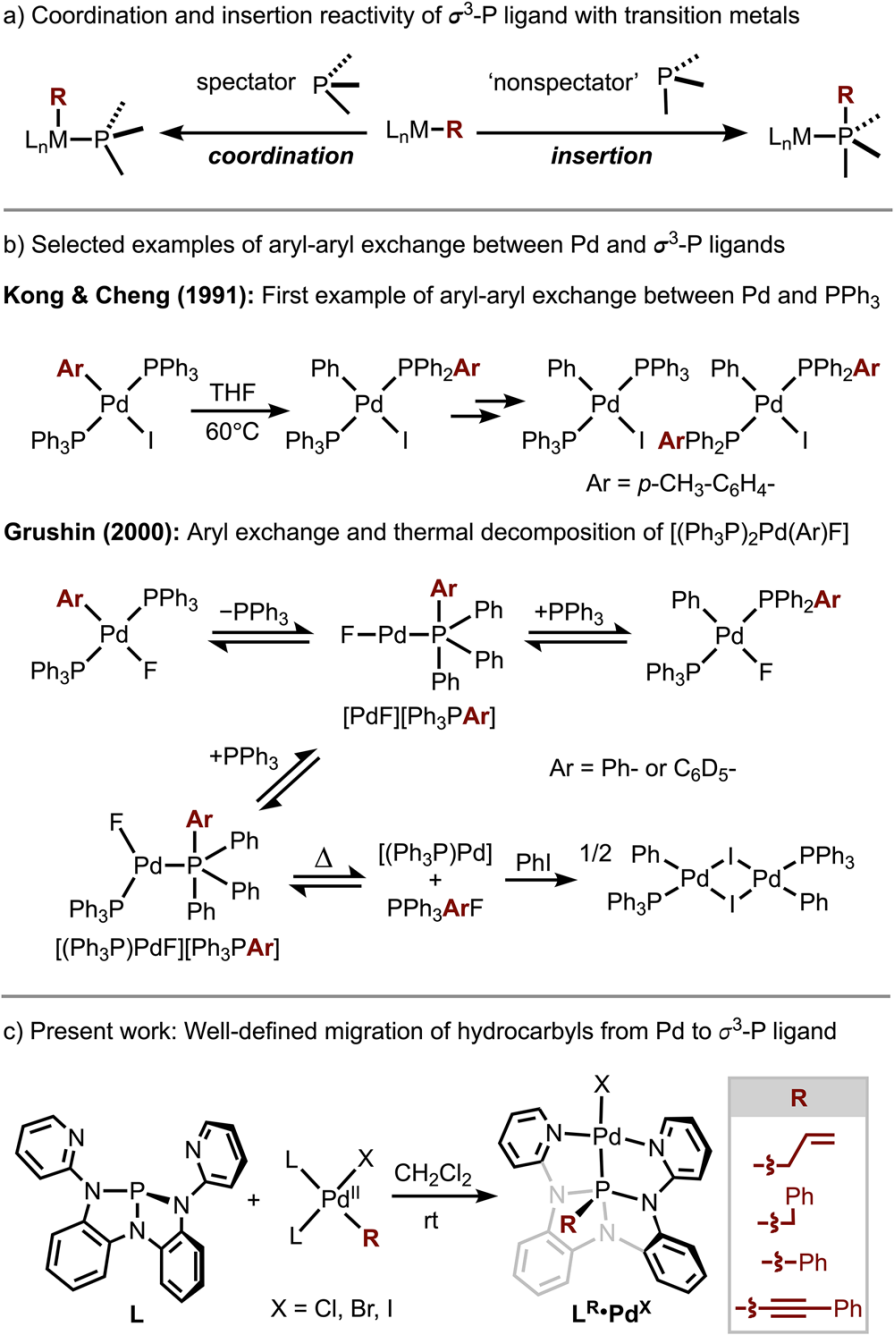
Migration of hydrocarbyl ligands from Pd(II) to nontrigonal σ^3^‐P ligand **L**.

We recently showed that geometrically deformed, electrophilic σ^3^‑P triamidophosphorus compounds^[^ ^41^ ^]^ can behave as nonspectator ligands^[^ ^42^, ^43^ ^]^ that interconvert between σ^3^‑P–M and σ^4^‑P–M states via L/X‑type switching^[^ ^44^ ^]^ and even insertion into late metal M─Me^[^ ^45^ ^]^ or M─H^[^ ^46^ ^]^ bonds. Guided by this biphilic behavior, we hypothesized that an appropriately designed non‐trigonal σ^3^‐P ligand could sequester phenyl groups from Pd(II) during metalation to give isolable P‐phenyl (σ^4^‐P)–Pd palladaphosphoranes. Here we demonstrate that the electrophilic triamide P(N(o‐N(2‐pyridyl)C_6_H_4_)_2_ (**L**) indeed accepts transfer of hydrocarbyl fragments spanning C_sp_, C_sp_^2^, and C_sp_^3^ hybridization from Pd(II), furnishing a family of structurally authenticated P‐alkynyl, P‐aryl, P‐allyl/benzyl palladaphosphoranes (Figure 1c). These results deliver the first direct structural evidence for P‐aryl metallophosphoranes at palladium and reveal a general metal‐to‐ligand group‐transfer manifold.

To evaluate the feasibility of C(sp^2^) hydrocarbyl group transfer, ligand **L** (P(N(o‐N(2‐pyridyl)C_6_H_4_)_2_)) was reacted with *cis*‐[(TMEDA)PdI(C_6_H_5_)]^[^ ^47^ ^]^ in CH_2_Cl_2_ at ambient temperature for 2 h (Figure 2a). ^1^H NMR spectra indicated formation of a new 1:1 complex between the chelating ligand **L** and Pd, with loss of TMEDA but retention of the phenyl fragment. Interestingly, the complex thus formed exhibits an apparent equivalence of the pyridyl moieties, implying a time‐averaged molecular symmetry of *C_s_* or higher. Although this observation itself does not exclude a κ^2^‐binding of ligand **L** to Pd with fast intramolecular pyridyl exchange, further data evince a static κ^3^‐chelation of **L** to Pd attended by a substantial change in local chemical environment around P. Namely, ^31^P NMR spectra show that ligand **L** (δ 140.0 ppm) is transformed upon metalation into a single new species whose resonance is shifted significantly upfield (δ –29.1 ppm). Markedly, the aryl region of ^13^C{^1^H} NMR spectrum displayed a doublet resonance at δ 138.0 ppm with a large coupling constant (^1^ *J*_PC_ = 108.9 Hz) indicative of a direct P─C bond. Taken together, the totality of the multinuclear NMR spectroscopies is consistent with intramolecular migration of the phenyl group from Pd to P upon metalation of **L**, in which the P center is transformed into a higher‐coordinate P‐phenyl palladaphosphorane formulated as **L^Ph^•Pd^I^** (Figure 2, top right).

**Figure 2:**
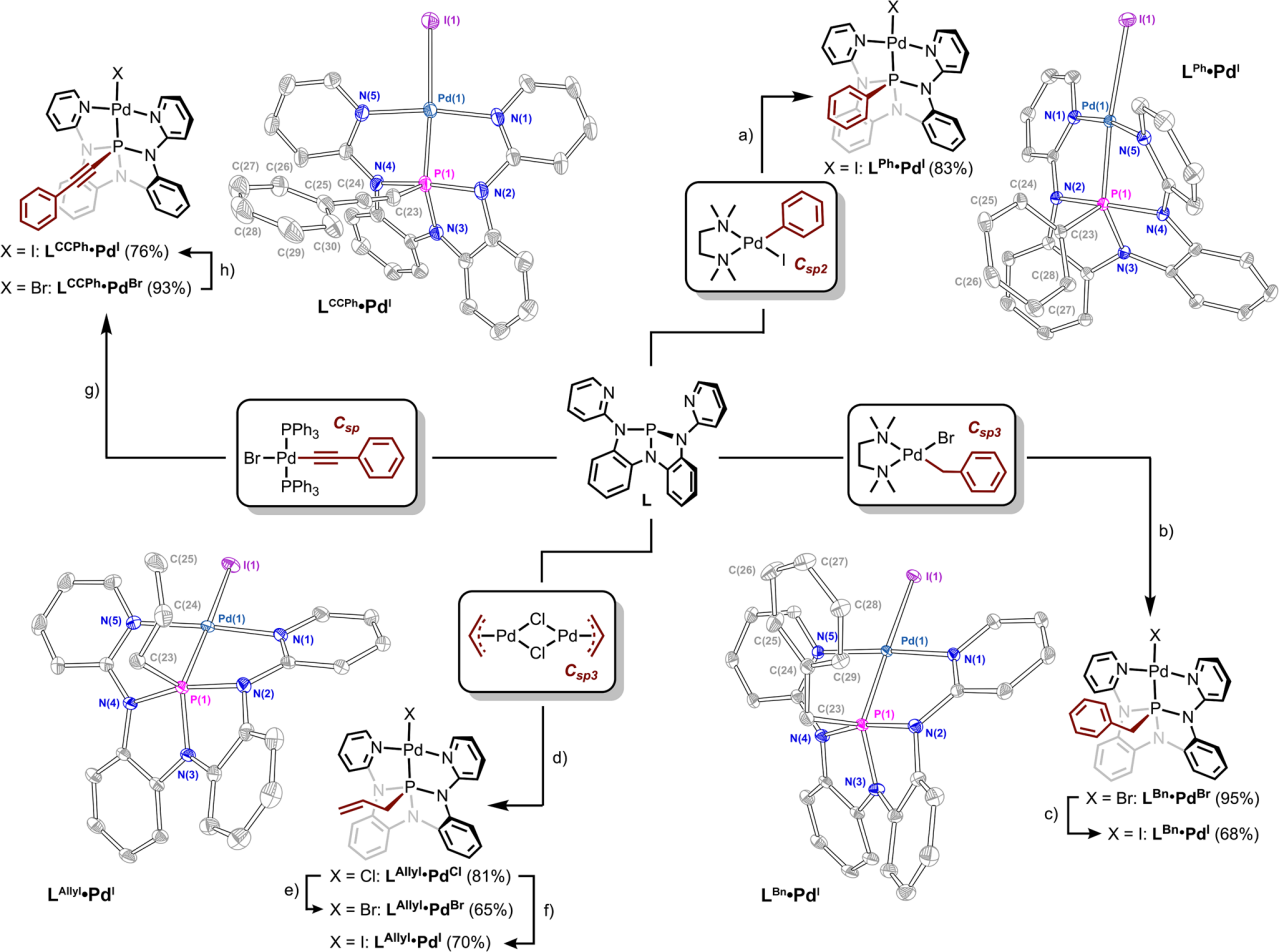
Synthesis and selected solid‐state structures of palladaphosphoranes **L^R^•Pd^X^** (R = Allyl, Bn, Ph, CCPh; X═Cl, Br, I). a) 1 equiv. cis‐[(TMEDA)PdI(C_6_H_5_)], CH_2_Cl_2_, rt 2 h; b) 1 equiv. cis‐[(TMEDA)PdBr(CH_2_C_6_H_5_)], CH_2_Cl_2_, rt 2 h; c) 10 equiv. NaI, THF:MeCN = 1:1, rt o/n; d) 0.5 equiv. [PdCl(C_3_H_5_)]_2_, THF, rt o/n; e) 10 equiv. NaBr, THF:MeCN = 1:1, rt 3d; f) 10 equiv. NaI, THF:MeCN = 1:1, rt 4 h; g)1 equiv. trans‐[PdBr(C≡C–C_6_H_5_)(PPh_3_)_2_], CH_2_Cl_2_, rt 2 h; h) 10 equiv. NaI, THF:MeCN = 1:1, rt o/n. Thermal ellipsoids are rendered at 50% probability level. All hydrogen atoms and solvent molecules are omitted for clarity. For crystallographic details, see Supporting Information section IV.

Despite the transient nature of previously invoked arylpalladaphosphoranes, the phenyl migration product **L^Ph^•Pd^I^** is indefinitely stable under inert conditions, enabling isolation of diffraction‐quality dark orange single crystals by vapor diffusion of pentane into a THF solution. The solid‐state structure confirmed migration of the phenyl fragment to phosphorus (P(1)‐C(23) = 1.8235(16)Å), along with *k* ^3^‐binding of the N,P,N‐chelating ligand to the square planar Pd(II). The Pd─P bond length is 2.2301(5) Å. The local geometry about P is best described as a distorted square pyramid (*τ*_5_ = 0.38), with a broad basal plane spanned by the pyridyl‐bearing N‐substituents (N(2)−P(1)−N(4) = 165.94(7)°) and metal‐containing posterior angle N(3)−P(1)−Pd(1) = 143.30(5)°. The angles including the C(phenyl) apex are comparatively acute (Pd(1)−P(1)−C(23) = 110.12(6)°, C(23)−P(1)−N(3) = 106.50(7)°).

The hydrocarbyl migration observed with phenyl substitution is not unique to C(sp^2^) fragments but also applies to C(sp^3^) moieties. For instance, metalation of ligand **L** with *cis*‐[(TMEDA)PdBr(CH_2_C_6_H_5_)]^[^ ^48^ ^]^ (1 equiv.) in CH_2_Cl_2_ at ambient temperature for 2 h resulted in clean formation of a new species with an upfield singlet resonance (^31^P δ –14.4 ppm), as anticipated for a higher‐coordinate P nucleus in benzylpalladaphosphorane **L^Bn^•Pd^Br^** (Figure 2b). Correspondingly, the P‐bound benzyl fragment was evident the ^1^H NMR spectra (doublet, δ 3.45 ppm, ^2^ *J*_PH _= 11.2 Hz) and ^13^C{^1^H} NMR spectra (doublet, δ 44.0 ppm, ^1^ *J*_PC _= 69.8 Hz).

Related C_sp_^3^ transfer reactivity was observed with π‐allyl palladium precursors. Metalation of the ligand **L** with 0.5 equiv. of allylpalladium(II) chloride dimer [(η^3^‐C_3_H_5_)PdCl]_2_ in THF at ambient temperature overnight yielded an orange‐yellow powder (Figure 2d) (δ –12.3 ppm), whose chemical shift implies the formation of a pentacoordinate phosphorus center. In the ^1^H NMR spectrum, the characteristic resonances of a linear allyl fragment could be observed, and integration indicated the presence of a single allyl group per ligand complex. The ^13^C{^1^H} NMR spectrum displayed a signal at δ 42.9 ppm, which is split into a doublet by coupling of the allylic C to P (^1^ *J*_PC_ = 74.0 Hz), indicative of a direct P─C bond. Together, these spectroscopic data are consistent with formation of a P‐allyl palladaphosphorane **L^Allyl^•Pd^Cl^**, as depicted in Figure 2. Single‐crystal X‐ray diffraction further corroborated the solution‐state assignment (Figure S36).

Notably, the observed hydrocarbyl migration extends even to migration of C_sp_‐hybridized fragment transfer. Reaction of *trans*‐[PdBr(C≡C─C_6_H_5_)(PPh_3_)_2_]^[^ ^49^ ^]^ with **L** afforded the phenylacetylenyl‐substituted derivative **L^CCPh^•Pd^Br^** (Figure 2g). In contrast to the allyl‐, benzyl‐, and phenyl analogues, **L^CCPh^•Pd^Br^** exhibited a significant upfield ^31^P NMR shift (singlet, δ–55.7 ppm). The ^13^C{^1^H} NMR spectrum displayed diagnostic doublets at δ 85.1 ppm (^1^ *J*_PC_ = 178.6 Hz) and δ 96.7 ppm (^2^ *J*_PC_ = 34.9 Hz), indicative of P─C≡C─Ph bond formation. Structure determination by X‐ray diffraction confirmed the identity of the P‐alkynyl product (Figure S42); a C≡C bond length of 1.205(3) Å is consistent with C≡C triple‐bond character, and IR spectra further corroborated this assignment with a C≡C stretch at *ν* = 2166 cm^−1^.

In effect, across the reactions of Pd(II)‐alkyl, ‐aryl, and ‐alkynyl compounds with ligand **L**, the clean appearance of single ^31^P NMR resonances and the absence of detectable byproducts in each case suggest that the constrained geometry and enhanced electrophilicity of the ligand scaffold promote the formation and stabilization of the resultant (σ⁴‐P) ─Pd complexes by hydrocarbyl migration from Pd(II) to σ^3^‐P ligand, regardless of C‐hybridization.

To gain insight into the mechanism of metallophosphorane formation, the reaction of **L** and (TMEDA)PdPhI was monitored by ^31^P NMR spectroscopy at 253 K. Notably, only free **L** (δ 140.0 ppm) and product **L^Ph^•Pd^I^** (δ –29.1 ppm) were observed during the reaction time course; no intermediates were detected (see SI, Figure S44). Evidently, metallophosphorane formation is at least as fast as association of **L** to Pd(II). In view of prior work on related systems,^[^ ^45^ ^]^ an initial *k* ^2^‐P,N‐chelated association complex **L•Pd^PhI^** (Figure 3) would be expected, and further evolution to the metallophosphorane **L^Ph^•Pd^I^** would then involve a sequence of phenyl migration and pendant pyridine binding. Figure 3a depicts a two‐dimensional relaxed surface scan (implemented within Orca 6.1.0,^[^ ^50^ ^]^ grid density of 100 points, r^2^SCAN‐3c level^[^ ^51^ ^]^) along the relevant *d*(P‐C_phenyl_) and *d*(Pd‐N_pyridine_) coordinates. The potential surface reflects a pronounced asynchrony in bond formation, for which phenyl α‐migration significantly precedes pyridine binding. Indeed, unconstrained geometry optimizations map a stepwise minimum energy pathway (Figure 3b) involving initial α‐migration of the phenyl group from Pd to P (**TS‐I**), subsequently proceeding to the final metallophosphorane via an energetic plateau involving shallow intermediate (**INT**, Δ*G* = 13.8 kcal mol^−1^) and low‐barrier pyridine binding (**TS‐II**, Δ*G* = 14.7 kcal mol^−1^). The relatively low energy of **TS‐I** (Δ*G* = 19.7 kcal mol^−1^) and overall reaction driving force (Δ*G* = –26.7 kcal mol^−1^) and are qualitatively consistent with the fast and irreversible formation of **L^Ph^•Pd^I^**. An intrinsic bond orbital (IBO) analysis^[^ ^52^, ^53^ ^]^ of the intrinsic reaction coordinate proceeding through **TS‐I** (Figure 3c) illustrates that the facility of α‐migration is connected to the acceptor character of the nontrigonal phosphorus center. The initial σ(Pd‐C_phenyl_) IBO can be seen to transform into the newly formed σ(P─C_phenyl_) bond orbital, consistent with transfer of the σ‐bonding electron pair to an acceptor phosphorus valence. Simultaneously, the σ(Pd─P) IBO can be seen to take on greater Pd character upon phenyl migration, consistent with an increasing covalency of the metal‐phosphorus interaction in metallophosphoranes.^[^ ^41^ ^]^

**Figure 3:**
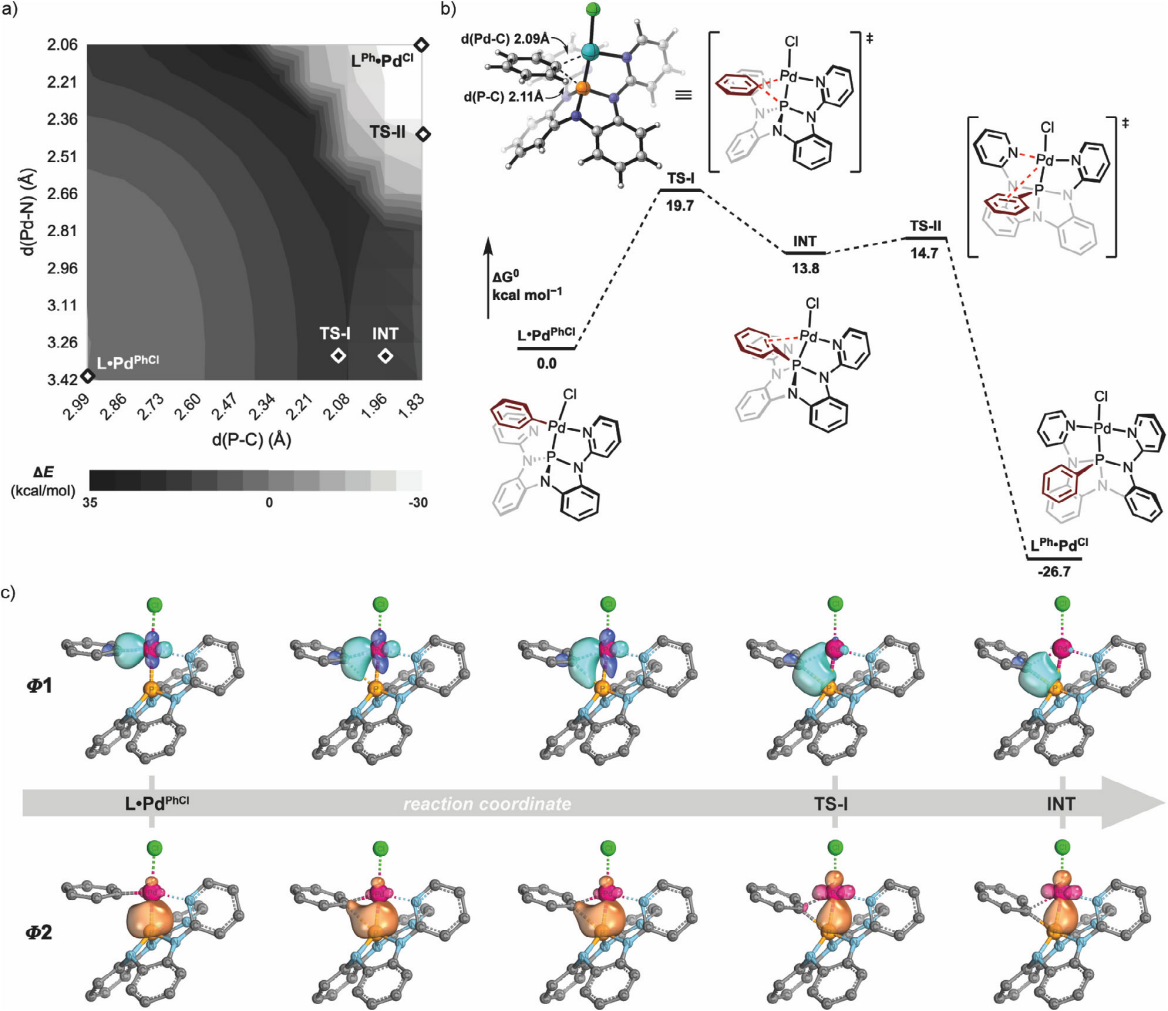
DFT studies of metallophosphorane formation at r^2^SCAN‐3c level of theory. a) Two‐dimensional potential surface from relaxed geometry scan (*N* = 100) along coordinates corresponding to phenyl migration (*d*(P─C), *x*‐axis) and pyridine binding (*d*(Pd‐N), *y*‐axis). Marks indicating the geometries of stationary points from unconstrained geometry optimizations are superimposed on the surface. b) Potential landscape depicting unconstrained stationary points along the minimum energy pathway. c) Progression of relevant IBOs proceeding through TS‐I along the reaction coordinate.

To facilitate structural and spectroscopic comparison among the novel palladaphosphoranes bearing C_sp_, C_sp_^2^, and C_sp_^3^ substituents at P, a series of homologous compounds with a conserved halide ligand was sought. Notably, the higher‐coordinate P‐centers in the aforementioned complexes are sufficiently robust to permit metal‐centered halide substitution reactions. Representatively, treatment of **L^Allyl^•Pd^Cl^** with either NaBr (10 equiv.) or NaI (10 equiv.) in a mixed THF:MeCN (1:1 vol/vol) solvent system resulted in exchange of the Pd‐bound chloride for the exogenous halide, forming the corresponding bromide (**L^Allyl^•Pd^Br^**, ^31^P δ –15.6 ppm) and iodide (**L^Allyl^•Pd^I^**, ^31^P δ –23.4 ppm) analogues (Figure 2e,f). X‐ray diffraction analysis of the solid‐state structures showed that **L^Allyl^•Pd^Br^** (Figure S37) and **L^Allyl^•Pd^I^** (Figure 2 bottom left, Figure S38) are materially similar to **L^Allyl^•Pd^Cl^** in terms of overall structure, local bond lengths, and bond angles around P (cf. Table S9). In a similar fashion, the iodide congeners **L^Bn^•Pd^I^** (^31^P δ –22.1 ppm) and **L^CCPh^•Pd^I^** (δ –63.8 ppm) were synthesized by halide metathesis of the corresponding bromide precursors **L^Bn^•Pd^Br^** and **L^CCPh^•Pd^Br^** (Figure 2c,h).

A structural comparison among the iodide complexes **1^Ph^•Pd^I^, 1^Bn^•Pd^I^, 1^Allyl^•Pd^I^** and **1^CCPh^•Pd^I^** shows that the Pd─P bond length in these complexes does not vary significant (std. dev. = 0.002 Å, Table 1), evidence of the strong buttressing effect of the κ^3^ chelate. Despite the very narrow structural range of Pd‐P bond lengths, the isotropic ^31^P NMR chemical shifts for this series of compounds are found to span more than 40 ppm (Figure 4a, cf. **1^Bn^•Pd^I^**: ^31^P δ –22.1 ppm and **1^CCPh^•Pd^I^**: δ –63.8 ppm), whose trend was found to correlate with the P─C bond lengths (Figure 4b). To trace the origin of the electronic effect giving rise to the observed differential ^31^P NMR chemical shifts, natural localized molecular orbital (NLMO) analysis^[^ ^54^ ^]^ was performed at PBE0/def2‐TZVP^[^ ^55^, ^56^ ^]^ theory level. While the compositions of Pd and P atoms in NLMO(σ_Pd‐P_) (Figure 4d) remain consistent throughout the series, compositions of NLMO(σ_P─C_) (Figure 4e) vary according to the *s*‐character of C fragment (Table 1). Despite the changes in P─C bonds, the phosphorus contribution to NLMO(P‐C) remains essentially constant, indicating that the observed structural differences arise primarily from the electronic character of the hydrocarbyl carbon. In a similar fashion, NLMO analysis also reveals a strong correlation between the *s*‐character of the C atoms in P─C bonds and the experimental δ(^31^P) (Figure 4c). The implication of these results is that electronic effect of the carbon substituent influences the solution‐state NMR response, but does not materially affect the interaction of the higher‐coordinate P fragment with respect to the Pd.

**Table 1:** Selected crystallographic, spectroscopic, and computational data for compounds **L^R^•Pd^I^** (R = Allyl, Bn, Ph, CCPh).

				NLMO(Pd─P)^c)^						NLMO(P─C)^c)^					
						Pd		P				P		C	
Metric	d(Pd─P)^a)^ (Å)	d(P─C)^a)^ (Å)	^31^P δ^b)^ (ppm)	%Pd	%P	%s	%d	%s	%p	%P	%C	%s	%p	%s	%p
**L^Ph^•Pd^I^**	2.2301(5)	1.8235(16)	−29.18	45.850	51.708	12.72	87.2	42.8	56.58	36.860	61.194	34.36	64.90	27.43	72.32
**L^Bn^•Pd^I^**	2.2300(5)	1.848(2)	−22.15	45.921	51.731	12.92	87.00	43.84	55.51	37.551	60.288	35.08	64.26	24.04	75.63
**L^Allyl^•Pd^I^**	2.2311(6)	1.846(2)	−23.49	45.996	51.677	12.93	86.99	43.56	55.79	37.534	60.449	34.78	64.56	24.60	75.02
**L^CCPh^•Pd^I^**	2.2269(11)^d)^ 2.2319(11)^d)^	1.774(4)^d)^ 1.762(4)^d)^	−63.85	44.906	52.409	13.00	86.92	42.13	57.25	35.728	62.902	31.65	67.39	42.42	57.37

^a)^ For details of crystallographic parameters, see Supporting Information section IV.

^b)^ CD_2_Cl_2_, 298 K.

^c)^ Computed at PBE0/def2‐TZVP theory level. See Supporting Information section V for details.

^d)^ Two molecules of **L** ^CCPh^•Pd^I^ were found in each asymmetric unit cell.

**Figure 4:**
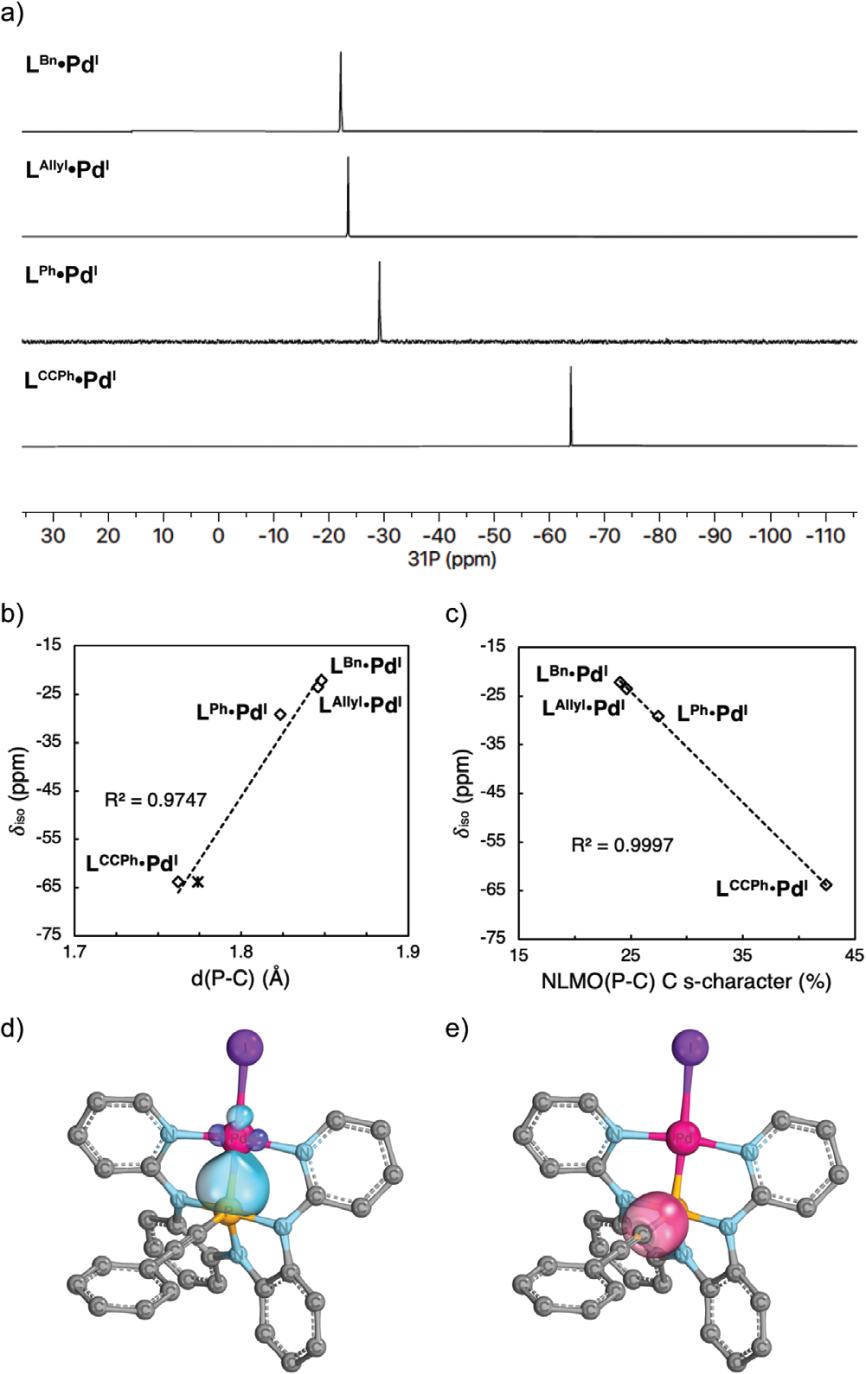
Effect of hydrocarbyl groups on the ^31^P NMR shifts of **L^R^•Pd^I^** (R = Allyl, Bn, Ph, CCPh) and NLMO depictions of selected bonds in **L^CCPh^•Pd^I^**. a) ^31^P NMR spectra of **L^R^•Pd^I^** (CD_2_Cl_2_, 298 K); b) ^31^P NMR shifts versus P─C bond distances. Note: Two molecules of **L^CCPh^•Pd^I^** were found in each asymmetric cell; c) ^31^P NMR shifts versus the s‐character of C atoms in the NLMOs representing P─C bonds; d) Pd─P bond orbital of **L^CCPh^•Pd^I^**; e) P─C bond orbital of **L^CCPh^•Pd^I^**. Each orbital isosurface, rendered with IBOview, represents 80% of the integrated electron density of the orbital.

In summary, we establish a general route to isolable palladaphosphoranes via intramolecular hydrocarbyl migration from Pd(II) to a constrained σ^3^‐P ligand. This transformation proceeds cleanly for C_sp_, C_sp_^2^, and C_sp_^3^ migrating fragments to furnish a family of (σ^4^‐P)─Pd complexes authenticated by multinuclear NMR and single‐crystal X‐ray diffraction. Across the resulting series, the κ^3^‐N,P,N scaffold buttresses an essentially invariant Pd─P bond distance that is preserved even under metal‐based substition reactions, implying opportunities for use of the as novel and robust pincer‐like support. Conceptually, these results provide evidence of an expanded role of P(III) ligands beyond passive σ‐donors, connecting the electrophilic reactivity of **L** to precedent with higher‐coordinate heavy group‐15 congeners.^[^ ^57^ ^]^ Given the modularity of the triamide framework and the variability of Pd‐bound substituents, the present results forecast developments enabling predictable control over hydrocarbyl migration. Conceptually, establishing controllable Pd─C ↔ P─C exchange with σ^3^‐P ligands reframes metal‐ligand migration from an off‐pathway scrambling or deactivation process into a designable element of Pd chemistry,^[^ ^58^, ^59^, ^60^, ^61^, ^62^, ^63^ ^]^ paving the way to nonspectator P‐ligand architectures that interface ligand‐centered reactivity with canonical palladium transformations in cross‐coupling and related catalytic chemistry.

## Conflict of Interests

The authors declare no conflict of interest.

## Supporting information



Supporting Information

Supporting Information

## Data Availability

The data that support the findings of this study are available in the Supporting Information of this article.
