# Supplementary material for: Csp, Csp 2, and Csp 3 Hydrocarbyl Group Migration from Pd(II) to P(III): Accessing Metallophosphoranes via Nonspectator Ligand Reactivity
Source: Angew Chem Int Ed Engl. 2025 Nov 13;65(2):e19496. doi: 10.1002/anie.202519496 (PMC12790345; doi:10.1002/anie.202519496)
Supplement: Supplementary file 1 — Supporting Information [file ANIE-65-e19496-s002.pdf]

Supporting Information for

C_{sp}, $C_{sp}{}^2$, and $C_{sp}{}^3$ Hydrocarbyl Group Migration from Pd(II) to P(III): Accessing Metallophosphoranes via Nonspectator Ligand Reactivity

Lily Ueh-Hsi Wang, Akira Tanushi, Peter Müller, Alexander T. Radosevich*

Department of Chemistry, Massachusetts Institute of Technology, 77 Massachusetts Avenue, Cambridge, Massachusetts 02139, United States

* email: radosevich@mit.edu

Table of Contents

I. General Considerations 3

II. Synthetic Procedures..4

III. Multinuclear NMR And IR Spectra of New Compounds..10

IV. Crystallographic Details..28

V. Computational Details for Natural Localized Molecular Orbital Analysis..46

a) Comparison of Crystallographic and Computed Structures..47
b) Cartesian Coordinates for Stationary Points..51
c) Natural Localized Molecular Orbital Analysis..63

VI. Details of Low Temperature In-Situ ^{31}P NMR Experiments..65

VII. Computational Details for Mechanistic Studies..67

a) Cartesian Coordinates for Stationary Points..68
b) Thermodynamic comparison of **L•Pd^{PhCI}** and **L•Pd^{PhCI}-iso**..77

VIII. References..78

I. General Considerations

All manipulations were carried out under an N_2 atmosphere using standard glovebox techniques unless otherwise stated. Diethyl ether (Et_2O), methylene chloride (CH_2Cl_2), tetrahydrofuran (THF), acetonitrile (MeCN), and pentane were purified and collected under Ar using a Glass Contour Solvent Purification System and solvents were further dried over 3 or 4 Å molecular sieves under an inert atmosphere in a glovebox. Deuterated methylene chloride (CD_2Cl_2) for NMR was purchased from Cambridge Isotopes Laboratories, Inc. and stored over 3 Å molecular sieves under an inert atmosphere in a glovebox. All chemicals were purchased from commercial sources (Sigma Aldrich, TCI, Alfa, Strem Chemicals) and used without further purification. Ligand **L**,[S1] *cis*-[(TMEDA)PdBr($CH_2C_6H_5$)],[S2] *cis*-[(TMEDA)PdI(C_6H_5)],[S3] and *trans*-[Pd(C≡CC_6H_5)Br(PPh_3)$_2$][S4] were synthesized according to literature procedures. All glassware was oven-dried at 120 °C prior to use.

NMR, IR, and mass spectra were obtained in the Department of Chemistry Instrumental Facility (DCIF) at the Massachusetts Institute of Technology. ^{1}H, ^{13}C{^{1}H}, ^{13}C{^{1}H}{^{31}P}, ^{31}P, and ^{31}P{^{1}H} NMR spectra were recorded on 400 or 500 MHz spectrometers at 298K. ^{1}H and ^{13}C chemical shifts are reported relative to residual solvent resonances.[S5] ^{31}P chemical shifts are absolutely referenced and reported versus phosphoric acid (0 ppm). IR spectra were recorded using solid samples on a Bruker Alpha II FTIR spectrometer with a diamond crystal ATR accessory. High-resolution mass spectrometry (HRMS) was performed at the Mass Spectrometry Laboratory within the DCIF using an Agilent QTOF 6545 with an ESI ionization source.

Low-temperature diffraction data were collected using Mo K_α radiation (λ = 0.71073 Å) on a Bruker-AXS Kappa Duo diffractometer with $I\mu S$ micro-sources, coupled to a Photon 3 CPAD detector, performing ϕ-and ω-scans. The structures were solved by dual-space methods using SHELXT and refined against F^2 on all data by full-matrix least squares with SHELXL-2017 following established refinement strategies.[S6–S8] Unless otherwise noted below, hydrogen atoms were included into the model at geometrically calculated positions and refined using a riding model. The isotropic displacement parameters of all hydrogen atoms were fixed to 1.2 times the U_{eq}-value of the atoms they are linked to (1.5 times for methyl groups). Details of the data quality and a summary of the residual values of the refinement are listed in Table S1 to S8.

II. Synthetic Procedures

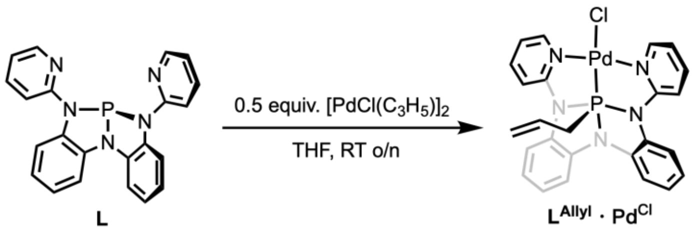

Synthesis of L^{Allyl}•Pd^{Cl}. In a N_2 glovebox, $[PdCl(C_3H_5)]_2$ (28.7 mg, 0.0784 mmol) was dissolved in THF (5 mL) and added a solution of **L** (60.0 mg, 0.157 mmol) in THF (5 mL), yielding an orange solution. After stirring overnight (~16h) at ambient temperature, the orange heterogeneous mixture was dried under vacuum. The bright orange residue was thoroughly washed with Et_2O (3 × 2 mL) and dried under vacuum to afford **L^{Allyl}•Pd^{Cl}** (72.0 mg, 81.1 % yield). X-ray quality crystals of **L^{Allyl}•Pd^{Cl}** were grown via vapor diffusion of Et_2O into a THF solution of **L^{Allyl}•Pd^{Cl}** at ambient temperature. **^{1}H NMR** (400 MHz, CD_2Cl_2) δ 9.37 (d, *J* = 6.1 Hz, 2H), 7.72 (tt, *J* = 7.0, 1.4 Hz, 2H), 7.63 (dd, *J* = 7.7, 1.6 Hz, 2H), 7.50 (d, *J* = 8.5 Hz, 2H), 7.33 (dd, *J* = 7.6, 1.6 Hz, 2H), 7.12 – 6.99 (m, 4H), 6.86 (ddd, *J* = 7.3, 6.0, 1.3 Hz, 2H), 5.52 (ddq, *J* = 17.0, 10.2, 7.5 Hz, 1H), 4.79 (ddd, *J* = 10.1, 5.6, 1.7 Hz, 1H), 4.64 – 4.52 (m, 1H), 2.84 (dd, *J* = 11.5, 7.7 Hz, 2H). **^{31}P{^{1}H} NMR** (162 MHz, CD_2Cl_2) δ -12.39. **^{31}P NMR** (162 MHz, CD_2Cl_2) δ -12.38. **^{13}C{^{1}H} NMR** (101 MHz, CD_2Cl_2) δ 152.9 (d, *J* = 13.7 Hz), 152.4, 140.4, 131.7, 131.5 (d, *J* = 8.4 Hz), 131.0 (d, *J* = 8.4 Hz), 122.6, 121.82, 119.0 (d, *J* = 15.9 Hz), 116.4, 113.6, 112.8 (d, *J* = 8.2 Hz), 110.4 (d, *J* = 4.2 Hz), 42.9 (d, *J* = 74.0 Hz). **HRMS** (ESI) m/z Calcd for $C_{25}H_{22}ClN_5PPd$ [M+H$^+$]: 564.0336, found 564.0331.

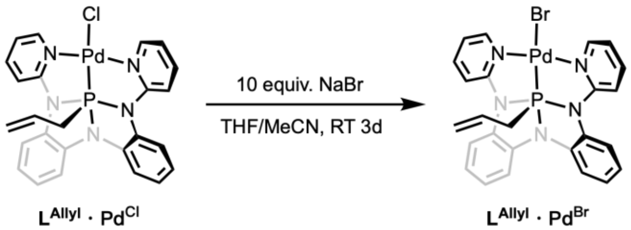

Synthesis of L^{Allyl}•Pd^{Br}. In a N_2 glovebox, **L^{Allyl}•Pd^{Cl}** (80.0 mg, 0.142 mmol) was dissolved in THF (5 mL) and added a heterogeneous suspension of NaBr (146 mg, 1.42 mmol) in MeCN (5 mL), yielding an orange chalky mixture. After stirring at ambient temperature for three days, the yellow heterogeneous mixture was dried under vacuum. The residue was extracted with CH_2Cl_2 (3 × 3 mL) and filtered through celite to remove the sodium salts. The filtrate was then dried under vacuum to afford an orange powder. The crude product was further purified by liquid-liquid diffusion of Et_2O into a CH_2Cl_2 solution of **L^{Allyl}•Pd^{Br}** at -20°C, yielding a yellow crystalline solid (yield 56.6 mg, 65.6%). X-ray quality crystals of **L^{Allyl}•Pd^{Br}** were grown via vapor diffusion of pentane into a THF solution of **L^{Allyl}•Pd^{Br}** at ambient temperature. **^{1}H NMR** (500

MHz, CD_2Cl_2) δ 9.56 (d, *J* = 6.1 Hz, 2H), 7.73 (t, *J* = 7.8 Hz, 2H), 7.63 (d, *J* = 7.8 Hz, 2H), 7.50 (d, *J* = 8.5 Hz, 2H), 7.33 (d, *J* = 7.7 Hz, 2H), 7.07 (dt, *J* = 24.2, 7.6 Hz, 4H), 6.84 (t, *J* = 6.6 Hz, 2H), 5.59 – 5.45 (m, 1H), 4.79 (dd, *J* = 10.1, 5.6 Hz, 1H), 4.56 (dd, *J* = 17.0, 6.9 Hz, 1H), 2.82 (dd, *J* = 11.5, 7.7 Hz, 2H). **^{31}P{^{1}H} NMR** (203 MHz, CD_2Cl_2) δ -15.67. **^{31}P NMR** (203 MHz, CD_2Cl_2) δ -15.67. **^{13}C{^{1}H}** NMR (126 MHz, CD_2Cl_2) δ 154.3, 153.0 (d, *J* = 13.7 Hz), 140.6, 131.6, 131.5 (d, *J* = 8.6 Hz), 130.9 (d, *J* = 8.4 Hz), 122.8, 121.9, 119.09 (d, *J* = 16.0 Hz), 116.6, 113.9, 112.8 (d, *J* = 8.3 Hz), 110.3 (d, *J* = 4.3 Hz), 42.4 (d, *J* = 74.0 Hz). **HRMS** (ESI) m/z Calcd for $C_{25}H_{22}BrN_5PPd$ [$M+H^+$]: 607.9831, found 607.9824.

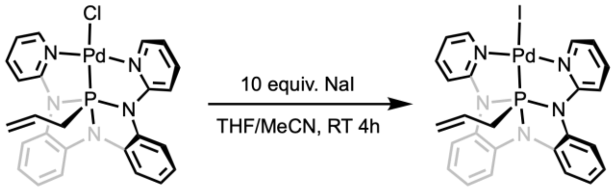

L^{Allyl} · Pd^{Cl} L^{Allyl} · Pd^{I}

Synthesis of L^{Allyl}•Pd^I. In a N_2 glovebox, **L^{Allyl}•Pd^{Cl}** (80.0 mg, 0.142 mmol) was dissolved in THF (5 mL) and added a solution of NaI (212 mg, 1.42 mmol) in MeCN (5 mL), yielding an orange chalky mixture. After stirring at ambient temperature for 4 h, the orange heterogeneous mixture was dried under vacuum. The residue was extracted with CH_2Cl_2 (3 × 3 mL) and filtered through celite to remove the sodium salts. The filtrate was then dried under vacuum to afford an orangish red powder. The crude product was further purified by liquid-liquid diffusion of Et_2O into a CH_2Cl_2 solution of **L^{Allyl}•Pd^I** at -20 °C, yielding a red crystalline solid (yield 65.2 mg, 70.1 %). X-ray quality crystals of **L^{Allyl}•Pd^I** were grown via vapor diffusion of pentane into a THF solution of **L^{Allyl}•Pd^I** at ambient temperature. **^{1}H NMR** (500 MHz, CD_2Cl_2) δ 9.80 (d, *J* = 6.0 Hz, 2H), 7.75 (t, *J* = 7.8 Hz, 2H), 7.63 (d, *J* = 7.8 Hz, 2H), 7.49 (d, *J* = 8.4 Hz, 2H), 7.34 (d, *J* = 7.7 Hz, 2H), 7.10 (t, *J* = 7.7 Hz, 2H), 7.05 (t, *J* = 7.6 Hz, 2H), 6.80 (t, *J* = 6.6 Hz, 2H), 5.51 (tq, *J* = 14.6, 7.3 Hz, 1H), 4.80 (dd, *J* = 10.1, 5.6 Hz, 1H), 4.54 (dd, *J* = 16.9, 6.9 Hz, 1H), 2.79 (dd, *J* = 11.4, 7.7 Hz, 2H). **^{31}P{^{1}H} NMR** (203 MHz, CD_2Cl_2) δ -23.49. **^{31}P NMR** (203 MHz, CD_2Cl_2) δ -23.49. **^{13}C{^{1}H}** NMR (126 MHz, CD_2Cl_2) δ 157.3, 153.1 (d, *J* = 13.9 Hz), 140.8, 131.5, 131.4 (d, *J* = 8.6 Hz), 130.6 (d, *J* = 8.4 Hz), 123.0, 121.9, 119.2 (d, *J* = 15.8 Hz), 116.6, 114.1, 112.8 (d, *J* = 8.3 Hz), 110.4 (d, *J* = 4.5 Hz), 41.7 (d, *J* = 72.9 Hz). **HRMS** (ESI) m/z Calcd for $C_{25}H_{22}IN_5PPd$ [$M+H^+$]: 655.9692, found 655.9702.

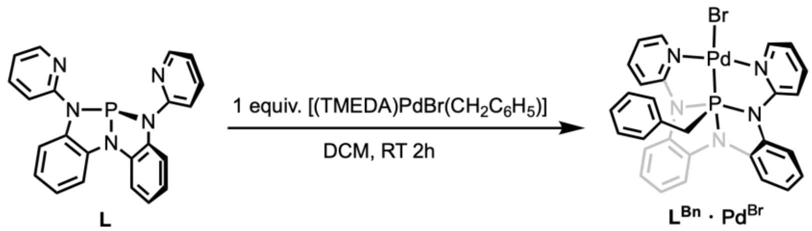

L **L^Bn · Pd^Br**

Synthesis of L^Bn•Pd^Br. In a N_2 glovebox, [(TMEDA)PdBr($CH_2C_6H_5$)] (41.3 mg, 0.105 mmol) was dissolved in CH_2Cl_2 (3 mL) and added a CH_2Cl_2 solution (2 mL) of **L** (40.0 mg, 0.105 mmol), yielding an orange solution. After stirring at ambient temperature for 2 h, the orange solution was concentrated to ~ 0.5 mL. Pentane (15 mL) was added dropwise to induce precipitation of an orange solid, which was collected by filtration and dried under vacuum to afford **L^Bn•Pd^Br** (yield 66.0 mg, 95.5%). The product thus obtained contained residual TMEDA in some synthetic runs but could be further purified by liquid-liquid diffusion of Et_2O into a CH_2Cl_2 solution of **L^Bn•Pd^Br** at -20°C. X-ray quality crystals of **L^Bn•Pd^Br** were grown via vapor diffusion of pentane into a THF solution of **L^Bn•Pd^Br** at room temperature. **^{1}H NMR** (400 MHz, CD_2Cl_2) δ 9.53 (d, *J* = 6.0 Hz, 2H), 7.67 – 7.58 (m, 4H), 7.33 – 7.25 (m, 4H), 7.12 – 7.00 (m, 4H), 6.99 – 6.96 (m, 1H), 6.92 (t, *J* = 7.5 Hz, 2H), 6.81 (t, *J* = 6.6 Hz, 2H), 6.60 (dd, *J* = 7.8, 3.1 Hz, 2H), 3.45 (d, *J* = 11.2 Hz, 2H). **^{31}P{^{1}H} NMR** (162 MHz, CD_2Cl_2) δ -14.45. **^{31}P NMR** (162 MHz, CD_2Cl_2) δ -14.45. **^{13}C{^{1}H} NMR** (101 MHz, CD_2Cl_2) δ 154.2, 152.7 (d, *J* = 13.8 Hz), 140.4, 134.9 (d, *J* = 6.9 Hz), 131.7, 131.2 (d, *J* = 8.6 Hz), 129.6 (d, *J* = 6.8 Hz), 128.3 (d, *J* = 3.7 Hz), 126.5 (d, *J* = 4.5 Hz), 122.7, 121.9, 116.6, 113.7, 112.9 (d, *J* = 8.2 Hz), 110.4 (d, *J* = 4.1 Hz), 44.1 (d, *J* = 69.8 Hz). **HRMS** (ESI) m/z Calcd for $C_{29}H_{24}BrN_5PPd$ [M+H$^+$]: 657.9988, found 657.9972.

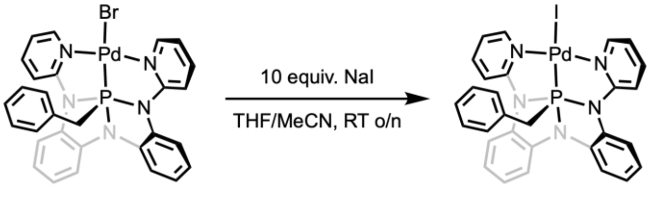

L^Bn · Pd^Br **L^Bn · Pd^I**

Synthesis of L^Bn•Pd^I. In a N_2 glovebox, **L^Bn•Pd^Br** (85.0 mg, 0.130 mmol) was dissolved in THF (5 mL), and added a solution of NaI (193 mg, 1.29 mmol) in MeCN (5 mL) yielding an orange chalky mixture. After stirring at ambient temperature overnight, the solvent from orange heterogeneous mixture was removed under reduced pressure. The residue was extracted with CH_2Cl_2 (3 × 3 mL) and filtered through celite to remove the sodium salts. The filtrate was then dried under vacuum to afford an orangish red powder. The crude product was further purified by liquid-liquid diffusion of Et_2O into a CH_2Cl_2 solution of **L^Bn•Pd^I** at -20 °C, yielding a red crystalline solid (yield 62.6 mg, 68.7 %). X-ray quality crystals of **L^Bn•Pd^I** was

grown via vapor diffusion of pentane into a THF/DCM (THF:DCM ~ 1:1) solution of **L^{Bn}•Pd^{I}** at ambient temperature. **^{1}H NMR** (500 MHz, CD_2Cl_2) δ 9.76 (d, *J* = 6.2 Hz, 2H), 7.65 (t, *J* = 7.8 Hz, 2H), 7.62 (d, *J* = 7.8 Hz, 2H), 7.29 (d, *J* = 3.0 Hz, 2H), 7.27 (d, *J* = 3.9 Hz, 2H), 7.09 (t, *J* = 7.6 Hz, 2H), 7.04 (t, *J* = 7.6 Hz, 2H), 7.01 – 6.95 (m, 1H), 6.92 (t, *J* = 7.5 Hz, 2H), 6.78 (t, *J* = 6.6 Hz, 2H), 6.58 (dd, *J* = 7.7, 2.9 Hz, 2H), 3.41 (d, *J* = 11.1 Hz, 2H). **^{31}P{^{1}H} NMR** (203 MHz, CD_2Cl_2) δ -22.15. **^{31}P NMR** (203 MHz, CD_2Cl_2) δ -22.15. **^{13}C{^{1}H} NMR** (126 MHz, CD_2Cl_2) δ 157.2, 152.7 (d, *J* = 13.8 Hz), 140.7, 134.7 (d, *J* = 6.8 Hz), 131.6, 131.1 (d, *J* = 8.6 Hz), 129.6 (d, *J* = 6.9 Hz), 128.3 (d, *J* = 3.6 Hz), 126.6 (d, *J* = 4.4 Hz), 122.9, 121.9, 116.7, 113.9, 112.8 (d, *J* = 8.2 Hz), 110.6 (d, *J* = 4.3 Hz), 43.5 (d, *J* = 68.7 Hz). **HRMS** (ESI) m/z Calcd for $C_{29}H_{24}IN_5PPd$ [M+H^{+}]: 705.9849, found 705.9861.

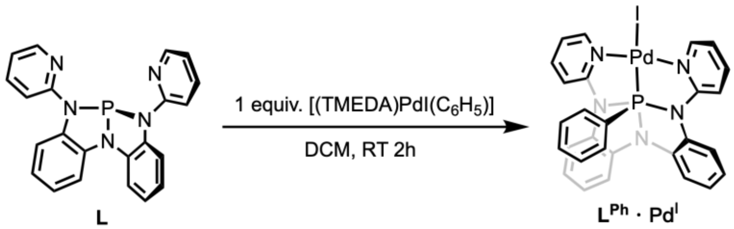

Synthesis of L^{Ph}•Pd^{I}. In a N_2 glovebox, [(TMEDA)PdI(C_6H_5)] (32.4 mg, 0.0760 mmol) was dissolved in CH_2Cl_2 (3 mL) and added a CH_2Cl_2 solution (3 mL) of **L** (29.0 mg, 0.0760 mmol), yielding an orange solution. After stirring at ambient temperature for 2 h, the orangish-red solution was concentrated to ~ 0.5 mL. Pentane (15 mL) was added dropwise to induce precipitation of an orange powder, which was collected by vacuum filtration on a glass frit, then dried under vacuum to afford **L^{Ph}•Pd^{I}** (yield 44.1 mg, 83.8 %). The product thus obtained contained residual TMEDA in some synthetic runs but could be further purified by liquid-liquid diffusion of Et_2O into a CH_2Cl_2 solution of **L^{Ph}•Pd^{I}** at -20 °C. X-ray quality crystals of **L^{Ph}•Pd^{I}** were grown via vapor diffusion of pentane into a THF solution of **L^{Ph}•Pd^{I}** at ambient temperature. **^{1}H NMR** (400 MHz, CD_2Cl_2) δ 9.76 (s, 2H), 7.67 (d, *J* = 7.7 Hz, 5H), 7.63 (d, *J* = 6.8 Hz, 1H), 7.44 (d, *J* = 8.4 Hz, 2H), 7.26 (dd, *J* = 7.9, 1.4 Hz, 2H), 7.17 – 7.12 (m, 1H), 7.12 – 7.05 (m, 4H), 6.99 (td, *J* = 7.7, 1.3 Hz, 2H), 6.73 (t, *J* = 6.6 Hz, 2H). **^{31}P{^{1}H} NMR** (162 MHz, CD_2Cl_2) δ -29.18. **^{31}P NMR** (162 MHz, CD_2Cl_2) δ -29.20. **^{13}C{^{1}H} NMR** (126 MHz, CD_2Cl_2) δ 157.38, 152.94 (d, *J* = 15.1 Hz), 140.76, 138.00 (d, *J* = 108.9 Hz), 131.41 (d, *J* = 9.6 Hz), 131.36, 130.69 (d, *J* = 16.1 Hz), 130.49 (d, *J* = 3.3 Hz), 128.18 (d, *J* = 14.9 Hz), 122.97, 121.88, 116.55, 114.23, 112.68 (d, *J* = 8.4 Hz), 110.32 (d, *J* = 4.6 Hz). **^{13}C{^{1}H}{^{31}P} NMR** (126 MHz, CD_2Cl_2) δ 157.40, 152.96, 140.76, 138.01, 131.43, 131.37, 130.70, 130.50, 128.19, 122.98, 121.88, 116.56, 114.24, 112.69, 110.32. **HRMS** (ESI) m/z Calcd for $C_{28}H_{22}IN_5PPd$ [M+H^{+}]: 691.9692, found 691.9697.

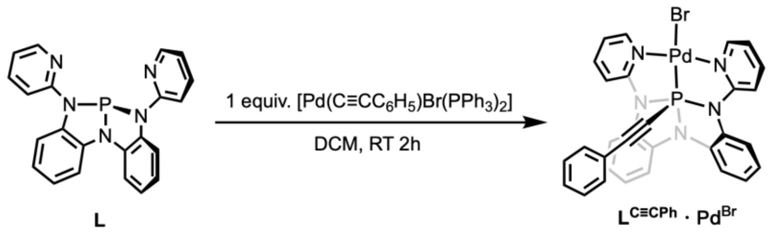

Synthesis of L^{CCPh}•Pd^{Br}. In a N_2 glovebox, *trans*-[Pd(C≡CC$_6$H$_5$)Br(PPh$_3$)$_2$] (100 mg, 0.123 mmol) was dissolved in CH_2Cl_2 (3 mL) and added a solution of **L**(46.9 mg, 0.123 mmol) in CH_2Cl_2 (2 mL), yielding a yellow solution. After stirring at ambient temperature for 2 h, the yellow solution was concentrated to ~ 0.5 mL. Et_2O (8 mL) and pentane (8 mL) were added dropwise sequentially to induce precipitation of a pale-yellow powder, which was collected by vacuum filtration on a glass frit, then dried under vacuum to afford **L^{CCPh}•Pd^{Br}** (yield 77.0 mg, 93.6%). The product thus obtained contained residual triphenylphosphine in some synthetic runs but could be further purified by liquid-liquid diffusion of Et_2O into a CH_2Cl_2 solution **L^{CCPh}•Pd^{Br}** at -20 °C. X-ray quality crystals of **L^{CCPh}•Pd^{Br}** were grown via vapor diffusion of pentane into a THF solution of **L^{CCPh}•Pd^{Br}** at ambient temperature. **^{1}H NMR** (500 MHz, CD_2Cl_2) δ 9.61 (d, *J* = 6.1 Hz, 2H), 7.78 (ddt, *J* = 8.6, 7.0, 1.6 Hz, 2H), 7.68 (dd, *J* = 7.6, 1.6 Hz, 2H), 7.57 (d, *J* = 8.4 Hz, 2H), 7.40 (dd, *J* = 7.5, 1.7 Hz, 2H), 7.29 – 7.21 (m, 1H), 7.19 – 7.06 (m, 6H), 7.04 – 6.99 (m, 2H), 6.91 – 6.85 (m, 2H). **^{31}P{^{1}H} NMR** (162 MHz, CD_2Cl_2) δ -55.73. **^{31}P NMR** (162 MHz, CD_2Cl_2) δ -55.73. **^{13}C{^{1}H}** NMR (126 MHz, CD_2Cl_2) δ 154.3, 152.4 (d, *J* = 17.6 Hz), 140.8, 132.3 (d, *J* = 1.5 Hz), 131.3, 130.8 (d, *J* = 10.3 Hz), 129.9, 128.6, 123.0, 122.2, 121.1 (d, *J* = 3.8 Hz), 116.8, 114.2, 113.1 (d, *J* = 9.5 Hz), 110.5 (d, *J* = 5.9 Hz), 96.7 (d, *J* = 34.9 Hz), 85.1 (d, *J* = 178.6 Hz). **IR**(ATR): *ν*(C≡C) 2166.38 cm^{-1} **HRMS** (ESI) m/z Calcd for $C_{30}H_{22}BrN_5PPd$ [M+H^{+}]: 667.9831, found 667.9826.

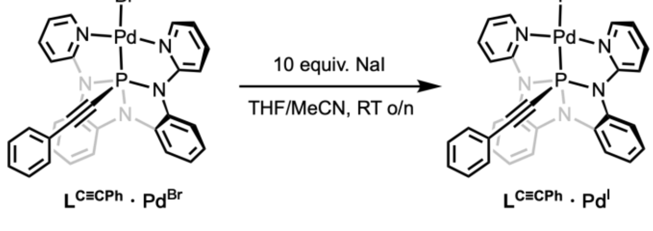

Synthesis of L^{CCPh}•Pd^{I}. In a N_2 glovebox, **L^{CCPh}•Pd^{Br}** (85 mg, 0.127 mmol) was dissolved in THF (5 mL), and added a solution of NaI (190 mg, 1.27 mmol) in MeCN (5 mL), yielding an orange chalky mixture. After stirring at ambient temperature overnight, the solvent from orange heterogeneous mixture was removed under vacuum. The residue was extracted with CH_2Cl_2 (3 × 3 mL) and filtered through celite to remove the sodium salts. The filtrate was then dried under vacuum to afford an orangish powder. The crude

product was further purified by liquid-liquid diffusion of Et_2O into a CH_2Cl_2 solution of $\mathbf{L^{CCPh}{\bullet}Pd^{I}}$ at -20 °C, yielding a red crystalline solid (yield 70.0 mg, 76.9 %). X-ray quality crystals of $\mathbf{L^{CCPh}{\bullet}Pd^{I}}$ were grown via vapor diffusion of pentane into a THF/DCM (THF:DCM ~ 1:1) solution of $\mathbf{L^{CCPh}{\bullet}Pd^{I}}$ at ambient temperature. **^{1}H** NMR (500 MHz, CD_2Cl_2) δ 9.85 (d, *J* = 6.1 Hz, 2H), 7.79 (t, *J* = 7.9 Hz, 2H), 7.68 (d, *J* = 7.6 Hz, 2H), 7.56 (d, *J* = 8.4 Hz, 2H), 7.40 (d, *J* = 7.5 Hz, 2H), 7.26 (t, *J* = 7.5 Hz, 1H), 7.15 (q, *J* = 6.9 Hz, 4H), 7.10 (t, *J* = 7.8 Hz, 2H), 7.00 (d, *J* = 7.7 Hz, 2H), 6.83 (t, *J* = 6.6 Hz, 2H). **^{31}P{^{1}H}** NMR (203 MHz, CD_2Cl_2) δ -63.85. **^{31}P** NMR (203 MHz, CD_2Cl_2) δ -63.85. **^{13}C{^{1}H}** NMR (126 MHz, CD_2Cl_2) δ 157.5 (d, *J* = 1.6 Hz), 152.6 (d, *J* = 17.9 Hz), 141.1, 132.3 (d, *J* = 1.7 Hz), 131.3, 130.8 (d, *J* = 10.5 Hz), 130.0, 128.6, 123.2, 122.3, 121.1 (d, *J* = 3.9 Hz), 116.9, 114.5, 113.1 (d, *J* = 9.6 Hz), 110.5 (d, *J* = 6.0 Hz), 97.0 (d, *J* = 34.5 Hz), 84.6 (d, *J* = 177.4 Hz). **IR**(ATR): ν(C≡C) 2168.38 cm^{-1}. **HRMS** (ESI) m/z Calcd for $C_{30}H_{22}IN_5PPd$ [$M+H^+$]: 715.9692, found 715.9706.

III. Multinuclear NMR and IR Spectra of New Compounds

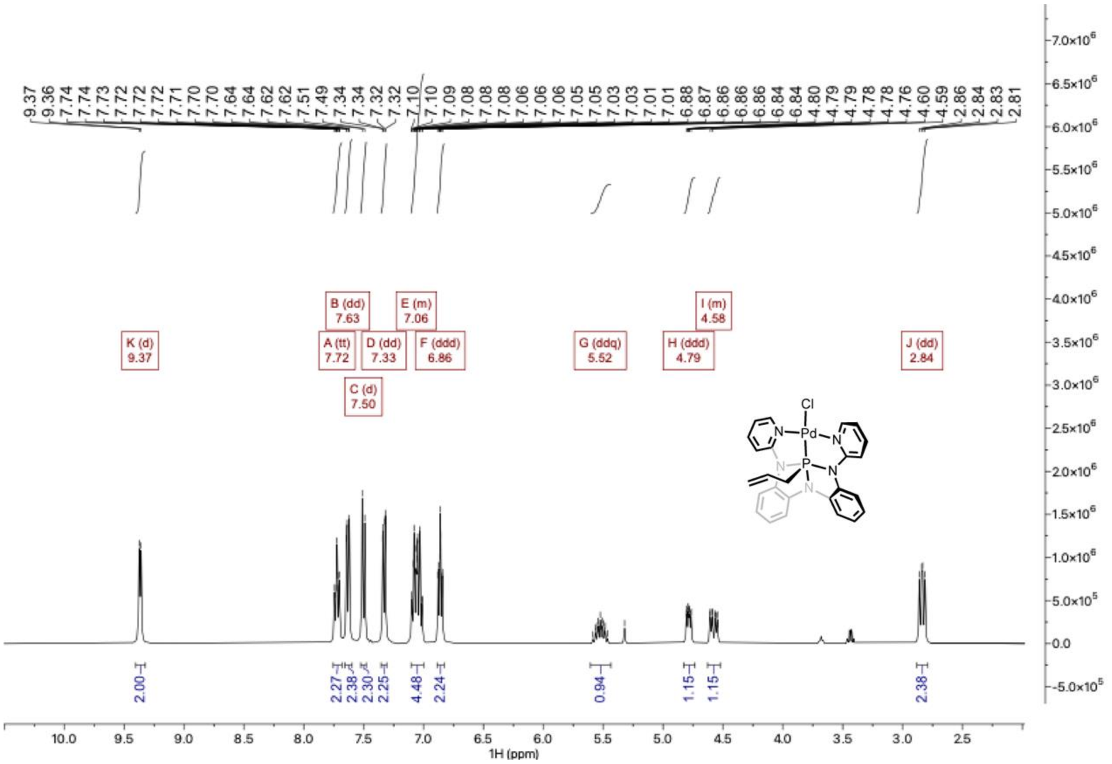

Figure S1. ^{1}H NMR spectrum of **L^{Allyl}•Pd^{Cl}** (400 MHz, CD_2Cl_2).

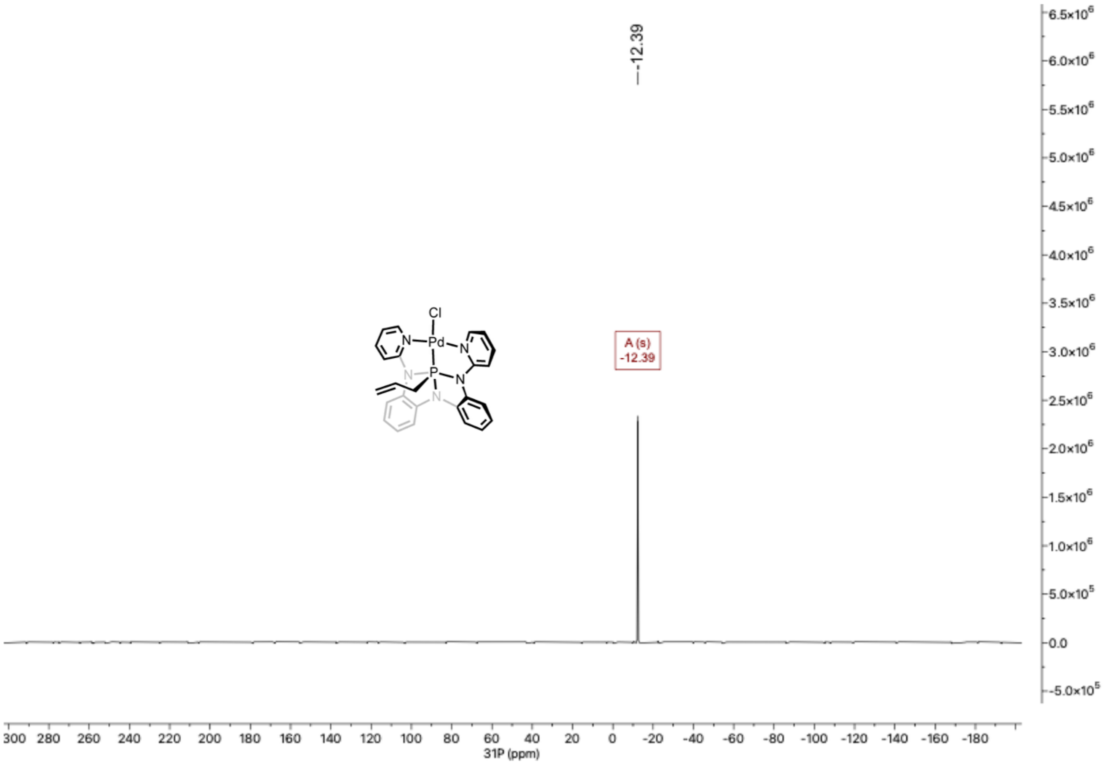

Figure S2. ^{31}P{^{1}H} NMR spectrum of **L^{Allyl}•Pd^{Cl}** (162 MHz, CD_2Cl_2).

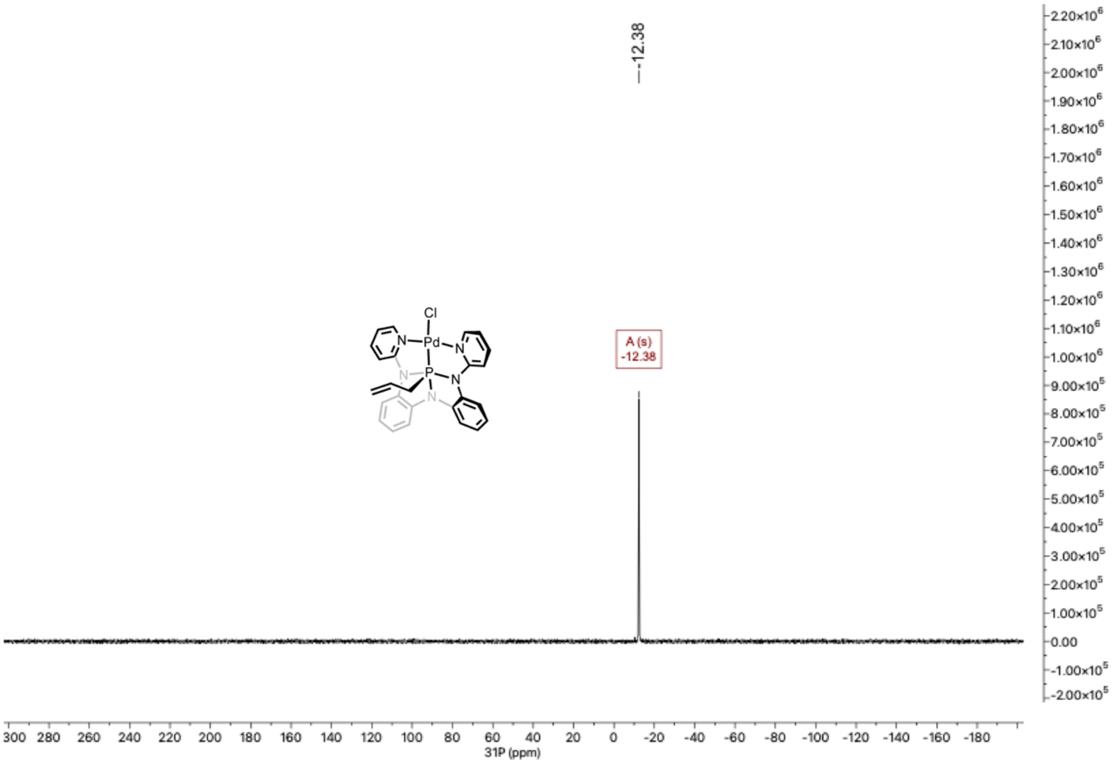

Figure S3. ^{31}P NMR spectrum of **L^{Allyl}•Pd^{Cl}** (162 MHz, CD_2Cl_2).

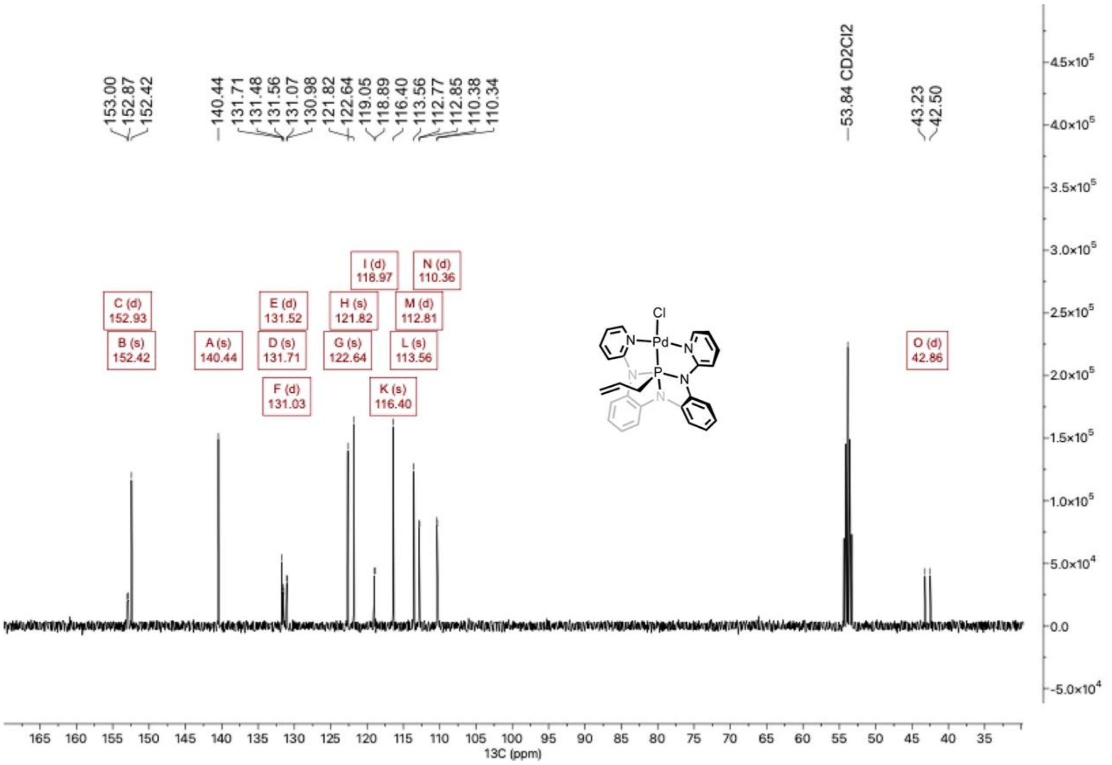

Figure S4. $^{13}C\{^1H\}$ NMR spectrum of **L^{Allyl}•Pd^{Cl}** (126 MHz, CD_2Cl_2).

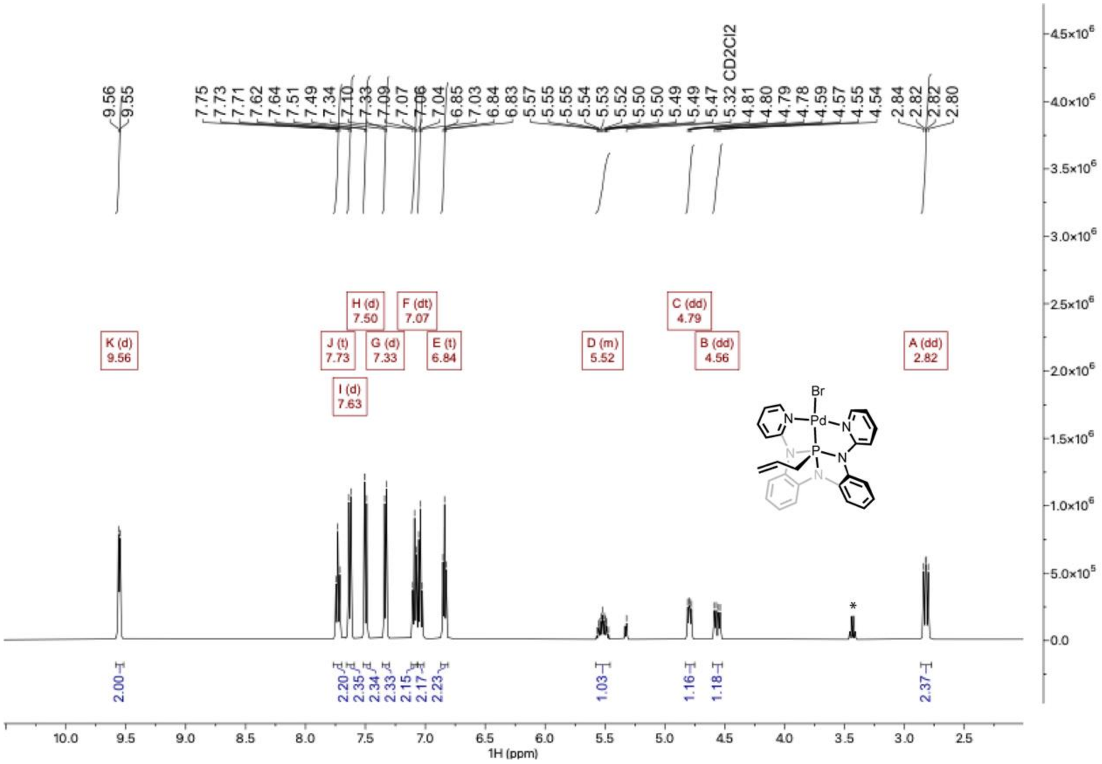

Figure S5. ^{1}H NMR spectrum of $\mathbf{L^{Allyl}{\bullet}Pd^{Br}}$ (400 MHz, CD_2Cl_2). * The Et_2O signal at 3.43 ppm is from the solvent co-crystallized in the crystalline sample.

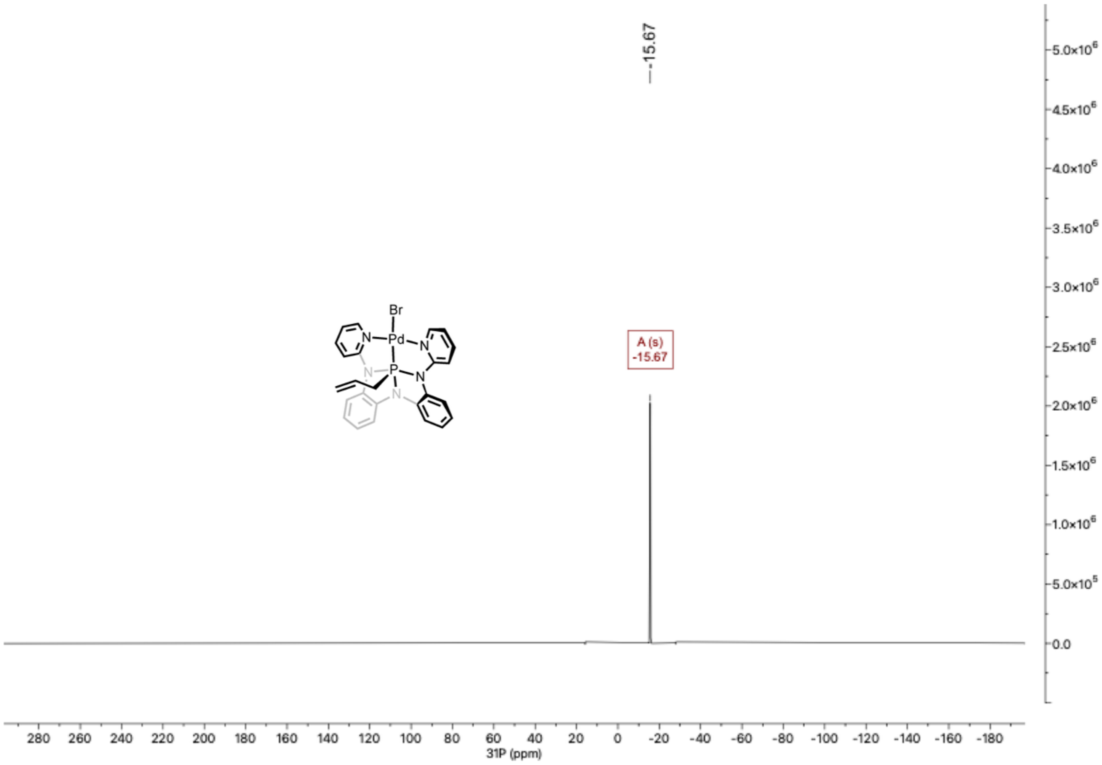

Figure S6. $^{31}P\{^{1}H\}$ NMR spectrum of $\mathbf{L^{Allyl}{\bullet}Pd^{Br}}$ (162 MHz, CD_2Cl_2).

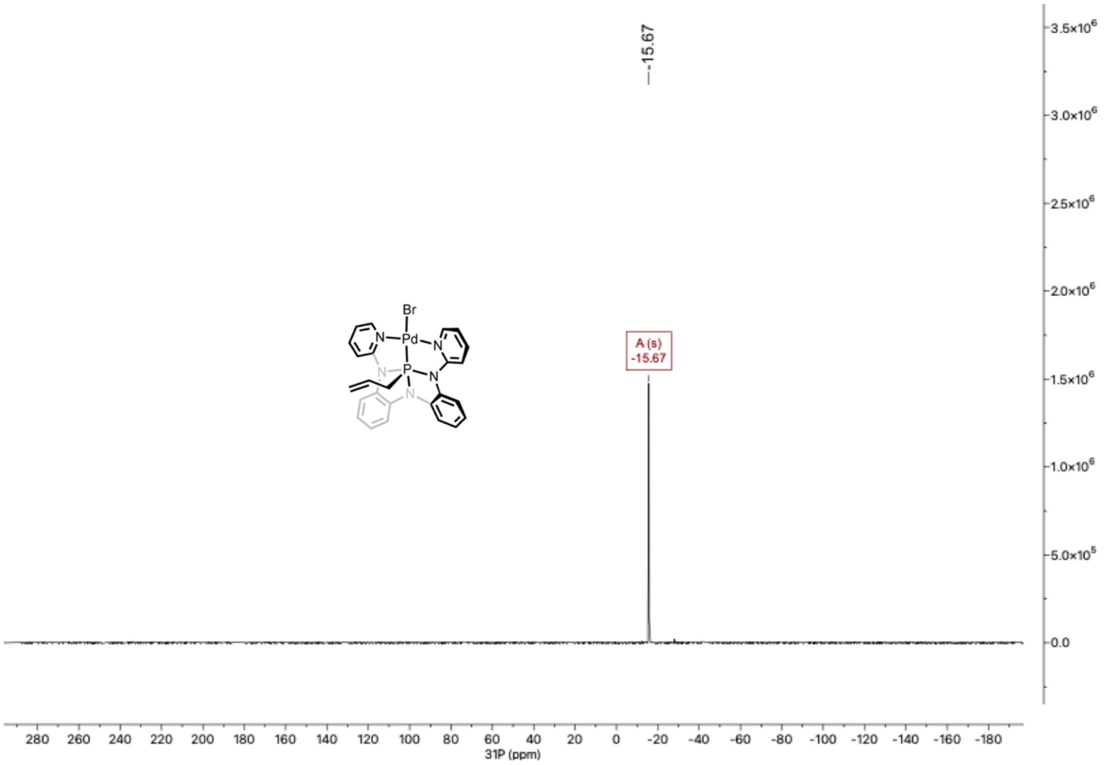

Figure S7. ^{31}P NMR spectrum of $\mathbf{L^{Allyl}{\bullet}Pd^{Br}}$ (162 MHz, CD_2Cl_2).

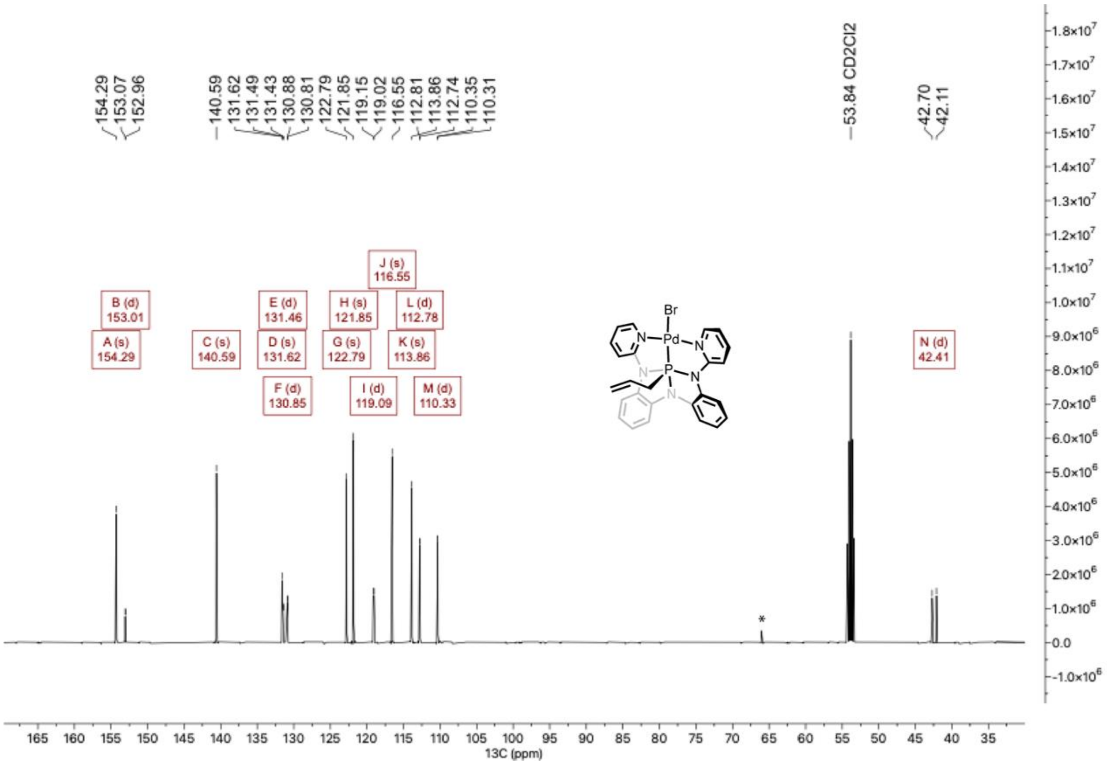

Figure S8. $^{13}C\{^1H\}$ NMR spectrum of $\mathbf{L^{Allyl}{\bullet}Pd^{Br}}$ (126 MHz, CD_2Cl_2). * The Et_2O signal at 66.11 ppm is from the solvent co-crystallized in the crystalline sample.

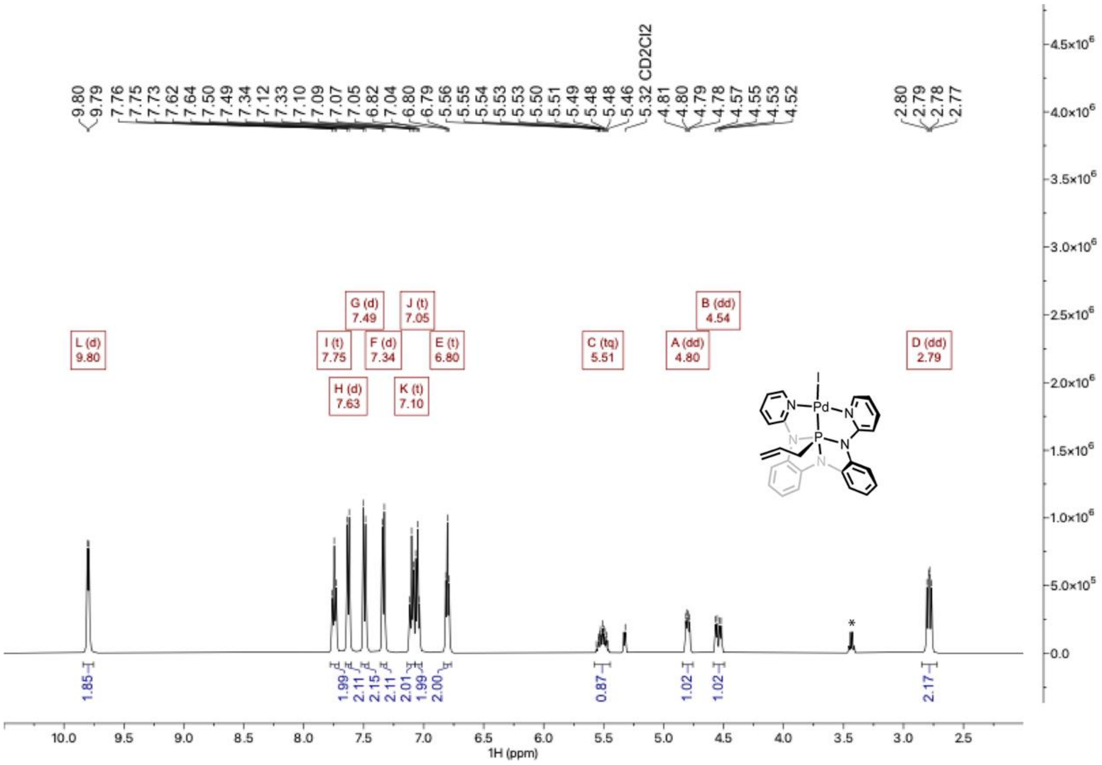

Figure S9. ^{1}H NMR spectrum of **L^{Allyl}•Pd^{I}** (400 MHz, CD_2Cl_2). * The Et_2O signal at 3.43 ppm is from the solvent co-crystallized in the crystalline sample.

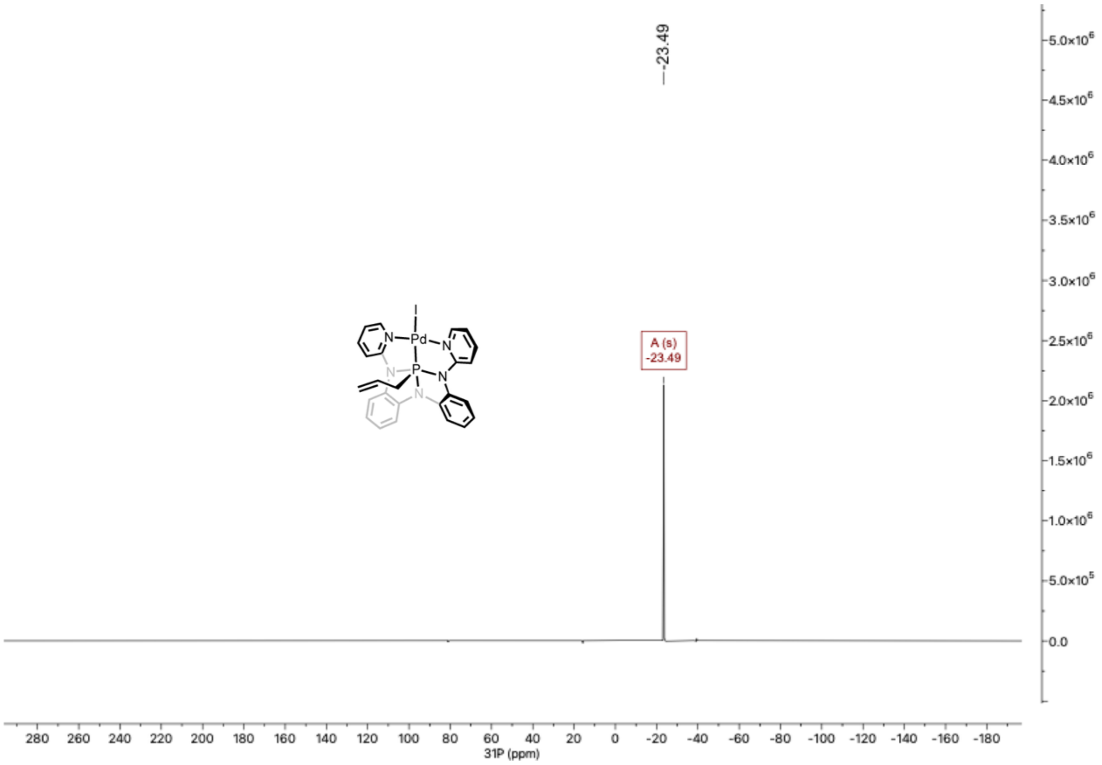

Figure S10. ^{31}P{^{1}H} NMR spectrum of **L^{Allyl}•Pd^{I}** (162 MHz, CD_2Cl_2).

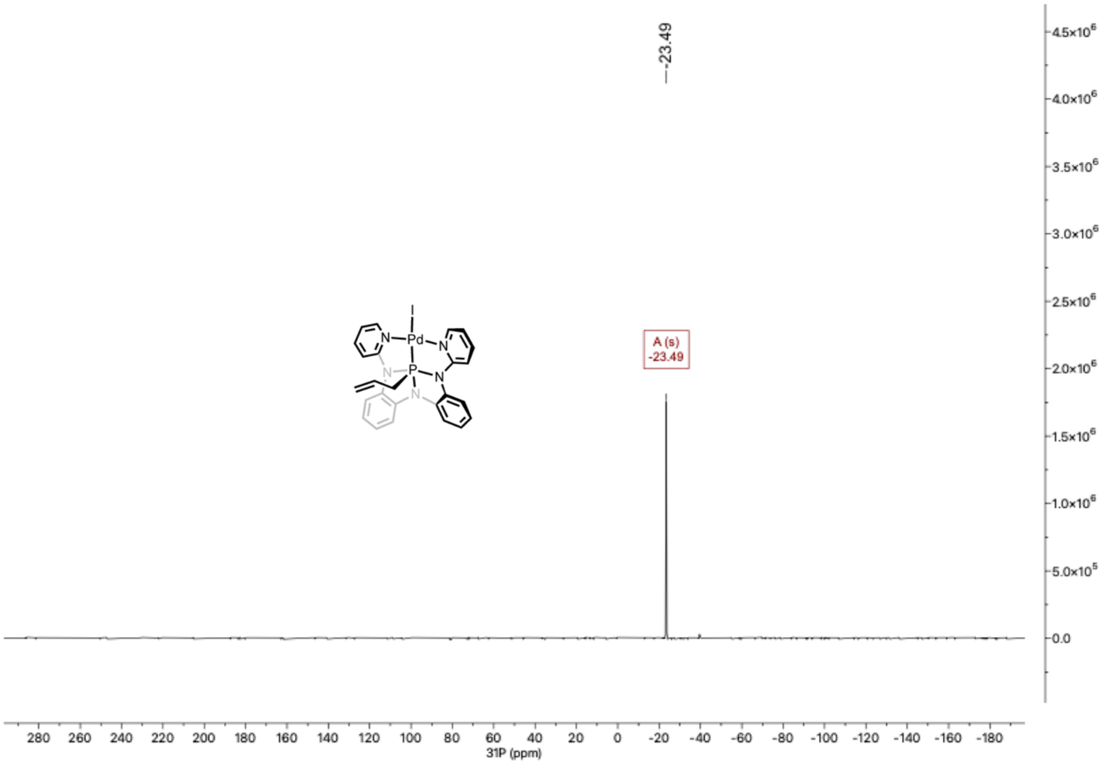

Figure S11. ^{31}P NMR spectrum of $\mathbf{L^{Allyl}{\bullet}Pd^{I}}$ (162 MHz, CD_2Cl_2).

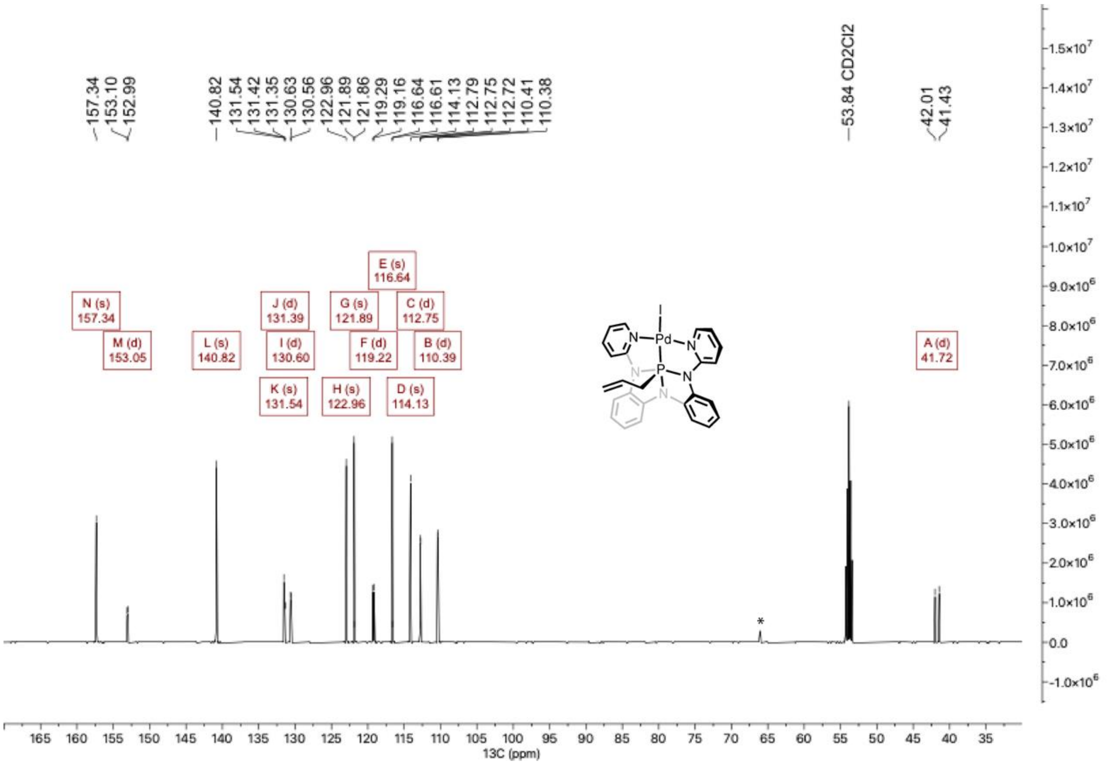

Figure S12. $^{13}C\{^{1}H\}$ NMR spectrum of $\mathbf{L^{Allyl}{\bullet}Pd^{I}}$ (126 MHz, CD_2Cl_2). * The Et_2O signal at 66.11 ppm is from the solvent co-crystallized in the crystalline sample.

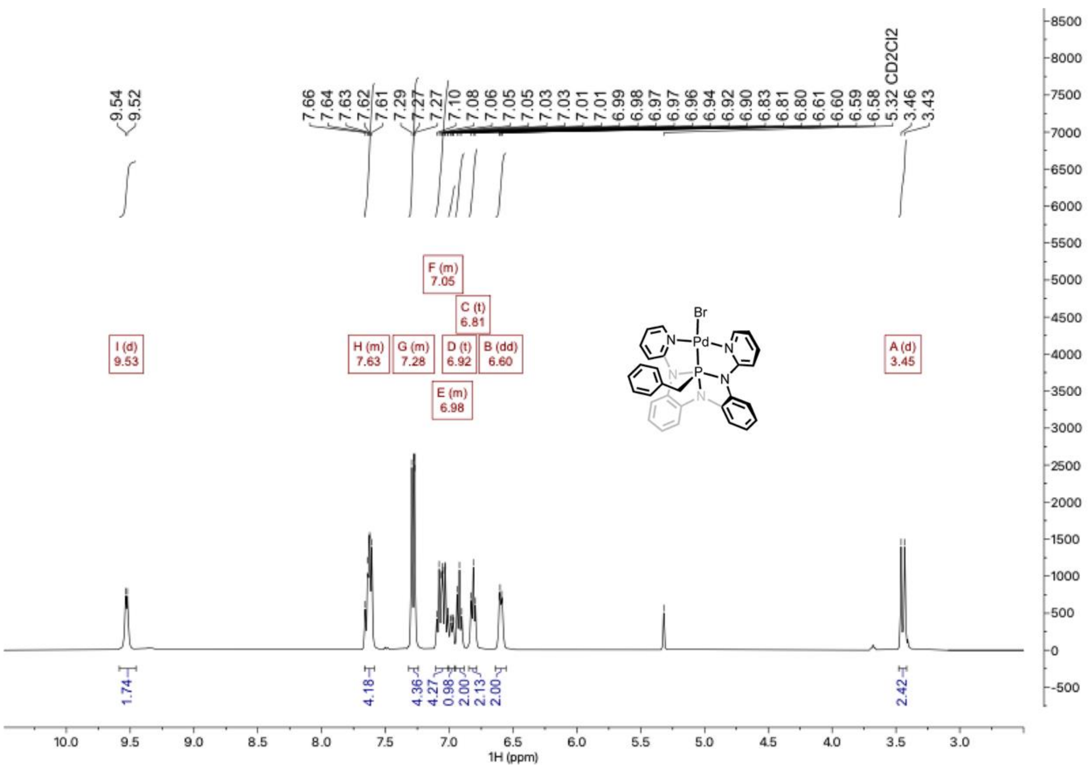

Figure S13. ^{1}H NMR spectrum of $\mathbf{L^{Bn}{\bullet}Pd^{Br}}$ (400 MHz, CD_2Cl_2).

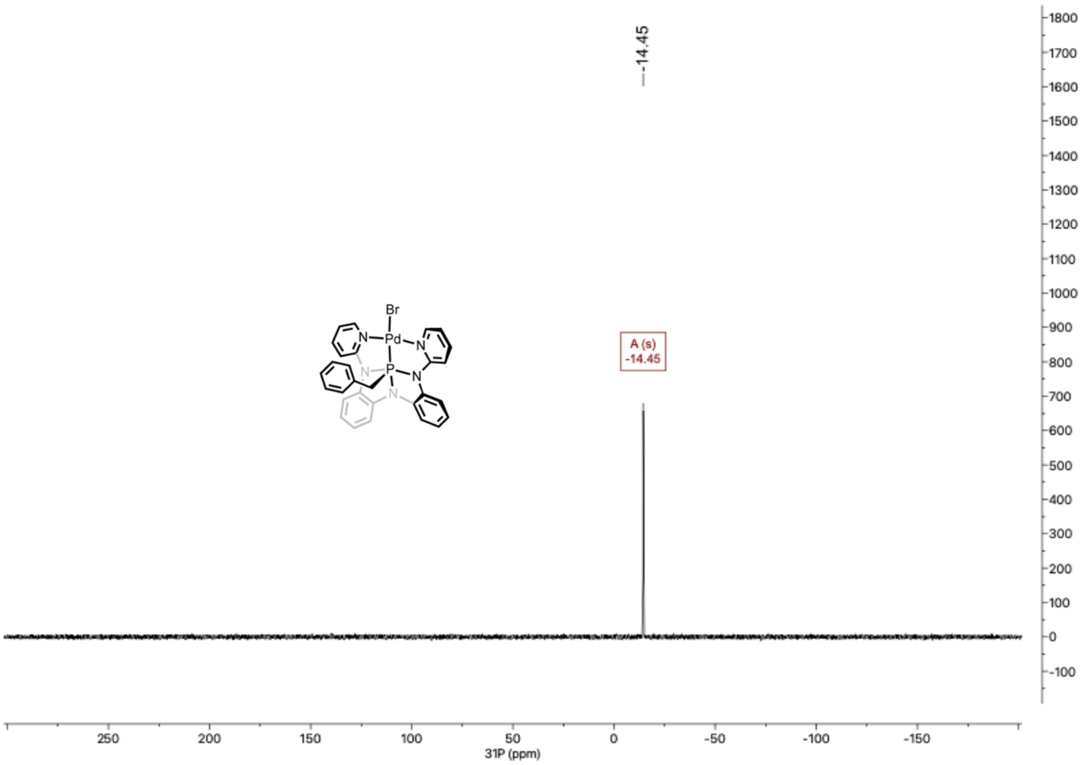

Figure S14. $^{31}P\{^{1}H\}$ NMR spectrum of $\mathbf{L^{Bn}{\bullet}Pd^{Br}}$ (162 MHz, CD_2Cl_2).

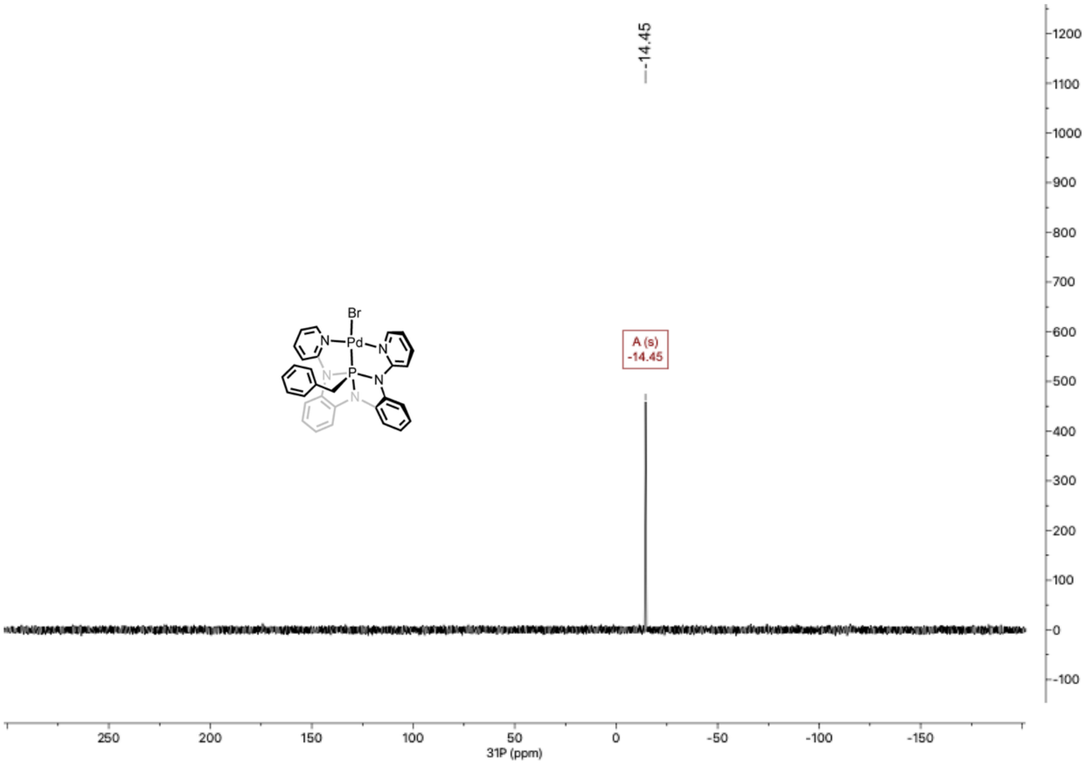

Figure S15. ^{31}P NMR spectrum of $\mathbf{L^{Bn}{\bullet}Pd^{Br}}$ (162 MHz, CD_2Cl_2).

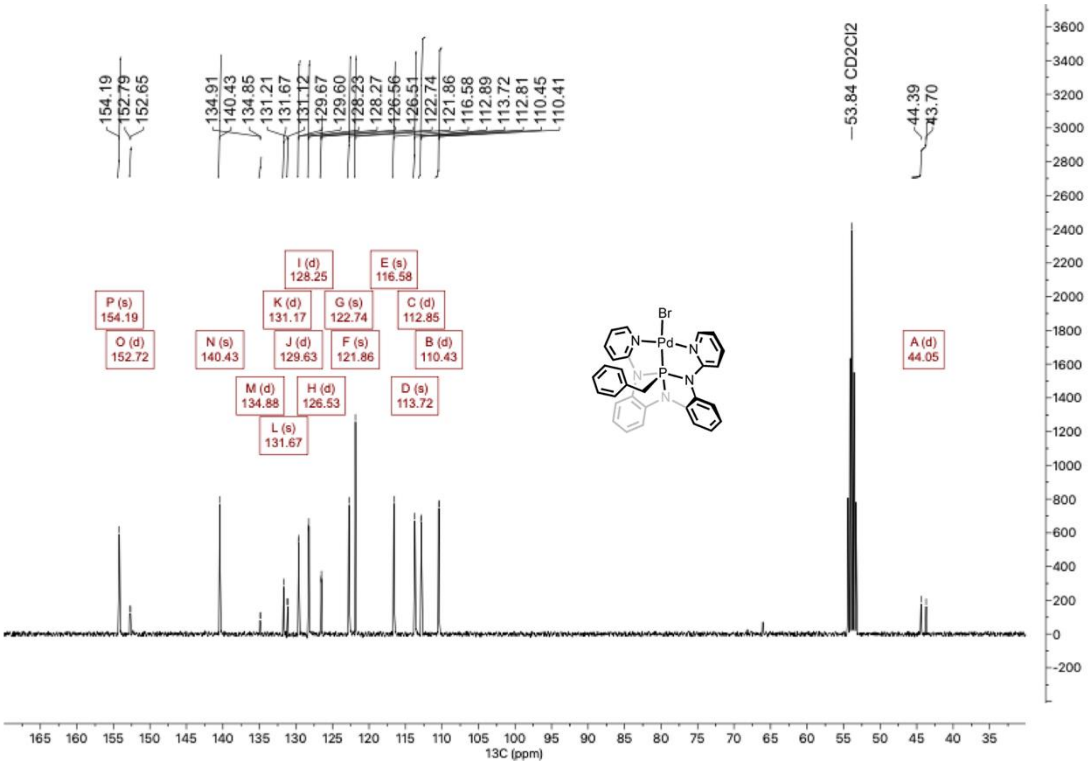

Figure S16. $^{13}C\{^{1}H\}$ NMR spectrum of $\mathbf{L^{Bn}{\bullet}Pd^{Br}}$ (126 MHz, CD_2Cl_2).

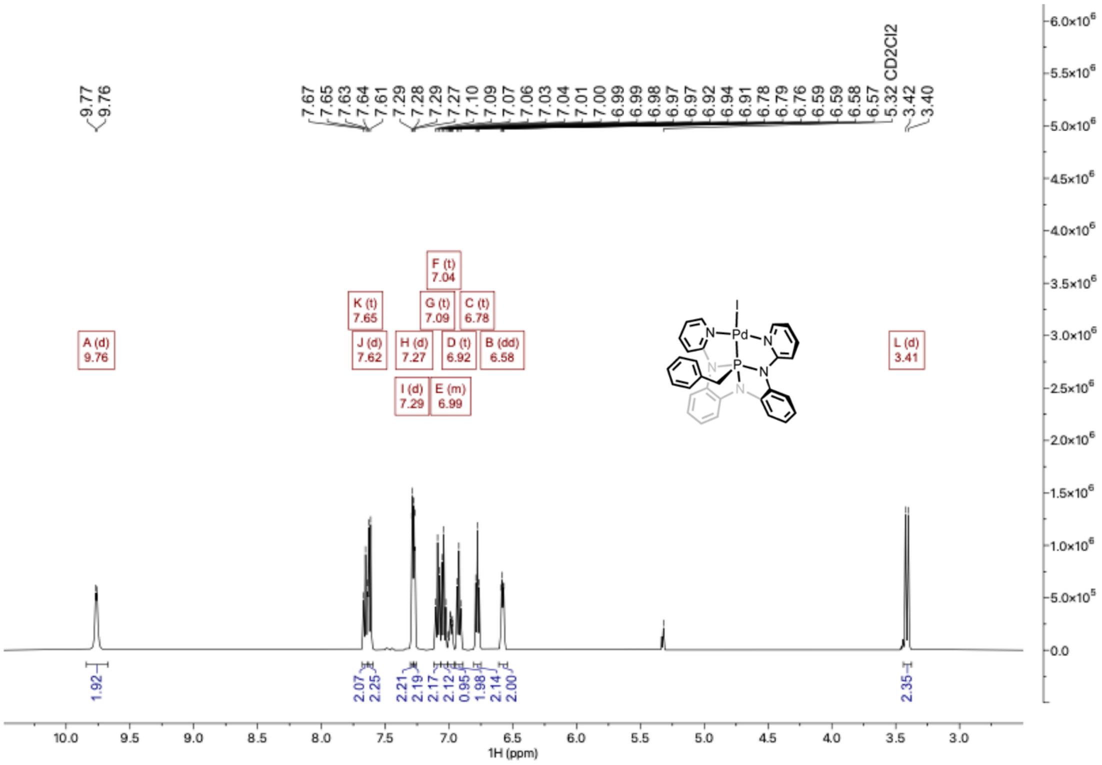

Figure S17. ^{1}H NMR spectrum of $\mathbf{L^{Bn}{\bullet}PdI}$ (400 MHz, CD_2Cl_2).

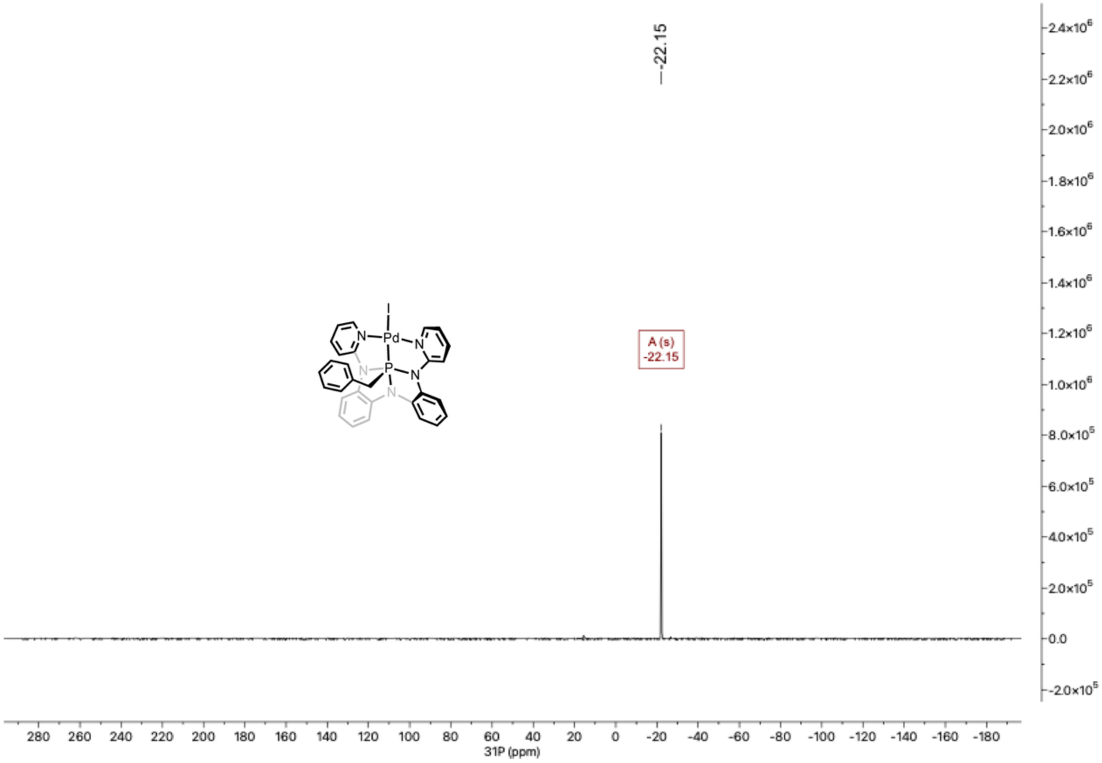

Figure S18. $^{31}P\{^{1}H\}$ NMR spectrum of $\mathbf{L^{Bn}{\bullet}PdI}$ (162 MHz, CD_2Cl_2).

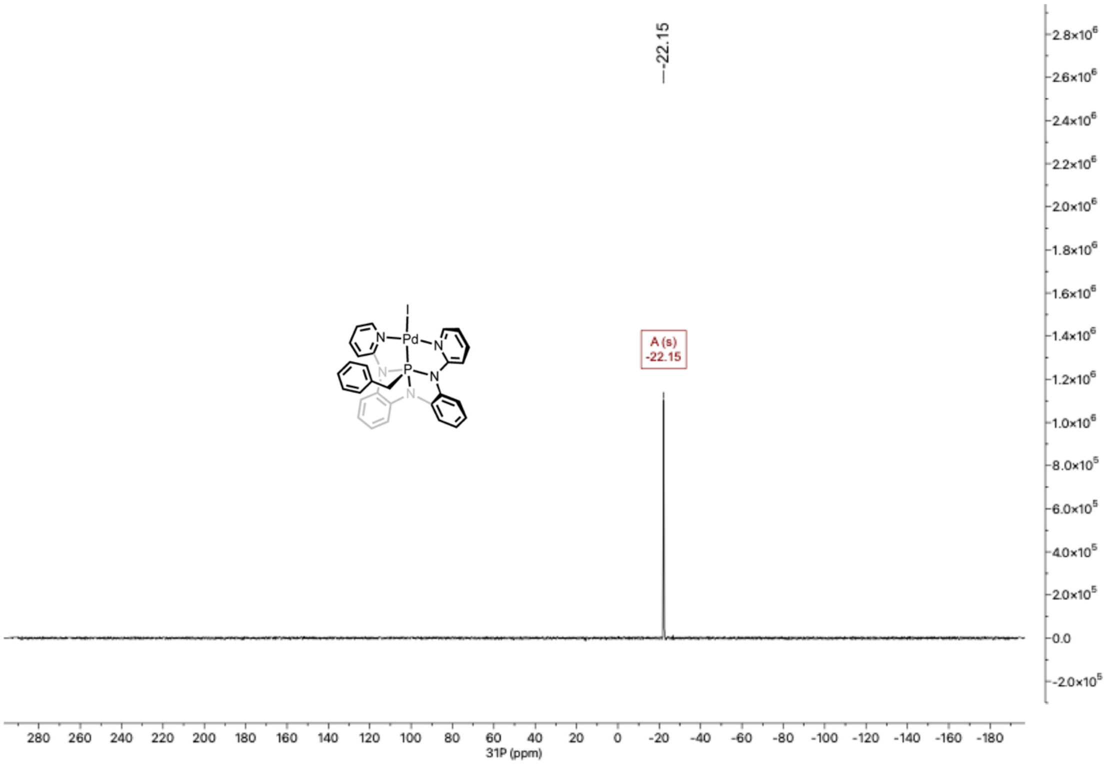

Figure S19. ^{31}P NMR spectrum of $\mathbf{L^{Bn}{\bullet}Pd^{I}}$ (162 MHz, CD_2Cl_2).

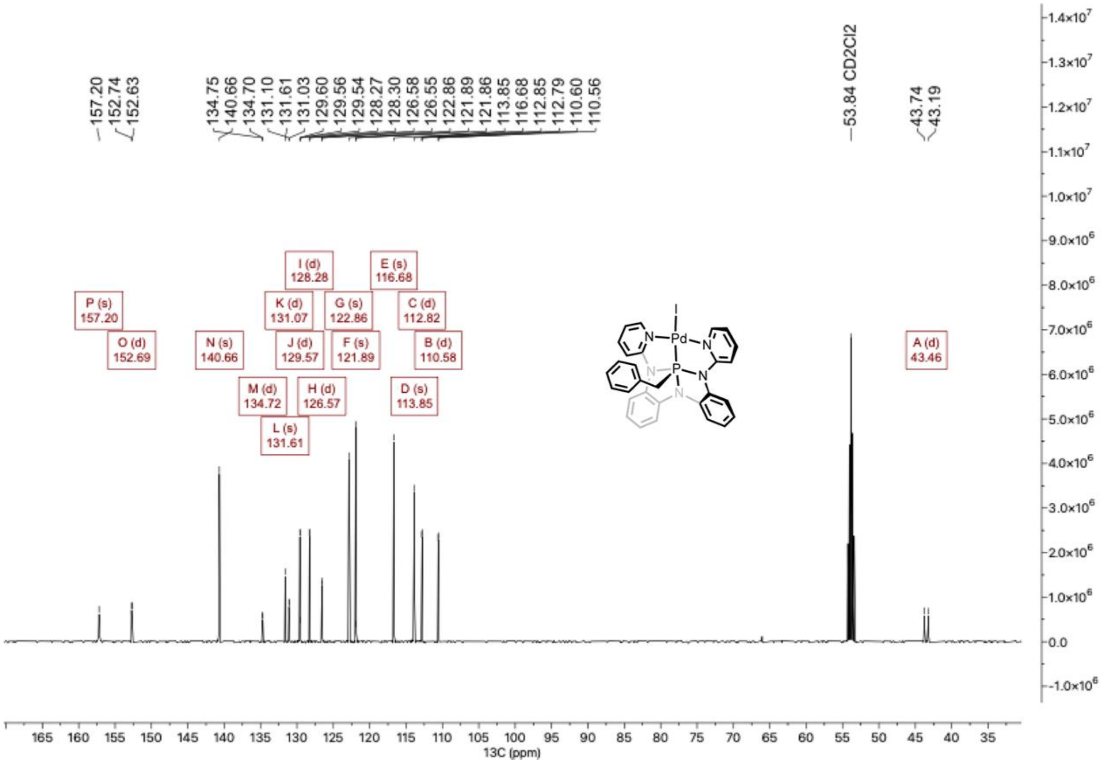

Figure S20. ^{13}C{^{1}H} NMR spectrum of $\mathbf{L^{Bn}{\bullet}Pd^{I}}$ (126 MHz, CD_2Cl_2).

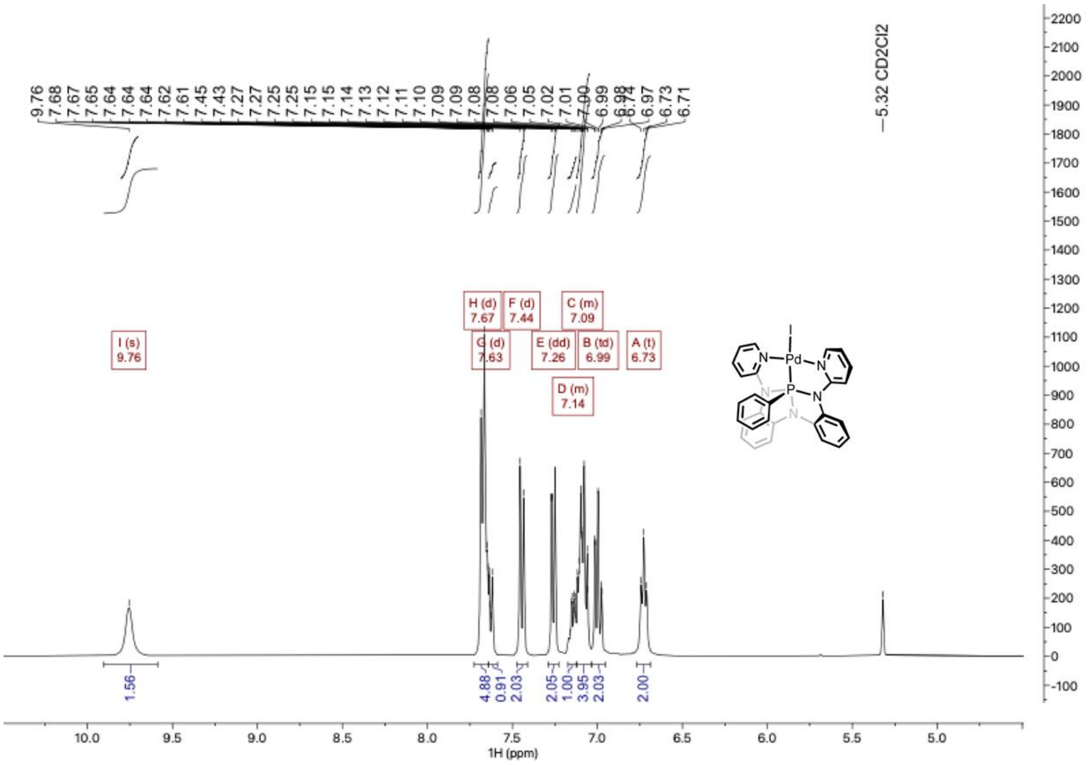

Figure S21. ^{1}H NMR spectrum of $\mathbf{L^{Ph}{\bullet}PdI}$ (400 MHz, CD_2Cl_2).

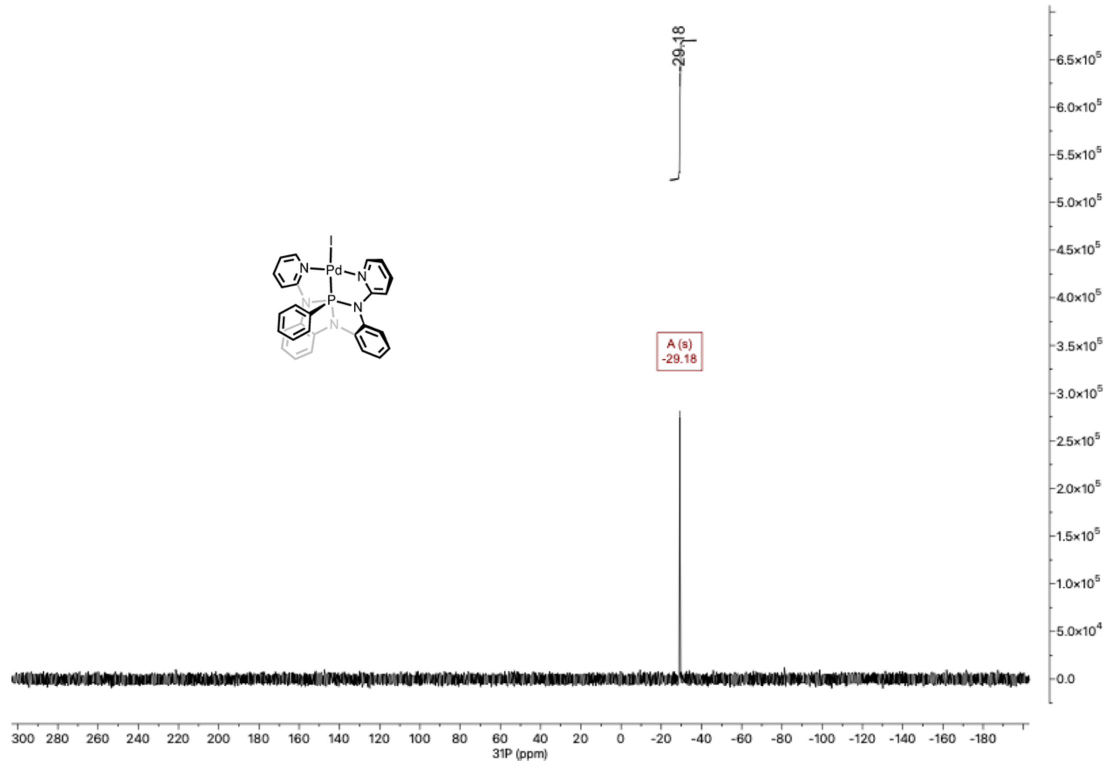

Figure S22. $^{31}P\{^{1}H\}$ NMR spectrum of $\mathbf{L^{Ph}{\bullet}PdI}$ (162 MHz, CD_2Cl_2).

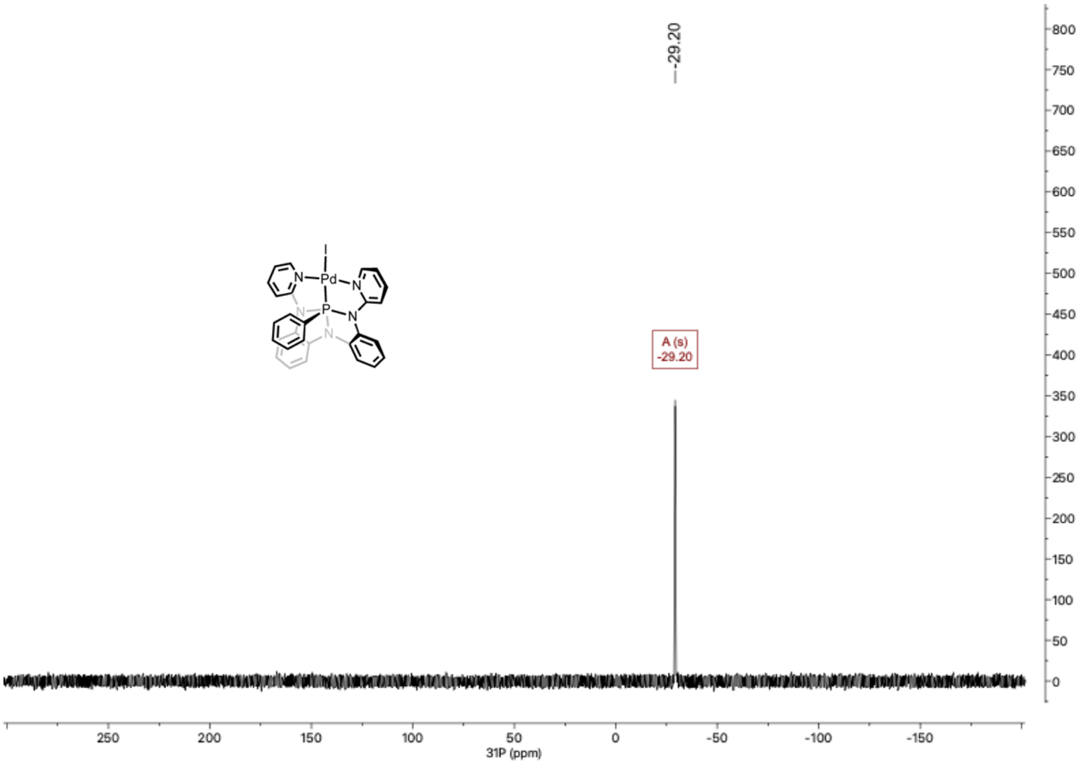

Figure S23. ^{31}P NMR spectrum of $\mathbf{L^{Ph}{\bullet}Pd^{I}}$ (162 MHz, CD_2Cl_2).

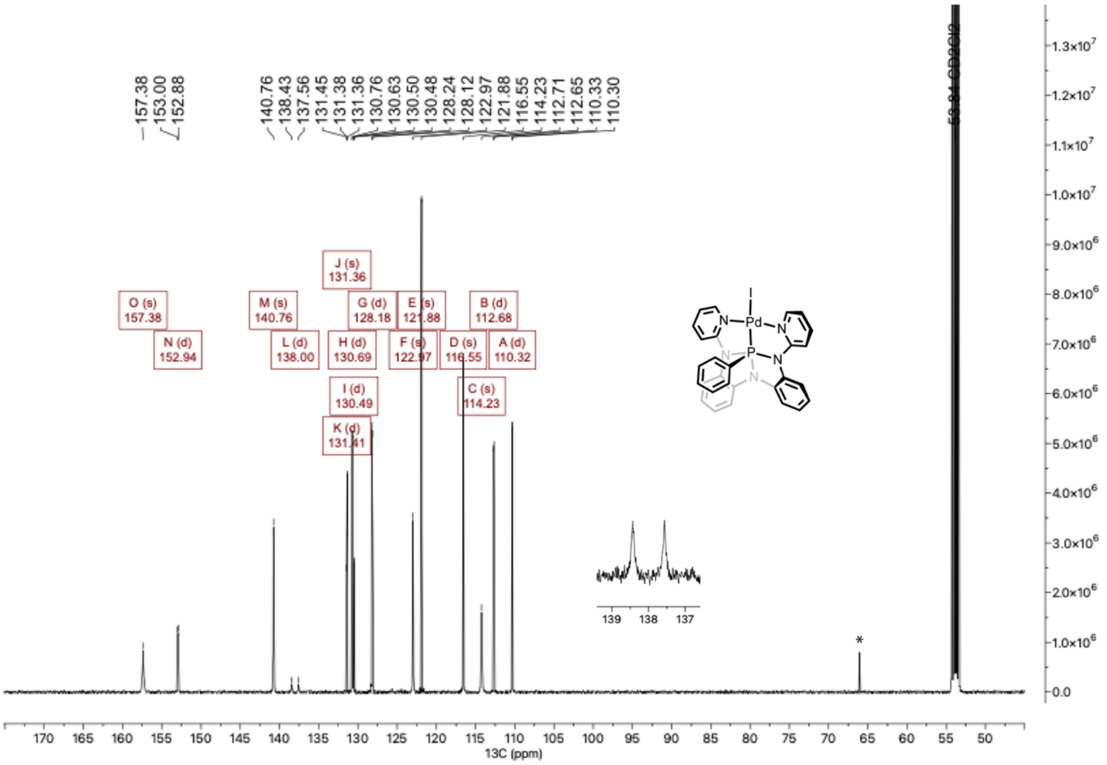

Figure S24. ^{13}C{^{1}H} NMR spectrum of $\mathbf{L^{Ph}{\bullet}Pd^{I}}$ (126 MHz, CD_2Cl_2). *The Et_2O signal at 66.06 ppm is from the solvent co-crystallized in the crystalline sample.

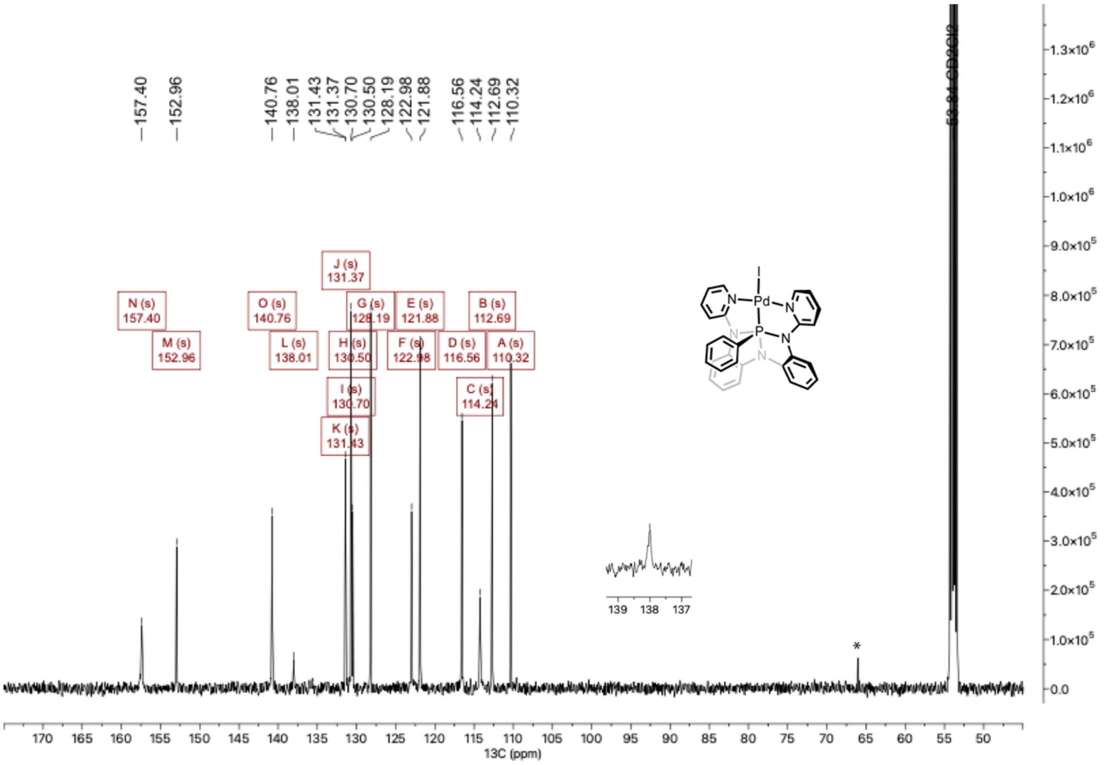

Figure S25. $^{13}C\{^{1}H\}\{^{31}P\}$ NMR spectrum of $\mathbf{L^{Ph}{\bullet}Pd^{I}}$ (126 MHz, CD_2Cl_2). *The Et_2O signal at 66.06 ppm is from the solvent co-crystallized in the crystalline sample.

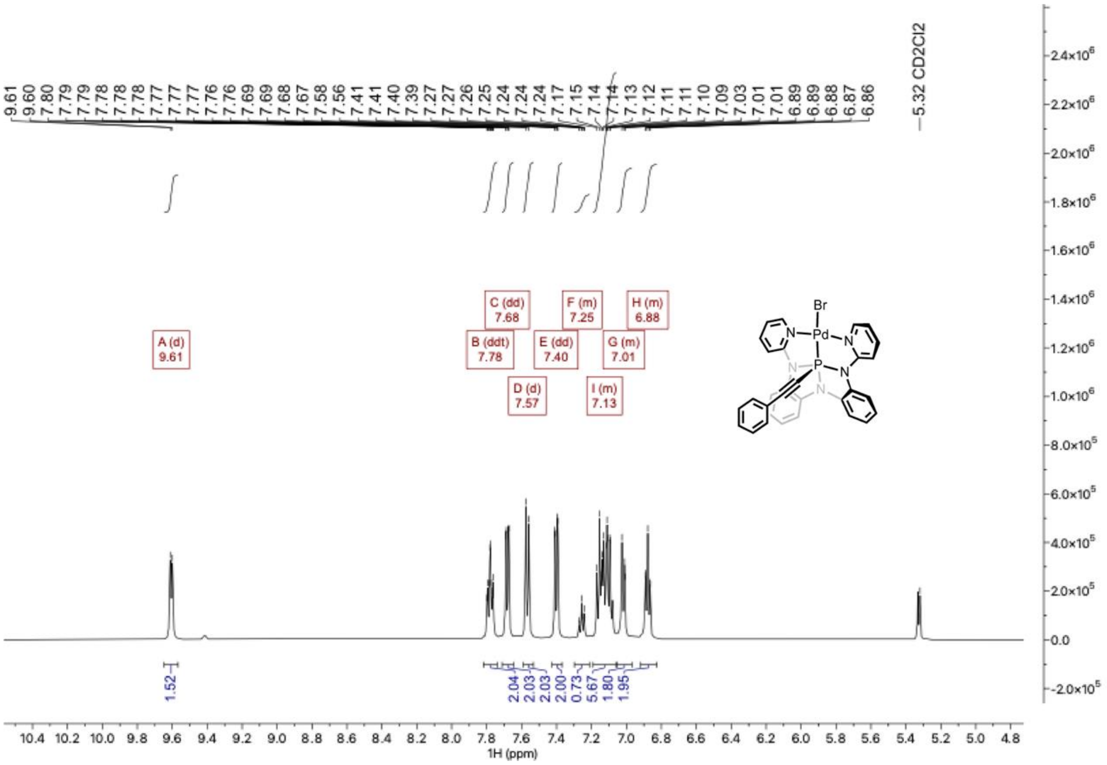

Figure S26. ^{1}H NMR spectrum of $\mathbf{L^{CCPh}{\bullet}Pd^{Br}}$ (400 MHz, CD_2Cl_2).

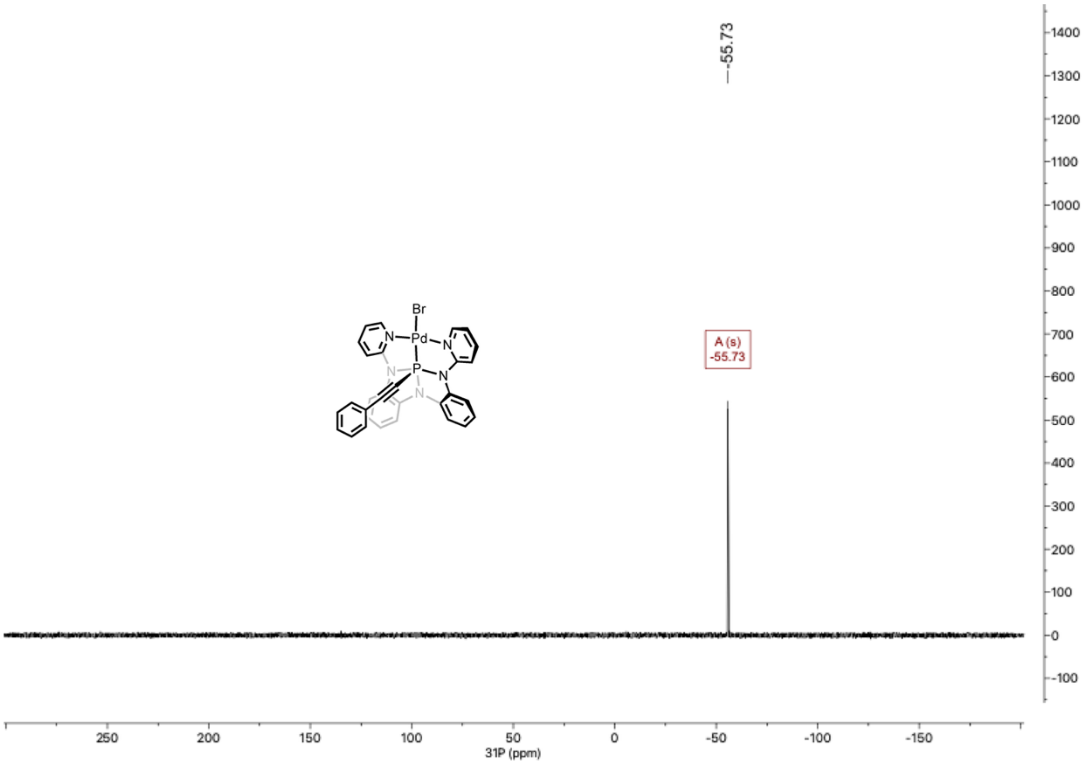

Figure S27. $^{31}P\{^{1}H\}$ NMR spectrum of **L^{CCPh}•Pd^{Br}** (162 MHz, CD_2Cl_2).

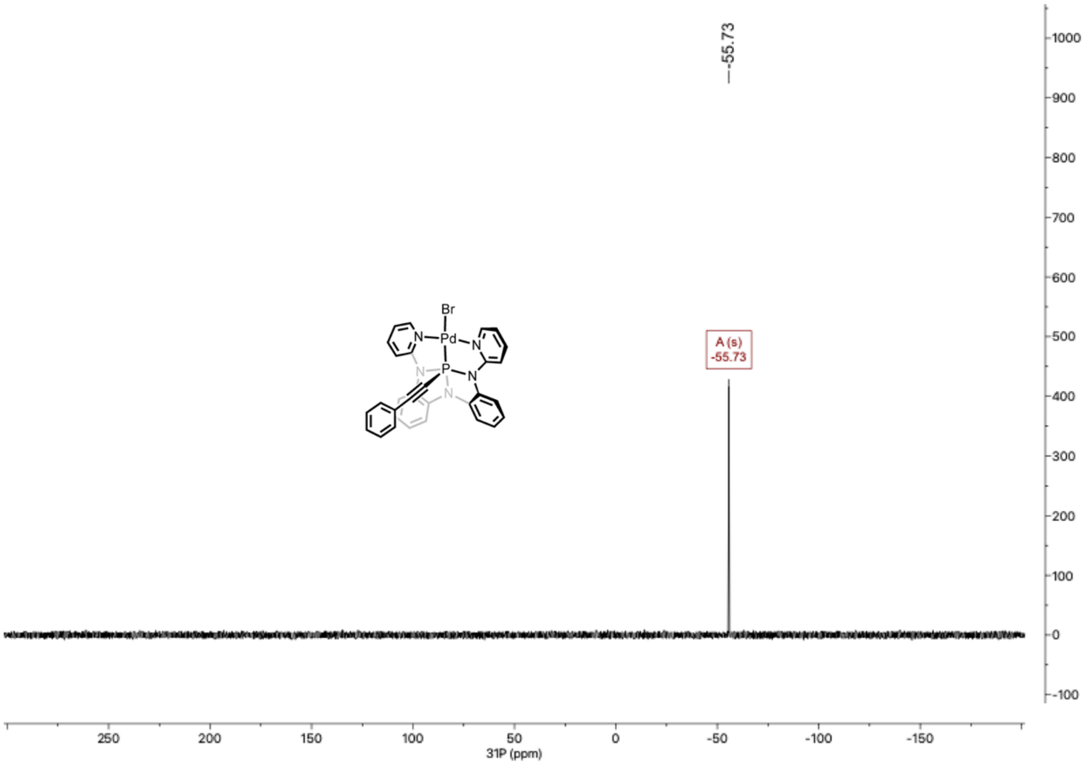

Figure S28. ^{31}P NMR spectrum of **L^{CCPh}•Pd^{Br}** (162 MHz, CD_2Cl_2).

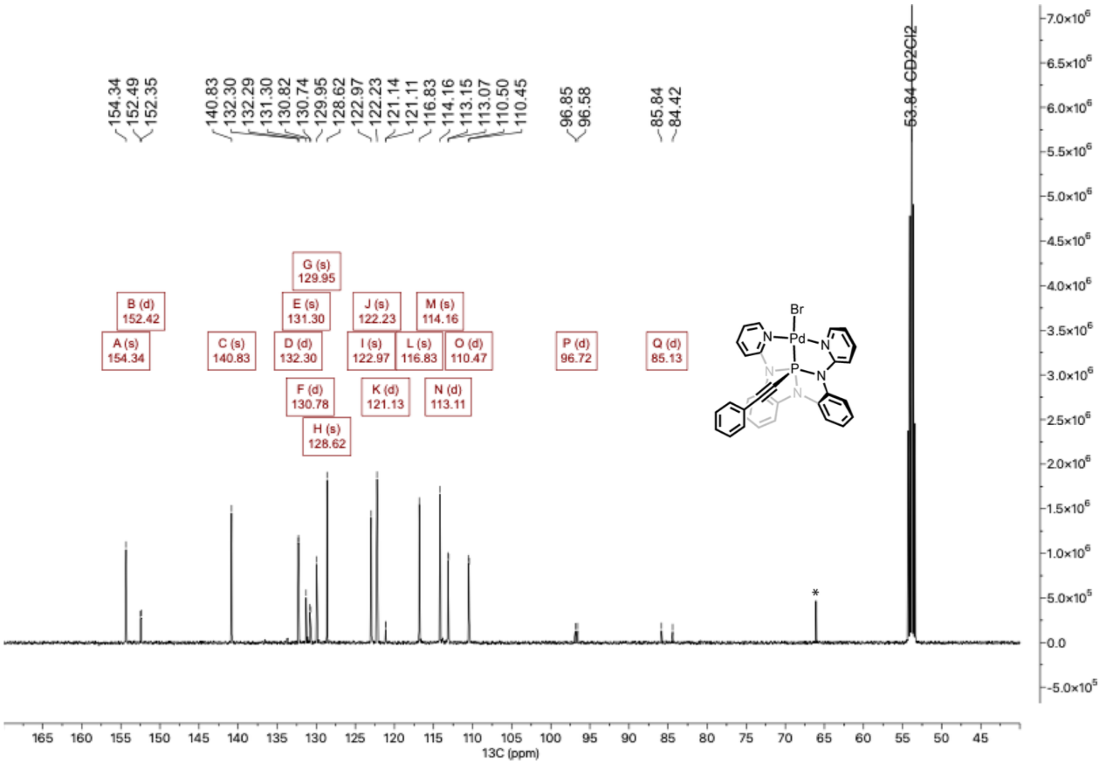

Figure S29. $^{13}C\{^1H\}$ NMR spectrum of $\mathbf{L^{CCPh}{\bullet}Pd^{Br}}$ (126 MHz, CD_2Cl_2). * The Et_2O signal at 66.11 ppm is from the solvent co-crystallized in the crystalline sample.

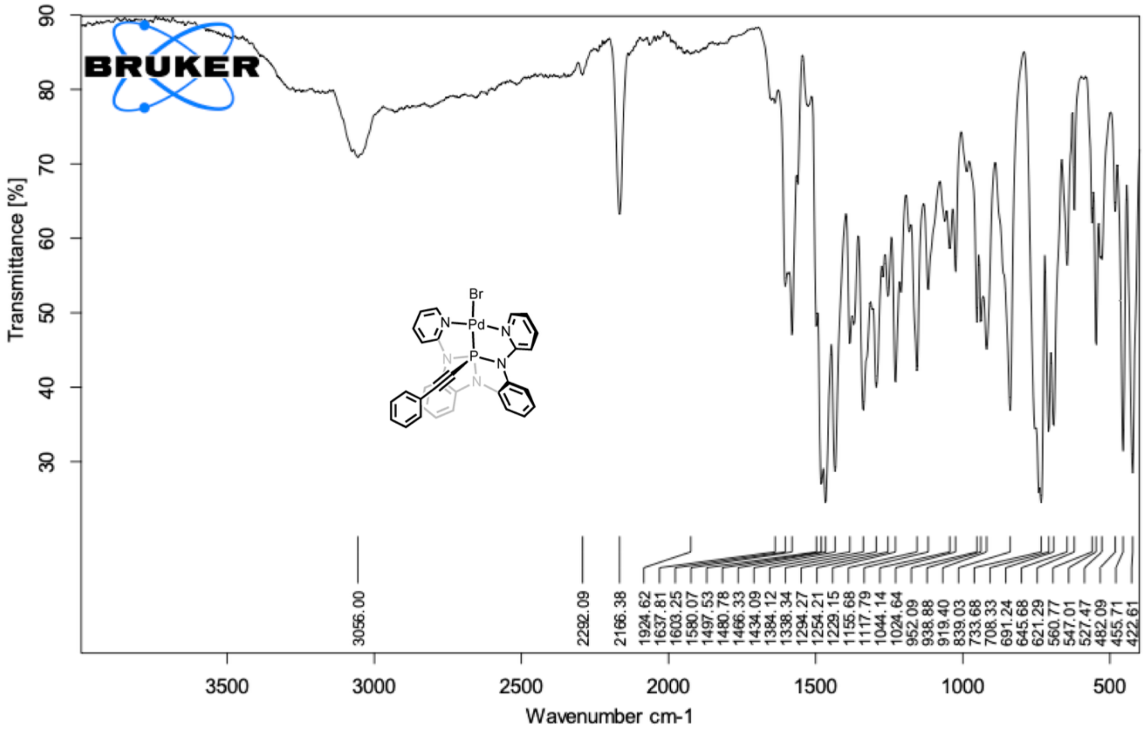

Figure S30. IR spectrum of $\mathbf{L^{CCPh}{\bullet}Pd^{Br}}$.

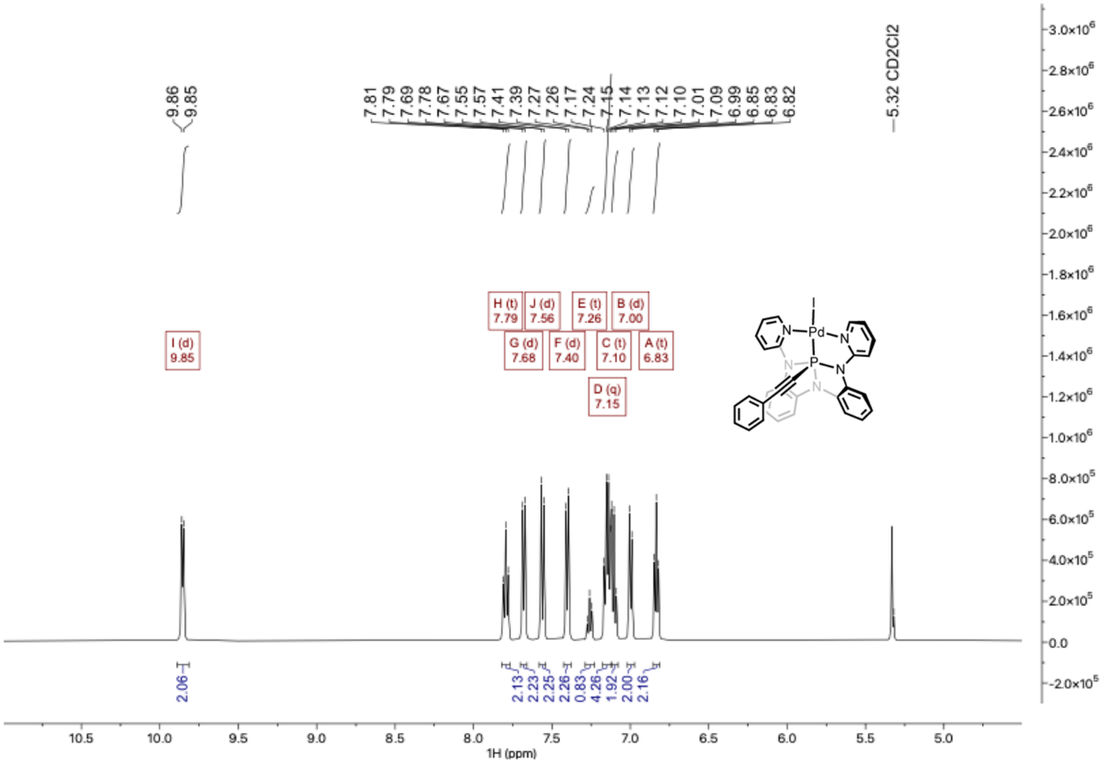

Figure S31. ^{1}H NMR spectrum of $\mathbf{L^{CCPh}{\bullet}Pd^{I}}$ (400 MHz, CD_2Cl_2).

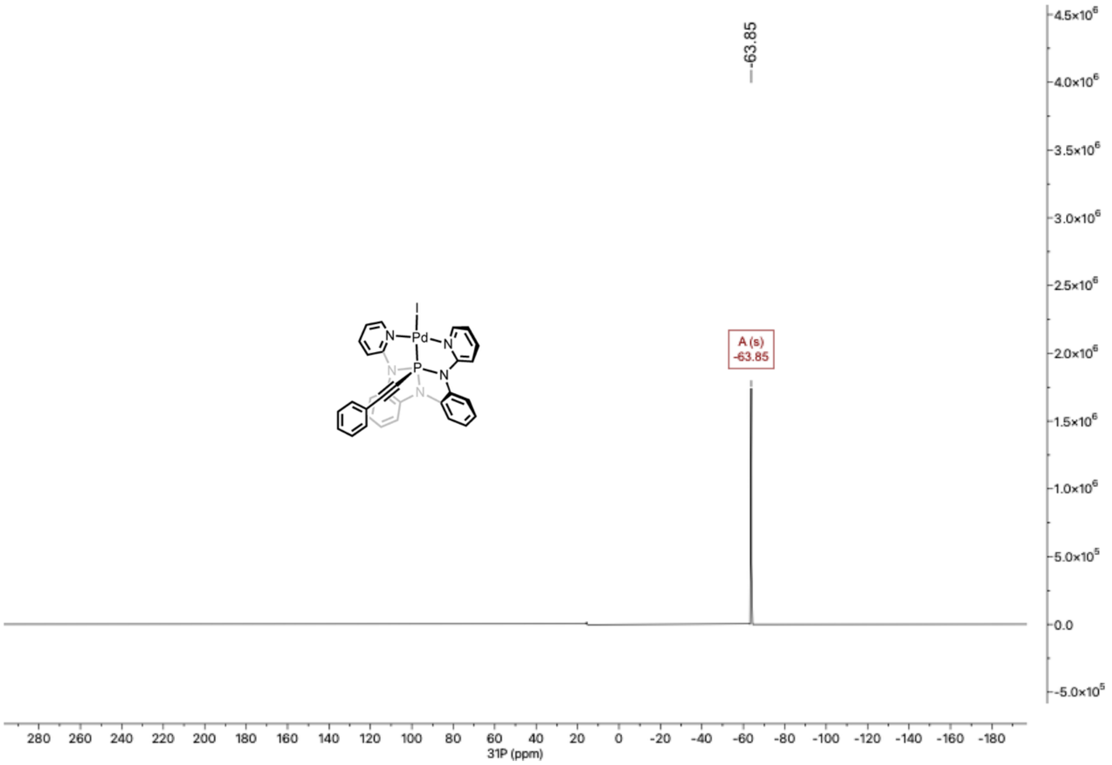

Figure S32. ^{31}P{^{1}H} NMR spectrum of $\mathbf{L^{CCPh}{\bullet}Pd^{I}}$ (162 MHz, CD_2Cl_2).

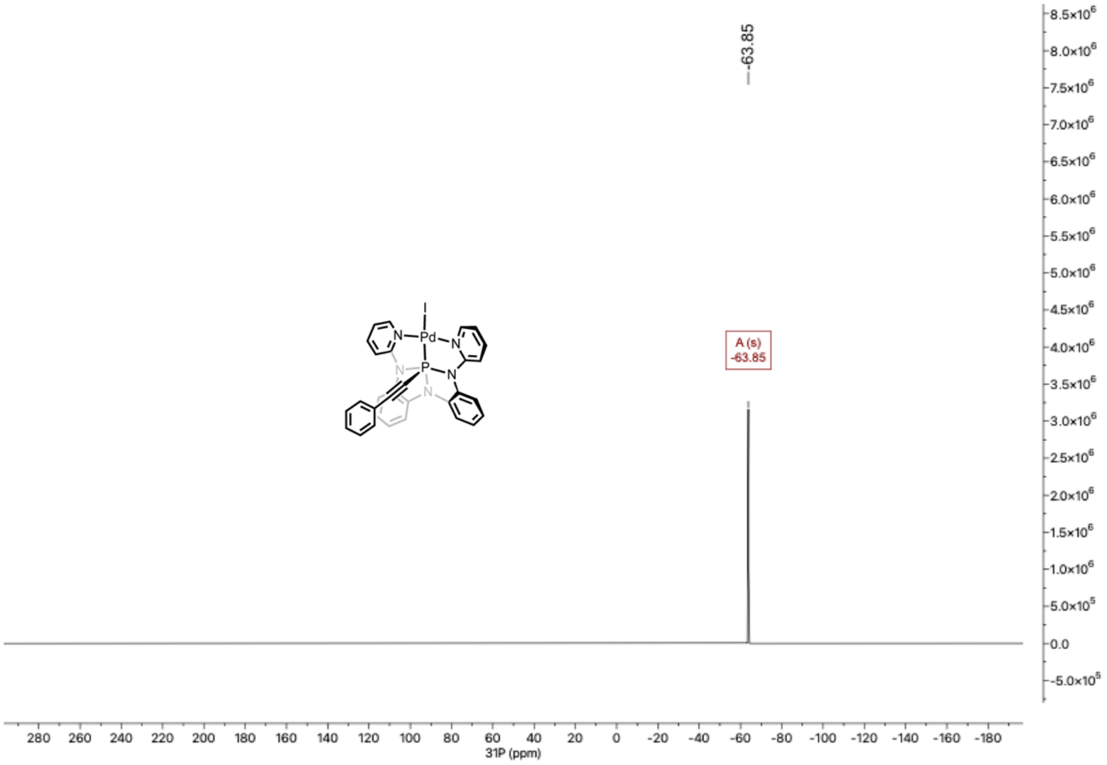

Figure S33. ^{31}P NMR spectrum of $\mathbf{L^{CCPh}{\bullet}Pd^{I}}$ (162 MHz, CD_2Cl_2).

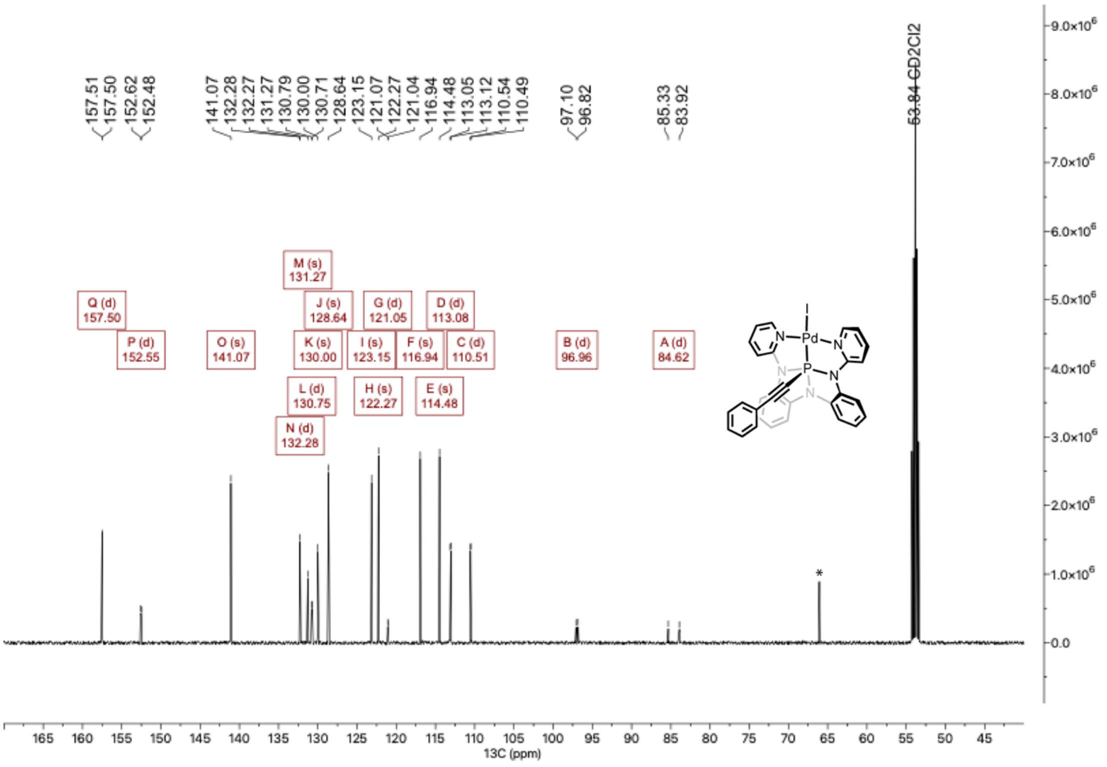

Figure S34. $^{13}C\{^{1}H\}$ NMR spectrum of $\mathbf{L^{CCPh}{\bullet}Pd^{I}}$ (126 MHz, CD_2Cl_2). * The Et_2O signal at 66.11 ppm is from the solvent co-crystallized in the crystalline sample.

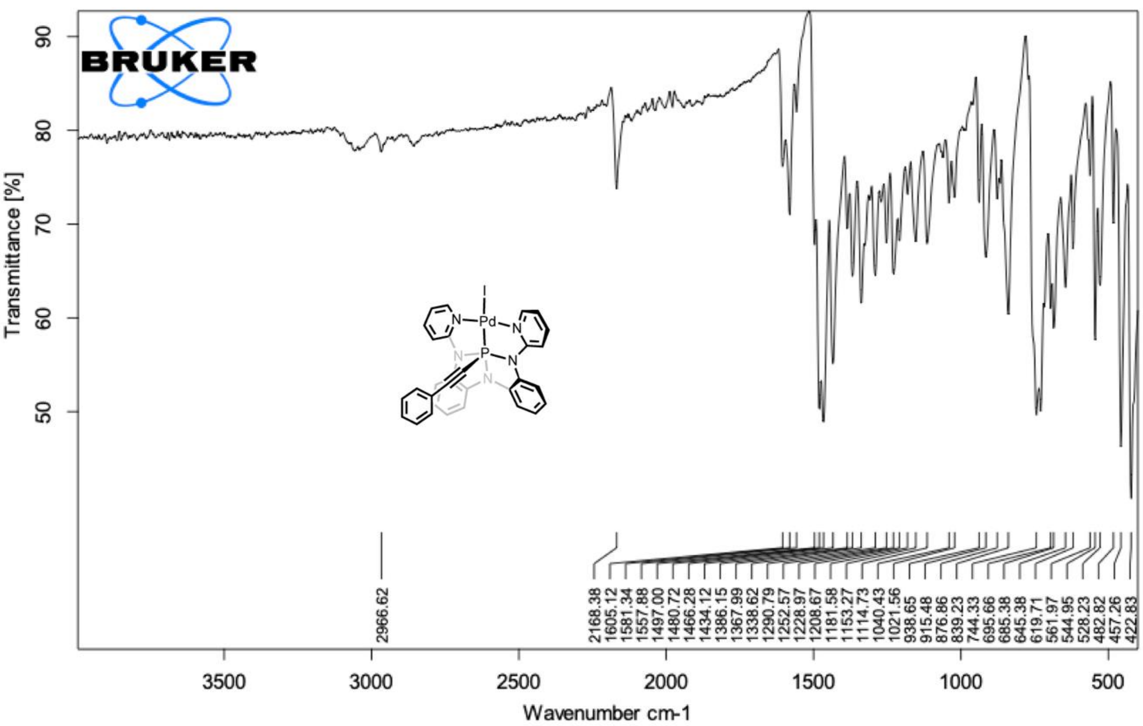

Figure S35. IR spectrum of $\mathbf{L^{CCPh}{\bullet}PdI}$.

IV. Crystallographic Details

All crystal structures reported have been deposited to the Cambridge Crystallographic Data Center (CCDC) and have been assigned the following deposition numbers.

- $\mathbf{L^{Allyl}{\bullet}Pd^{Cl}}$ (G24046): 2371011
- $\mathbf{L^{Allyl}{\bullet}Pd^{Br}}$ (P24167): 2371007
- $\mathbf{L^{Allyl}{\bullet}Pd^{I}}$ (G24199): 2371012
- $\mathbf{L^{Bn}{\bullet}Pd^{Br}}$ (G24048): 2371008
- $\mathbf{L^{Bn}{\bullet}Pd^{I}}$ (G24201): 2371013
- $\mathbf{L^{Ph}{\bullet}Pd^{I}}$ (G24061): 2371015
- $\mathbf{L^{CCPh}{\bullet}Pd^{Br}}$ (G24093_sq): 2371009
- $\mathbf{L^{CCPh}{\bullet}Pd^{I}}$ (P24166): 2371014

Compound $\mathbf{L^{Allyl}{\bullet}Pd^{Cl}}$ crystallizes in the monoclinic centrosymmetric space group $P2_1/c$ with one molecule of $\mathbf{L^{Allyl}{\bullet}Pd^{Cl}}$ per asymmetric unit. No restraints were applied.

Compound $\mathbf{L^{Allyl}{\bullet}Pd^{Br}}$ crystallizes in the monoclinic centrosymmetric space group $P2_1/c$ with one molecule of $\mathbf{L^{Allyl}{\bullet}Pd^{Br}}$ per asymmetric unit. No restraints were applied.

Compound $\mathbf{L^{Allyl}{\bullet}Pd^{I}}$ crystallizes in the monoclinic chiral space group $P2_1$ with one molecule of $\mathbf{L^{Allyl}{\bullet}Pd^{I}}$ per asymmetric unit. The P-bound C_3H_5 moiety was refined as disordered over two positions. The disorder was refined with the help of similarity restraints on 1-2 and 1-3 distances and displacement parameters as well as rigid-bond restraints. The disorder ratio was refined freely and converged at 0.660(11). The absolute structure could be established with high confidence based on resonant scattering; the Flack parameter was calculated by Parson's method[S9] refined to 0.002(2).

Compound $\mathbf{L^{Bn}{\bullet}Pd^{Br}}$ crystallizes in the monoclinic centrosymmetric space group $P2_1/c$ with one molecule of $\mathbf{L^{Bn}{\bullet}Pd^{Br}}$ and half disordered molecule of THF per asymmetric unit. The THF molecule is located near a crystallographic inversion center, which means that the asymmetric unit only contains half a THF molecule. This half molecule was refined as disordered over two positions (making this effectively a four-fold disorder). The disorder was refined with the help of similarity restraints on 1-2 and 1-3 distances and displacement parameters as well as rigid-bond restraints. The disorder ratio was refined freely and converged at 0.850(15).

Compound $\mathbf{L^{Bn}{\bullet}Pd^{I}}$ crystallizes in the orthorhombic space group $P2_12_12_1$ with one molecule of $\mathbf{L^{Bn}{\bullet}Pd^{I}}$ per asymmetric unit. The absolute structure could be established with high confidence based on resonant scattering; the Flack parameter as calculated by Parson's method[S9] refined to -0.017(3). No restraints were applied.

Compound **L^{Ph}•Pd^{I}** crystallizes in the triclinic centrosymmetric space group *P*-1 with one half molecule of **L^{Ph}•Pd^{I}** and one molecule of THF per asymmetric unit. The other half of the target molecule is generated by the crystallographic inversion center. No restraints were applied.

Compound **L^{CCPh}•Pd^{Br}** crystallizes in the orthorhombic centrosymmetric space group *Pbcn* with one molecule of **L^{CCPh}•Pd^{Br}** per asymmetric unit. In addition, there is a highly disordered solvent present, and the parameterization of the solvent model was not stable. The program Squeeze[S10] was implemented in Platon[S11] allows to account for the contribution of disordered solvent contained in voids within the crystal lattice. The solvent contribution is added to the model in a separate file (the .fab file) by SHELXL. This procedure was applied to the structure of **L^{CCPh}•Pd^{Br}**. Squeeze identified one crystallographically independent solvent-accessible void with a volume of 337 Å^3, located at coordinates 0.00, 0.23, 0.25. This is enough space for *ca*. 15 non-hydrogen atoms or 3 molecules of THF or pentane. In this void, Squeeze identified the equivalent of 96 electrons, corresponding to about two to three THF or pentane molecules.

Compound **L^{CCPh}•Pd^{I}** crystallizes in the monoclinic centrosymmetric space group $P2_1/c$ with two molecules of **L^{CCPh}•Pd^{I}** and three molecules of THF per asymmetric unit. In one of the independent molecules, a ligand-pyridine moiety was refined as disordered over two positions. The disorder ratio was refined freely and converged at 0.78(2). In addition, the structure was found to be twinned by pseudo-merohedry. The twin law is 1 0 0 0 -1 0 0 0 -1 with the twin ratio refined to 0.4578(6). Similarity restraints on 1-2 and 1-3 distances were applied wherever applicable, and similar ADP and rigid-bond restraints were applied to all atoms to stabilize the refinement and to counteract correlation effects introduced by the twinning.[S12]

Figure S36. Solid-state structure of $\mathbf{L^{Allyl}{\bullet}Pd^{Cl}}$ (G24046). Thermal ellipsoids are rendered at 50% probability level. All hydrogen atoms are omitted for clarity.

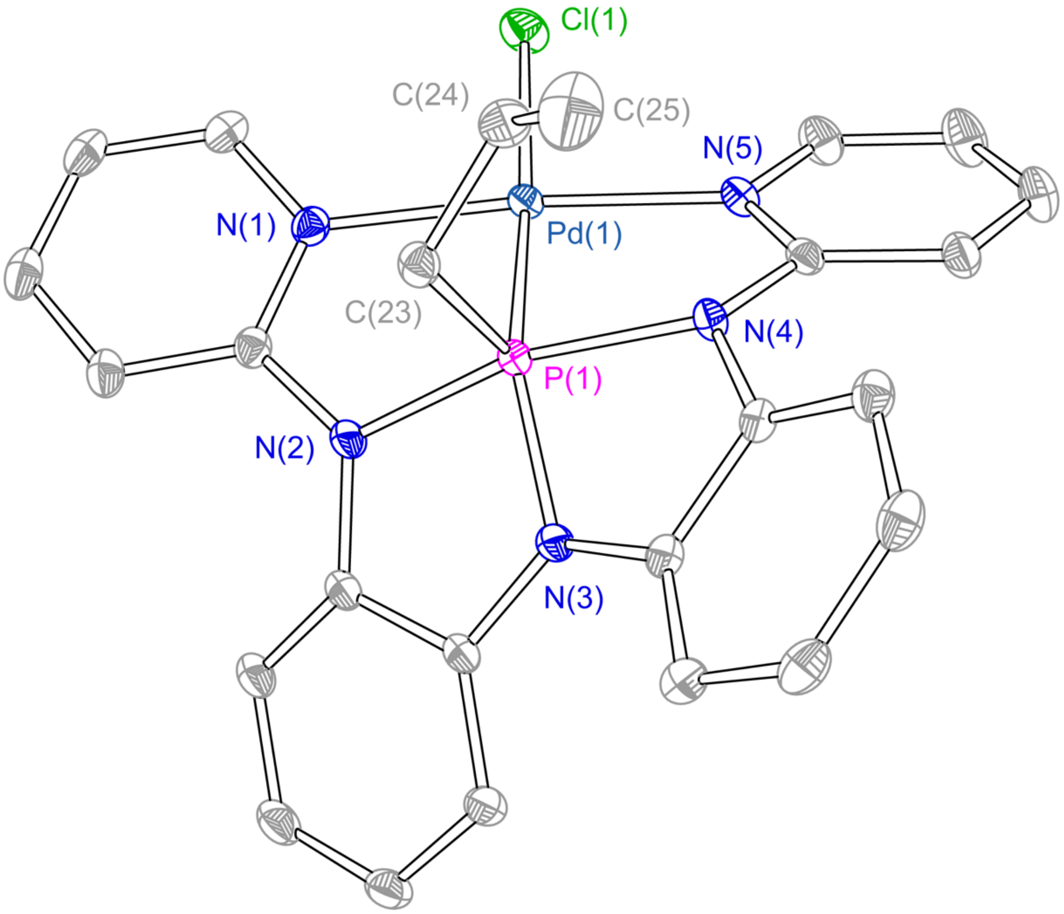

Table S1. Crystal data and structure refinement for **L^{Allyl}•Pd^{Cl}** (G24046)

Identification code	G24046	
Empirical formula	$C_{25} H_{21} Cl N_5 P Pd$	
Formula weight	564.29	
Temperature	100(2) K	
Wavelength	0.71073 Å	
Crystal system	Monoclinic	
Space group	$P2_1/c$	
Unit cell dimensions	a = 10.0315(4) Å	α= 90°
	b = 13.8677(6) Å	β= 96.6667(16)°
	c = 15.9418(7) Å	γ= 90°
Volume	2202.73(16) Å^3	
Z	4	
Density (calculated)	1.702 Mg/ m^3	
Absorption coefficient	1.062 mm^{-1}	
F(000)	1136	
Crystal size	0.345 x 0.340 x 0.310 mm^3	
Theta range for data collection	1.952 to 31.546°	
Index ranges	-14<=h<=14, -20<=k<=20, -23<=l<=23	
Reflections collected	145538	
Independent reflections	7353 [R_{int} = 0.0348]	
Completeness to theta = 25.242°	100.0 %	
Absorption correction	Semi-empirical from equivalents	
Max. and min. transmission	0.6049 and 0.5260	
Refinement method	Full-matrix least-squares on F^2	
Data / restraints / parameters	7353 / 0 / 298	
Goodness-of-fit on F^2	1.056	
Final R indices [I>2σ(I)]	R1 = 0.0191, wR2 = 0.0466	
R indices (all data)	R1 = 0.0213, wR2 = 0.0475	
Largest diff. peak and hole	0.495 and -0.594 e.Å^{-3}	

Figure S37. Solid-state structure of $\mathbf{L^{Allyl}{\bullet}Pd^{Br}}$ (P24167). Thermal ellipsoids are rendered at 50% probability level. All hydrogen atoms are omitted for clarity.

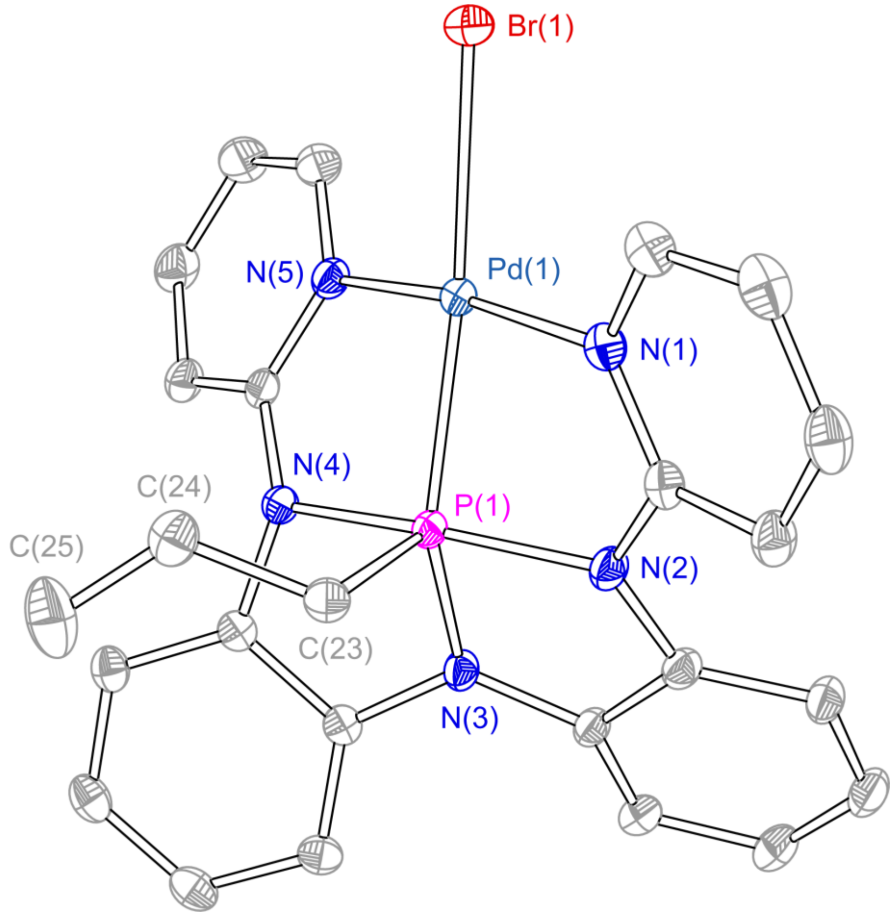

Table S2. Crystal data and structure refinement for $\mathbf{L^{Allyl}}$•$\mathbf{Pd^{Br}}$, P24167

Identification code	P24167	
Empirical formula	$C_{25} H_{21} Br N_5 P Pd$	
Formula weight	608.75	
Temperature	100(2) K	
Wavelength	0.71073 Å	
Crystal system	Monoclinic	
Space group	$P2_1/c$	
Unit cell dimensions	a = 10.1121(5) Å	α= 90°
	b = 13.9083(7) Å	β= 96.5476(17)°
	c = 16.0223(7) Å	γ= 90°
Volume	2238.71(19) $Å^3$	
Z	4	
Density (calculated)	1.806 Mg/m^3	
Absorption coefficient	2.710 mm^{-1}	
F(000)	1208	
Crystal size	0.405 x 0.305 x 0.105 mm^3	
Theta range for data collection	1.944 to 31.576°	
Index ranges	-14<=h<=14, -20<=k<=20, -22<=l<=23	
Reflections collected	149993	
Independent reflections	7481 [R_{int} = 0.0483]	
Completeness to theta = 25.242°	100.0 %	
Absorption correction	Semi-empirical from equivalents	
Refinement method	Full-matrix least-squares on F^2	
Data / restraints / parameters	7481 / 0 / 298	
Goodness-of-fit on F^2	1.028	
Final R indices [I>2σ(I)]	R1 = 0.0219, $wR2$ = 0.0501	
R indices (all data)	R1 = 0.0269, $wR2$ = 0.0521	
Largest diff. peak and hole	0.829 and -0.680 e.$Å^{-3}$	

Figure S38. Solid-state structure of **L**[Allyl]**•Pd**[I] (G24199). Thermal ellipsoids are rendered at 50% probability level. All hydrogen atoms are omitted for clarity.

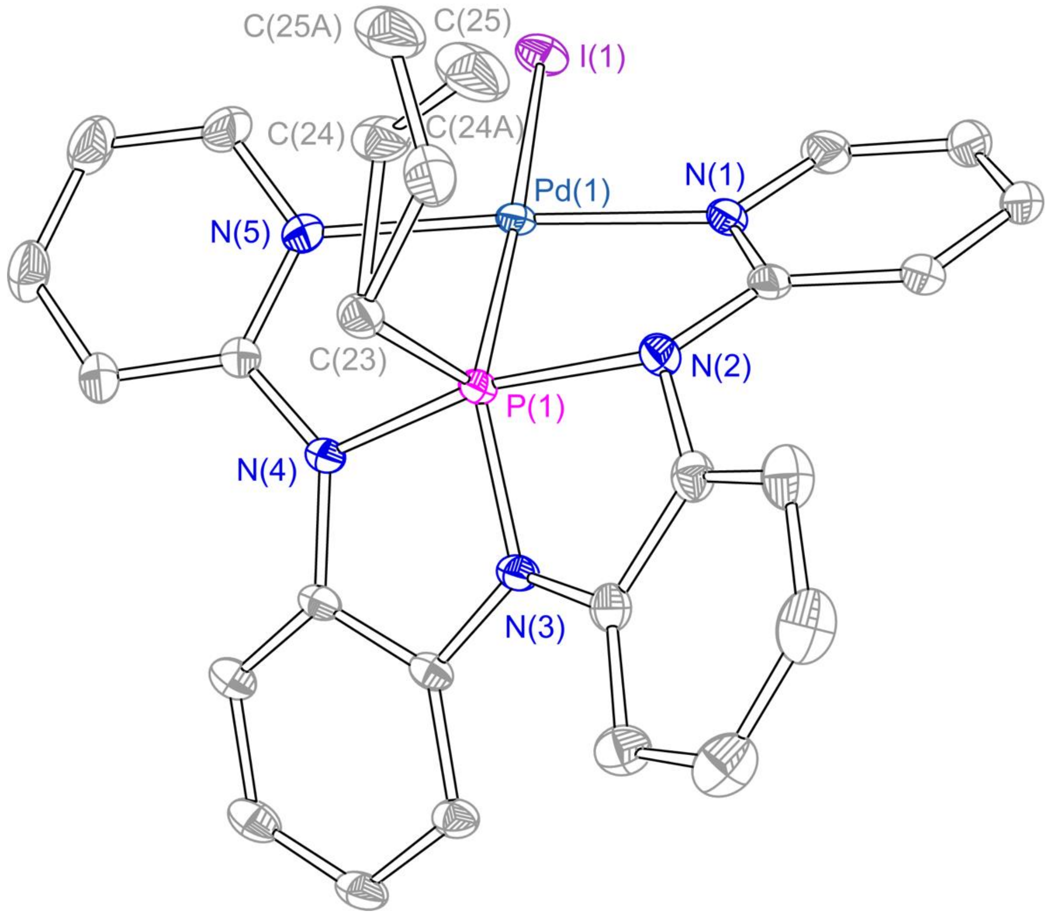

Table S3. Crystal data and structure refinement for **L^{Allyl}•Pd^{I}**, G24199

Identification code	G24199	
Empirical formula	$C_{25} H_{21} I N_5 P Pd$	
Formula weight	655.74	
Temperature	100(2) K	
Wavelength	0.71073 Å	
Crystal system	Monoclinic	
Space group	$P2_1$	
Unit cell dimensions	a = 9.5083(4) Å	α= 90°
	b = 12.3089(5) Å	β= 104.4556(14)°
	c = 10.2965(4) Å	γ= 90°
Volume	1166.92(8) $Å^3$	
Z	2	
Density (calculated)	1.866 Mg/m^3	
Absorption coefficient	2.211 mm^{-1}	
F(000)	640	
Crystal size	0.355 x 0.330 x 0.260 mm^3	
Theta range for data collection	2.043 to 31.532°	
Index ranges	$-13<=h<=13$, $-18<=k<=18$, $-15<=l<=15$	
Reflections collected	78581	
Independent reflections	7753 [R_{int} = 0.0297]	
Completeness to theta = 25.242°	99.9 %	
Absorption correction	Semi-empirical from equivalents	
Refinement method	Full-matrix least-squares on F^2	
Data / restraints / parameters	7753 / 83 / 317	
Goodness-of-fit on F^2	1.062	
Final R indices [I>2σ(I)]	$R1$ = 0.0163, $wR2$ = 0.0364	
R indices (all data)	$R1$ = 0.0173, $wR2$ = 0.0367	
Absolute structure parameter	0.002(3)	
Largest diff. peak and hole	1.200 and -0.637 e.$Å^{-3}$	

Figure S39. Solid-state structure of $\mathbf{L^{Bn}}$•$\mathbf{Pd^{Br}}$ (G24048). Thermal ellipsoids are rendered at 50% probability level. All hydrogen atoms and solvent molecules are omitted for clarity.

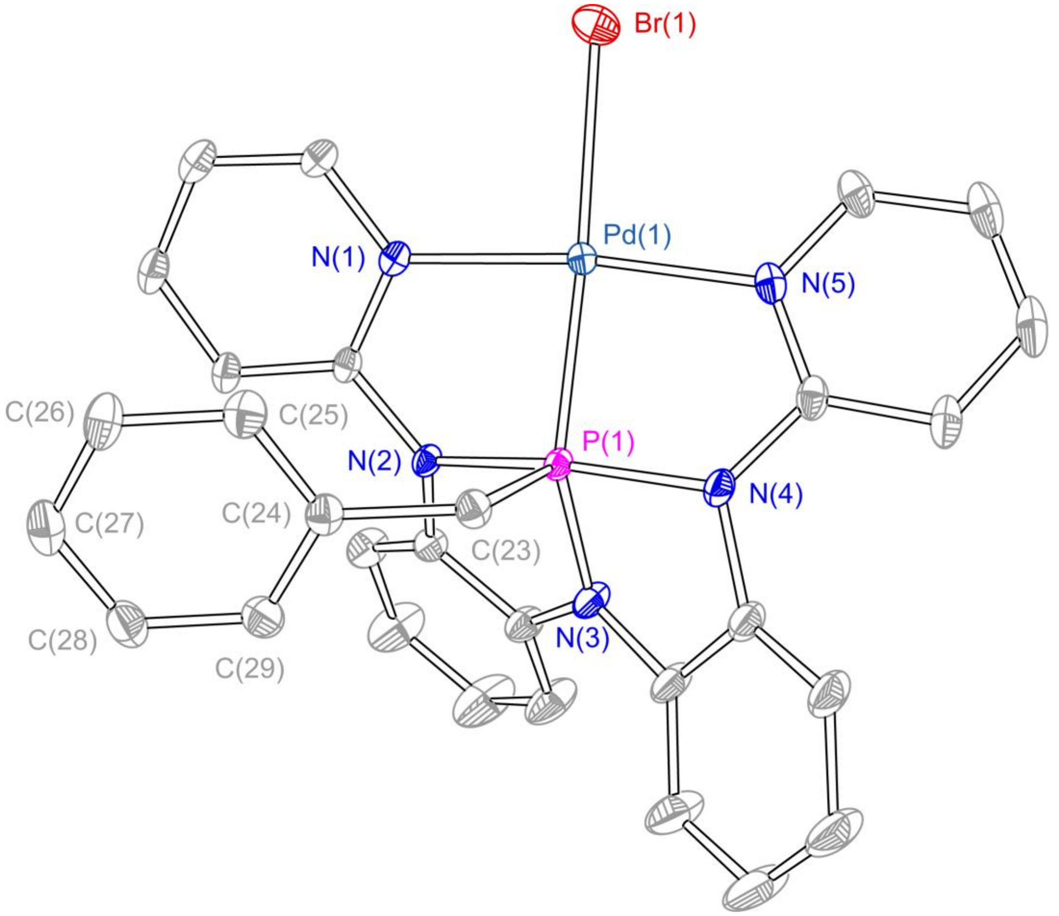

Table S4. Crystal data and structure refinement for $\mathbf{L^{Bn}}$•$\mathbf{Pd^{Br}}$, G24048

Identification code	G24048	
Empirical formula	$C_{31} H_{27} Br N_5 O_{0.50} P Pd$	
Formula weight	694.85	
Temperature	100(2) K	
Wavelength	0.71073 Å	
Crystal system	Monoclinic	
Space group	$P2_1/c$	
Unit cell dimensions	a = 19.4392(13) Å	α= 90°
	b = 15.8886(9) Å	β= 102.302(3)°
	c = 8.9501(6) Å	γ= 90°
Volume	2700.9(3) $Å^3$	
Z	4	
Density (calculated)	1.709 Mg/m^3	
Absorption coefficient	2.260 mm^{-1}	
F(000)	1392	
Crystal size	0.200 x 0.130 x 0.030 mm^3	
Theta range for data collection	2.145 to 31.523°	
Index ranges	$-28<=h<=28, -23<=k<=21, -13<=l<=13$	
Reflections collected	160665	
Independent reflections	8990 [R_{int} = 0.0529]	
Completeness to theta = 25.242°	99.9 %	
Absorption correction	Semi-empirical from equivalents	
Refinement method	Full-matrix least-squares on F^2	
Data / restraints / parameters	8990 / 236 / 425	
Goodness-of-fit on F^2	1.055	
Final R indices [I>2σ(I)]	R1 = 0.0317, $wR2$ = 0.0902	
R indices (all data)	R1 = 0.0346, $wR2$ = 0.0925	
Largest diff. peak and hole	1.113 and -2.260 e.$Å^{-3}$	

Figure S40. Solid-state structure of $\mathbf{L^{Bn}{\bullet}Pd^{I}}$ (G24201). Thermal ellipsoids are rendered at 50% probability level. All hydrogen atoms are omitted for clarity.

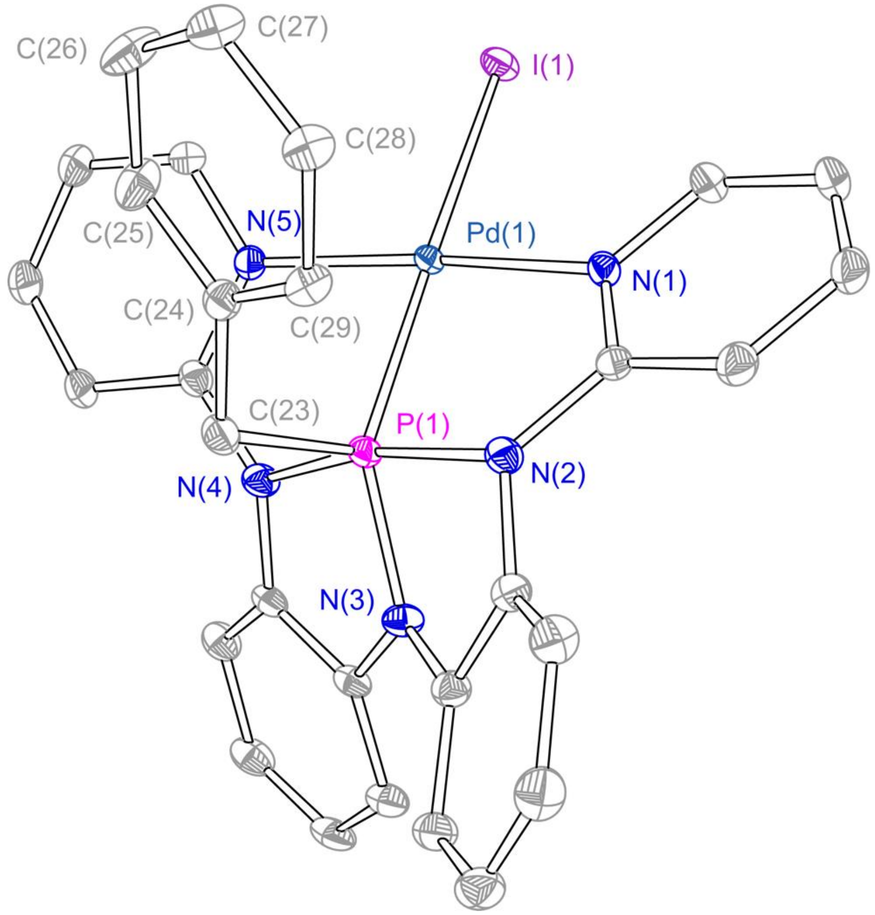

Table S5. Crystal data and structure refinement for $\mathbf{L^{Bn}}$**•**$\mathbf{Pd^{I}}$, G24201

Identification code	G24201	
Empirical formula	C_{29} H_{23} I N_5 P Pd	
Formula weight	705.79	
Temperature	100(2) K	
Wavelength	0.71073 Å	
Crystal system	Orthorhombic	
Space group	$P2_12_12_1$	
Unit cell dimensions	*a* = 9.1625(3) Å	α= 90°
	b = 13.6231(3) Å	β= 90°
	c = 20.1844(6) Å	γ= 90°
Volume	2519.45(12) $Å^3$	
Z	4	
Density (calculated)	1.861 Mg/m^3	
Absorption coefficient	2.055 mm^{-1}	
F(000)	1384	
Crystal size	0.430 x 0.400 x 0.125 mm^3	
Theta range for data collection	1.803 to 31.522°	
Index ranges	-13<=*h*<=13, -20<=*k*<=17, -29<=*l*<=29	
Reflections collected	119944	
Independent reflections	8404 [R_{int} = 0.0340]	
Completeness to theta = 25.242°	99.9 %	
Absorption correction	Semi-empirical from equivalents	
Refinement method	Full-matrix least-squares on F^2	
Data / restraints / parameters	8404 / 0 / 334	
Goodness-of-fit on F^2	1.100	
Final R indices [I>2σ(I)]	R1 = 0.0151, *wR*2 = 0.0349	
R indices (all data)	R1 = 0.0165, *wR*2 = 0.0352	
Absolute structure parameter	-0.017(3)	
Largest diff. peak and hole	0.355 and -0.518 e.$Å^{-3}$	

Figure S41. Solid-state structure of $\mathbf{L^{Ph}{\bullet}Pd^{I}}$ (G24061). Thermal ellipsoids are rendered at 50% probability level. All hydrogen atoms and solvent molecules are omitted for clarity.

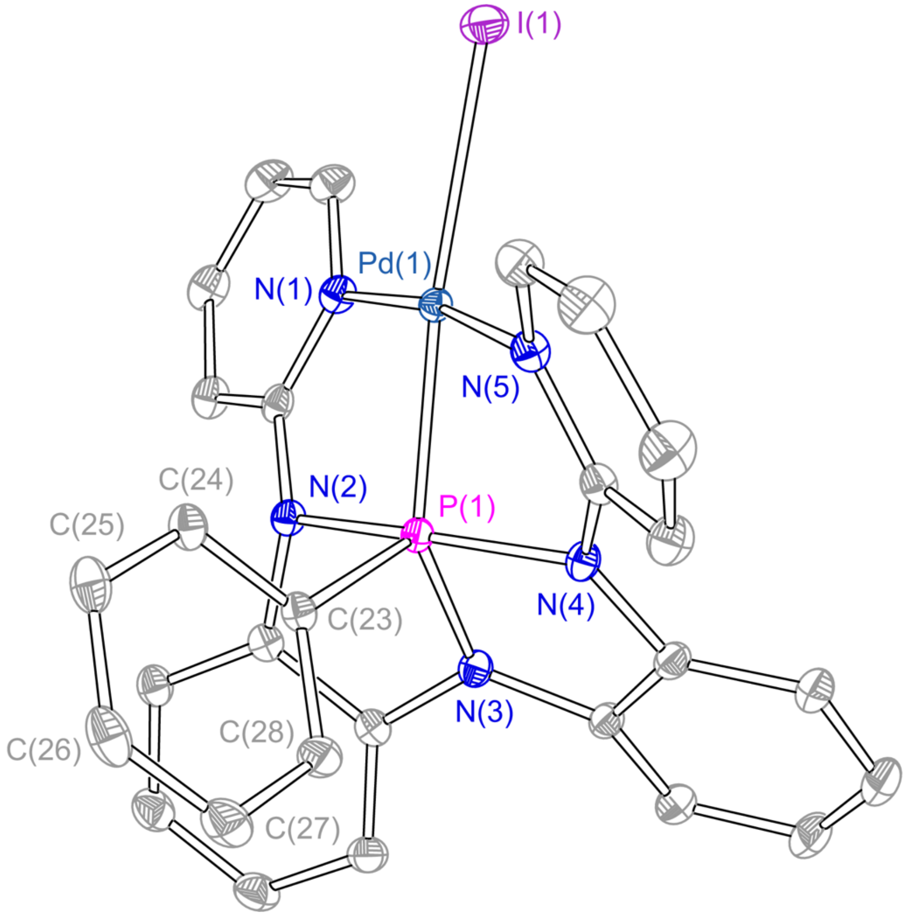

Table S6. Crystal data and structure refinement for $\mathbf{L^{Ph}{\bullet}Pd^{I}}$, G24061

Identification code	G24061	
Empirical formula	$C_{32} H_{29} I N_5 O P Pd$	
Formula weight	763.87	
Temperature	100(2) K	
Wavelength	0.71073 Å	
Crystal system	Triclinic	
Space group	*P*-1	
Unit cell dimensions	a = 8.4456(7) Å	α= 109.838(3)°
	b = 12.2746(11) Å	β= 100.056(3)°
	c = 15.8098(14) Å	γ= 101.651(3)°
Volume	1456.4(2) $Å^3$	
Z	2	
Density (calculated)	1.742 Mg/m^3	
Absorption coefficient	1.788 mm^{-1}	
F(000)	756	
Crystal size	0.225 x 0.155 x 0.070 mm^3	
Theta range for data collection	1.420 to 31.575°	
Index ranges	-12<=*h*<=12, -18<=*k*<=18, -23<=*l*<=23	
Reflections collected	112292	
Independent reflections	9749 [R_{int} = 0.0338]	
Completeness to theta = 25.242°	99.9 %	
Absorption correction	Semi-empirical from equivalents	
Refinement method	Full-matrix least-squares on F^2	
Data / restraints / parameters	9749 / 4 / 370	
Goodness-of-fit on F^2	1.069	
Final R indices [I>2σ(I)]	R1 = 0.0229, *wR*2 = 0.0566	
R indices (all data)	R1 = 0.0259, *wR*2 = 0.0580	
Largest diff. peak and hole	0.783 and -1.315 e.$Å^{-3}$	

Figure S42. Solid-state structure of $\mathbf{L^{CCPh}{\bullet}Pd^{Br}}$ (G24093_sq). Thermal ellipsoids are rendered at 50% probability level. All hydrogen atoms are omitted for clarity.

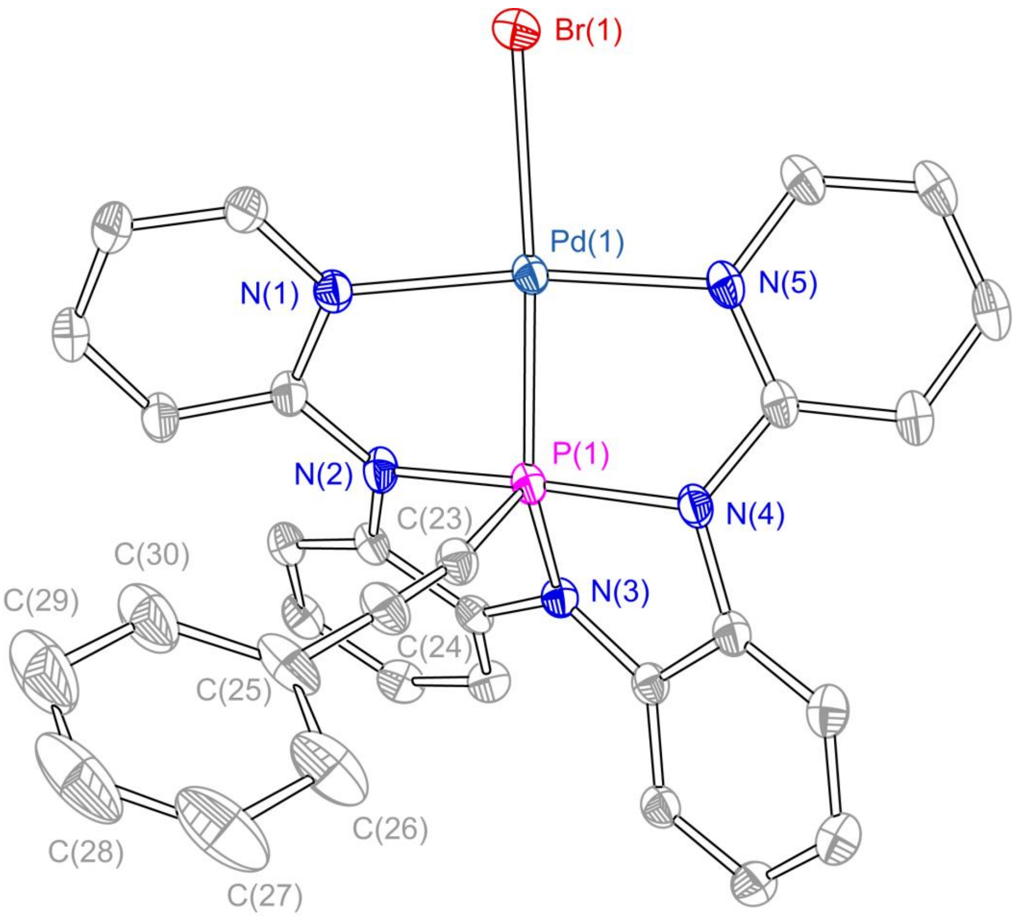

Table S7. Crystal data and structure refinement for $\mathbf{L^{CCPh}{\bullet}Pd^{Br}}$, G24093_sq

Identification code	G24093_sq	
Empirical formula	C_{30} H_{21} Br N_5 P Pd	
Formula weight	668.80	
Temperature	100(2) K	
Wavelength	0.71073 Å	
Crystal system	Orthorhombic	
Space group	*Pbcn*	
Unit cell dimensions	a = 15.6946(8) Å	α= 90°
	b = 21.5183(12) Å	β= 90°
	c = 17.9958(10) Å	γ= 90°
Volume	6077.6(6) Å^3	
Z	8	
Density (calculated)	1.462 Mg/m^3	
Absorption coefficient	2.004 mm^{-1}	
F(000)	2656	
Crystal size	0.285 x 0.250 x 0.030 mm^3	
Theta range for data collection	1.965 to 33.180°	
Index ranges	-24<=*h*<=21, -33<=*k*<=33, -27<=*l*<=27	
Reflections collected	335390	
Independent reflections	11621 [R_{int} = 0.0703]	
Completeness to theta = 25.242°	100.0 %	
Absorption correction	Semi-empirical from equivalents	
Refinement method	Full-matrix least-squares on F^2	
Data / restraints / parameters	11621 / 0 / 343	
Goodness-of-fit on F^2	1.040	
Final R indices [I>2σ(I)]	R1 = 0.0335, *wR*2 = 0.0814	
R indices (all data)	R1 = 0.0464, *wR*2 = 0.0877	
Largest diff. peak and hole	1.084 and -1.246 e.Å^{-3}	

Figure S43. Solid-state structure of $\mathbf{L^{CCPh}{\bullet}Pd^{I}}$ (P24166). Thermal ellipsoids are rendered at 50% probability level. All hydrogen atoms and solvent molecules are omitted for clarity.

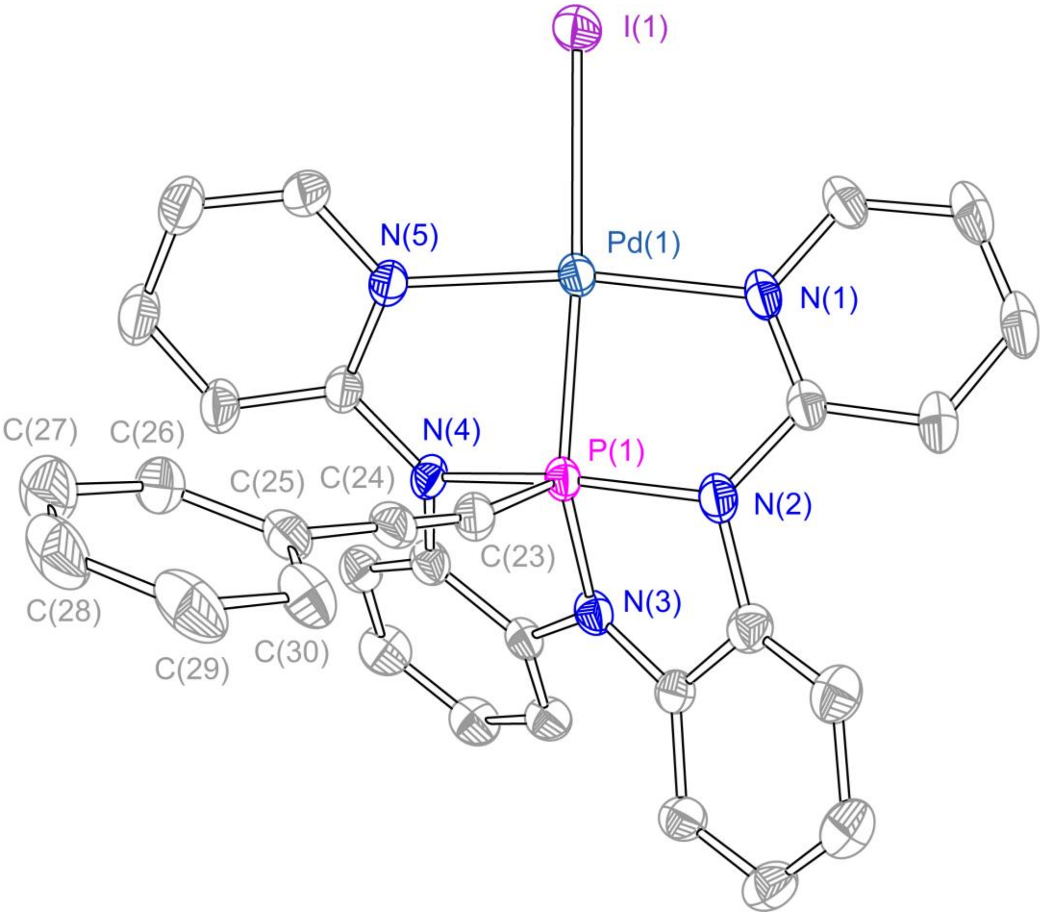

Table S8. Crystal data and structure refinement for $\mathbf{L^{CCPh} \bullet Pd^{I}}$, P24166

Identification code	P24166	
Empirical formula	$C_{36} H_{33} I N_5 O_{1.50} P Pd$	
Formula weight	823.94	
Temperature	100(2) K	
Wavelength	0.71073 Å	
Crystal system	Monoclinic	
Space group	$P2_1/c$	
Unit cell dimensions	a = 18.0435(14) Å	α= 90°
	b = 15.3267(13) Å	β= 90.013(3)°
	c = 23.646(2) Å	γ= 90°
Volume	6539.2(9) $Å^3$	
Z	8	
Density (calculated)	1.674 Mg/m^3	
Absorption coefficient	1.600 mm^{-1}	
F(000)	3280	
Crystal size	0.205 x 0.160 x 0.065 mm^3	
Theta range for data collection	1.329 to 31.590°.	
Index ranges	$-26<=h<=26$, $-22<=k<=22$, $-34<=l<=34$	
Reflections collected	555606	
Independent reflections	21885 [R_{int} = 0.0729]	
Completeness to theta = 25.242°	100.0 %	
Absorption correction	Semi-empirical from equivalents	
Refinement method	Full-matrix least-squares on F^2	
Data / restraints / parameters	21885 / 1686 / 858	
Goodness-of-fit on F^2	1.062	
Final R indices [I>2σ(I)]	R1 = 0.0420, $wR2$ = 0.0968	
R indices (all data)	R1 = 0.0537, $wR2$ = 0.1044	
Largest diff. peak and hole	1.649 and -2.006 e.$Å^{-3}$	

V. Computational Details for Natural Localized Molecular Orbital Analysis

Molecular geometries of $\mathbf{L^R{\bullet}Pd^X}$ reported in this communication were optimized in Orca 5.0.3 using PBE0 density functional with def2-TZVP basis set.[S13–S15] Palladium and iodine atoms were represented with zeroth-order regular approximation (ZORA) to account for the relativistic effect.[S16] The calculation was carried out in the gas phase without symmetry constraint, and reproduced the experimental structures with good agreement. Stationary points were characterized by frequency calculations to confirm their identity as local minima (zero imaginary frequencies). Natural localized molecular orbital (NLMO) analysis was performed at PBE0/def2-TZVP theory level using NBO 7.0 with the optimized structures.[S17] ZORA was similarly applied to the palladium and iodine atoms. The visualization and analysis of the intrinsic bond orbitals (IBOs) were performed using IBOview.[S18]

a) Comparison of Crystallographic and Computed Structures

Bond distances (Å) and bond angles (deg)

Table S9. Compound **L^{Allyl}•Pd^{X}** (X = Cl, Br, I)

	**L^{Allyl}•Pd^{Cl}**		**L^{Allyl}•Pd^{Br}**		**L^{Allyl}•Pd^{I}**	
	Expt.	Calc.	Expt.	Calc.	Expt.	Calc.
Pd(1)-X(1)	2.4144(3)	2.38233	2.5493(2)	2.53283	2.7546(2)	2.72486
Pd(1)-P(1)	2.2218(3)	2.22563	2.2306(4)	2.23071	2.2311(6)	2.23748
Pd(1)-N(1)	2.0311(10)	2.0297	2.0665(13)	2.03686	2.050(2)	2.04384
Pd(1)-N(5)	2.0565(10)	2.05488	2.0377(13)	2.06124	2.067(2)	2.0723
P(1)-N(3)	1.7447(10)	1.74916	1.7475(13)	1.74867	1.7328(19)	1.74864
P(1)-N(2)	1.8404(10)	1.84547	1.8325(12)	1.8403	1.839(2)	1.8372
P(1)-N(4)	1.8355(10)	1.86145	1.8431(12)	1.85931	1.857(2)	1.85607
P(1)-C(23)	1.8440(12)	1.85201	1.8435(15)	1.85134	1.846(2)	1.85082
N(5)-Pd(1)-N(1)	173.03(4)	173.579	172.33(5)	172.504	171.09(9)	171.3572
N(5)-Pd(1)-P(1)	84.91(3)	85.593	88.16(4)	87.7008	84.08(6)	84.1981
N(1)-Pd(1)-P(1)	88.23(3)	87.986	84.60(4)	85.0631	87.62(6)	87.1778
N(5)-Pd(1)-X(1)	92.82(3)	91.9982	94.00(4)	95.5845	91.61(6)	92.2628
N(1)-Pd(1)-X(1)	94.12(3)	94.4083	93.52(4)	91.6668	96.97(6)	96.3567
P(1)-Pd(1)-X(1)	173.939(12)	175.2124	171.784(11)	176.691	172.825(16)	176.4371
N(3)-P(1)-N(2)	84.24(5)	83.735	83.65(6)	83.9263	84.62(9)	84.181
N(3)-P(1)-C(23)	106.25(5)	105.482	106.08(7)	105.7241	109.69(11)	105.4953
N(2)-P(1)-C(23)	96.35(5)	95.6963	96.08(6)	95.6654	94.34(10)	95.6593
N(3)-P(1)-N(4)	83.71(5)	83.1186	84.35(6)	83.0654	83.86(9)	83.0778
N(2)-P(1)-N(4)	164.94(5)	164.1029	164.66(6)	164.1899	166.55(9)	164.0237
C(23)-P(1)-N(4)	95.66(5)	95.6963	96.40(6)	96.4782	96.05(10)	97.0016
N(3)-P(1)-Pd(1)	145.22(4)	145.4121	146.30(5)	143.8134	138.36(8)	144.5778
N(2)-P(1)-Pd(1)	92.85(3)	93.7187	92.17(4)	93.9356	93.48(7)	93.8071
C(23)-P(1)-Pd(1)	108.51(4)	109.092	107.61(5)	110.4277	111.93(8)	109.8901
N(4)-P(1)-Pd(1)	91.91(3)	92.0411	92.53(4)	91.276	90.54(7)	91.0125

Table S10. Compound $\mathbf{L^{Bn}{\bullet}Pd^{X}}$ (X = Br, I)

	$\mathbf{L^{Bn}{\bullet}Pd^{Br}}$		$\mathbf{L^{Bn}{\bullet}Pd^{I}}$	
	Expt.	Calc.	Expt.	Calc.
Pd(1)-X(1)	2.5536(3)	2.53552	2.7448(2)	2.7255
Pd(1)-P(1)	2.2197(6)	2.23461	2.2300(5)	2.2392
Pd(1)-N(1)	2.0591(17)	2.05964	2.0486(18)	2.03869
Pd(1)-N(5)	2.0377(18)	2.03622	2.0650(18)	2.07245
P(1)-N(3)	1.7399(19)	1.74751	1.7301(18)	1.74691
P(1)-N(2)	1.8410(17)	1.84146	1.8426(19)	1.83698
P(1)-N(4)	1.8598(18)	1.86227	1.856(2)	1.86176
P(1)-C(23)	1.847(2)	1.85839	1.848(2)	1.85733
N(5)-Pd(1)-N(1)	172.54(7)	173.134	171.19(7)	171.2192
N(5)-Pd(1)-P(1)	87.90(6)	88.1612	85.08(5)	84.3495
N(1)-Pd(1)-P(1)	84.90(5)	84.9908	87.67(5)	87.5875
N(5)-Pd(1)-X(1)	95.45(5)	94.5418	90.49(5)	93.6933
N(1)-Pd(1)-X(1)	91.70(5)	92.3236	97.47(5)	94.7723
P(1)-Pd(1)-X(1)	176.475(15)	175.6922	169.588(15)	171.7891
N(3)-P(1)-N(2)	83.51(8)	83.4973	84.11(9)	83.5546
N(3)-P(1)-C(23)	107.14(10)	105.4882	108.60(10)	105.5749
N(2)-P(1)-C(23)	99.45(9)	100.6671	93.85(9)	100.9728
N(3)-P(1)-N(4)	84.39(9)	93.695	83.57(8)	83.9544
N(2)-P(1)-N(4)	165.57(9)	164.1764	165.81(8)	164.1953
C(23)-P(1)-N(4)	91.60(9)	91.6845	96.74(9)	91.6854
N(3)-P(1)-Pd(1)	142.32(8)	144.8459	138.21(7)	146.003
N(2)-P(1)-Pd(1)	91.67(6)	91.9782	94.17(6)	92.1345
C(23)-P(1)-Pd(1)	110.51(7)	109.6037	113.18(7)	108.348
N(4)-P(1)-Pd(1)	93.12(6)	93.1527	90.34(6)	92.817

Table S11. Compound $\mathbf{L^{Ph}{\bullet}Pd^{I}}$

	$\mathbf{L^{Ph}{\bullet}Pd^{I}}$	
	Expt.	Calc.
Pd(1)-X(1)	2.7061(3)	2.72438
Pd(1)-P(1)	2.2301(5)	2.23831
Pd(1)-N(1)	2.0447(14)	2.03907
Pd(1)-N(5)	2.0645(14)	2.07532
P(1)-N(3)	1.7352(15)	1.74431
P(1)-N(2)	1.8442(15)	1.84738
P(1)-N(4)	1.8358(15)	1.8427
P(1)-C(23)	1.8235(16)	1.82546
N(5)-Pd(1)-N(1)	170.63(6)	170.7899
N(5)-Pd(1)-P(1)	83.55(4)	83.9856
N(1)-Pd(1)-P(1)	87.72(4)	87.3677
N(5)-Pd(1)-X(1)	91.91(4)	94.1102
N(1)-Pd(1)-X(1)	97.01(4)	94.8947
P(1)-Pd(1)-X(1)	174.128(12)	171.7142
N(3)-P(1)-N(2)	85.27(7)	84.3812
N(3)-P(1)-C(23)	106.50(7)	105.1598
N(2)-P(1)-C(23)	96.13(7)	95.5715
N(3)-P(1)-N(4)	84.11(7)	83.4838
N(2)-P(1)-N(4)	165.94(7)	164.3231
C(23)-P(1)-N(4)	95.71(7)	97.1534
N(3)-P(1)-Pd(1)	143.30(5)	146.7296
N(2)-P(1)-Pd(1)	93.07(5)	92.8088
C(23)-P(1)-Pd(1)	110.12(6)	108.1074
N(4)-P(1)-Pd(1)	89.96(5)	91.905

Table S12. Compound $\mathbf{L^{CCPh}{\bullet}Pd^{X}}$ (X = Br, I)

	$\mathbf{L^{CCPh}{\bullet}Pd^{Br}}$		$\mathbf{L^{CCPh}{\bullet}Pd^{I}}$	
	Expt.	Calc.	Expt.	Calc.
Pd(1)-X(1)	2.5362(3)	2.53042	2.7234(4)	2.72242
Pd(1)-P(1)	2.2171(5)	2.22567	2.2269(11)	2.23246
Pd(1)-N(1)	2.0612(16)	2.05675	2.042(3)	2.04462
Pd(1)-N(5)	2.0347(16)	2.03582	2.070(3)	2.06686
P(1)-N(3)	1.7331(16)	1.7421	1.727(3)	1.74129
P(1)-N(2)	1.8327(16)	1.84311	1.825(4)	1.82968
P(1)-N(4)	1.8211(16)	1.83361	1.846(3)	1.84003
P(1)-C(23)	1.764(2)	1.76004	1.774(4)	1.75884
N(5)-Pd(1)-N(1)	171.98(7)	172.3733	170.85(15)	171.2254
N(5)-Pd(1)-P(1)	87.58(5)	87.6117	84.17(11)	84.3967
N(1)-Pd(1)-P(1)	84.53(5)	85.1904	86.96(11)	87.1673
N(5)-Pd(1)-X(1)	96.03(5)	95.4801	91.75(10)	92.0032
N(1)-Pd(1)-X(1)	91.82(5)	91.7893	97.18(10)	96.5279
P(1)-Pd(1)-X(1)	176.065(14)	176.5046	175.48(3)	175.6132
N(3)-P(1)-N(2)	84.27(7)	84.3036	85.57(17)	83.9987
N(3)-P(1)-C(23)	107.23(9)	105.3612	107.95(19)	105.9443
N(2)-P(1)-C(23)	93.82(8)	94.8886	95.82(19)	95.2212
N(3)-P(1)-N(4)	85.07(8)	84.3036	83.65(16)	84.5225
N(2)-P(1)-N(4)	167.20(8)	166.5628	167.18(17)	166.461
C(23)-P(1)-N(4)	96.09(8)	94.6053	94.10(19)	94.815
N(3)-P(1)-Pd(1)	145.69(6)	144.2496	144.40(14)	144.8241
N(2)-P(1)-Pd(1)	91.10(6)	91.5816	94.08(12)	91.2502
C(23)-P(1)-Pd(1)	106.99(7)	110.3644	107.49(15)	109.1959
N(4)-P(1)-Pd(1)	93.72(6)	94.0045	90.66(12)	94.0071

b) Cartesian Coordinates for PBE0 Stationary Points

Cartesian coordinates of the PBE0/def2-TZVP stationary points in Å units

Table S13. Cartesian coordinates of $\mathbf{L^{Allyl}{\bullet}Pd^{Cl}}$

Pd	2.0765610000	2.6605430000	5.7247190000
Cl	0.7069910000	0.7139650000	5.6216940000
P	3.2866550000	4.5094150000	5.9907620000
N	1.2203740000	3.5394420000	4.1078830000
N	2.3276580000	5.4619640000	4.7342920000
N	4.6364230000	5.4458630000	5.3901320000
N	4.6219370000	3.5844620000	6.8998670000
N	3.0671760000	1.9634060000	7.3846010000
C	0.4187090000	2.8592480000	3.2745410000
H	0.2311690000	1.8333960000	3.5649750000
C	-0.1250930000	3.4131280000	2.1411490000
H	-0.7606800000	2.8161120000	1.5020350000
C	0.1868410000	4.7357900000	1.8494330000
H	-0.1976770000	5.2104360000	0.9535310000
C	0.9950110000	5.4501790000	2.6990790000
H	1.2630700000	6.4704250000	2.4748840000
C	1.5097340000	4.8364530000	3.8539150000
C	2.9020190000	6.7176620000	4.5424900000
C	2.3002670000	7.9090260000	4.1719380000
H	1.2390700000	7.9447100000	3.9628730000
C	3.0646550000	9.0689660000	4.1090150000
H	2.5998210000	10.0001620000	3.8084800000
C	4.4059970000	9.0394450000	4.4527050000
H	4.9937610000	9.9491780000	4.4193520000
C	5.0087120000	7.8591220000	4.8721240000
H	6.0407620000	7.8627150000	5.1923750000
C	4.2569970000	6.6934480000	4.9098960000
C	5.9226300000	5.0184750000	5.6854700000
C	7.1088900000	5.4686830000	5.1307890000
H	7.1077000000	6.2655860000	4.4002590000
C	8.3044250000	4.8543150000	5.4888280000
H	9.2350350000	5.2135080000	5.0666140000
C	8.3035330000	3.7779130000	6.3581860000
H	9.2347720000	3.2897570000	6.6196120000
C	7.1118790000	3.2955800000	6.8886850000
H	7.1245490000	2.4191520000	7.5214360000
C	5.9183090000	3.9292400000	6.5706670000
C	4.2317340000	2.5673470000	7.7115680000
C	4.9251450000	2.1877690000	8.8672040000

H	5.8182520000	2.7267530000	9.1482260000
C	4.4294620000	1.1718240000	9.6523830000
H	4.9607000000	0.8770140000	10.5503540000
C	3.2377400000	0.5526940000	9.2988470000
H	2.8134820000	-0.2507400000	9.8852920000
C	2.5830650000	0.9921130000	8.1699090000
H	1.6499630000	0.5583750000	7.8328490000
C	2.6492030000	5.4449270000	7.4565140000
H	2.9661570000	4.8775420000	8.3359600000
H	3.1685820000	6.4079450000	7.4817170000
C	1.1717080000	5.6367670000	7.4690320000
H	0.5726590000	4.7291850000	7.4397560000
C	0.5673290000	6.8154270000	7.5243160000
H	1.1305570000	7.7432980000	7.5483220000
H	-0.5133800000	6.8928910000	7.5474670000

Table S14. Cartesian coordinates of $\mathbf{L^{Allyl}{\bullet}Pd^{Br}}$

Pd	2.1105030000	2.6299100000	5.6876210000
Br	0.8581250000	0.4410780000	5.4513800000
P	3.2830560000	4.5034200000	5.9895560000
N	1.2548600000	3.5234860000	4.0695420000
N	2.3157150000	5.4590760000	4.7495190000
N	4.6311560000	5.4378200000	5.3834420000
N	4.6216050000	3.5711700000	6.8818790000
N	3.0588980000	1.9723170000	7.3954950000
C	0.4653710000	2.8550130000	3.2143140000
H	0.3007440000	1.8152810000	3.4654740000
C	-0.0945040000	3.4307260000	2.1001180000
H	-0.7190030000	2.8382380000	1.4459980000
C	0.1871270000	4.7675600000	1.8476760000
H	-0.2112960000	5.2618040000	0.9686520000
C	0.9833050000	5.4709130000	2.7171810000
H	1.2279100000	6.5030930000	2.5243350000
C	1.5135590000	4.8342350000	3.8522400000
C	2.8901660000	6.7174690000	4.5629610000
C	2.2858870000	7.9127800000	4.2104890000
H	1.2215140000	7.9532010000	4.0191790000
C	3.0529160000	9.0707390000	4.1416370000
H	2.5862190000	10.0050270000	3.8539650000
C	4.3998360000	9.0348550000	4.4621200000
H	4.9900730000	9.9427150000	4.4225190000

C	5.0053830000	7.8508870000	4.8668210000
H	6.0425130000	7.8491060000	5.1702180000
C	4.2500670000	6.6878580000	4.9116930000
C	5.9187750000	5.0027070000	5.6634340000
C	7.1028360000	5.4462430000	5.0991430000
H	7.1010990000	6.2449040000	4.3705040000
C	8.2969610000	4.8221300000	5.4457480000
H	9.2262700000	5.1750590000	5.0155320000
C	8.2970020000	3.7442050000	6.3135530000
H	9.2275500000	3.2497960000	6.5654680000
C	7.1070470000	3.2679800000	6.8531690000
H	7.1171400000	2.3902680000	7.4847090000
C	5.9160740000	3.9105000000	6.5448890000
C	4.2263860000	2.5733380000	7.7146490000
C	4.9119320000	2.2212250000	8.8830520000
H	5.8157330000	2.7521240000	9.1454900000
C	4.3933420000	1.2451480000	9.7034150000
H	4.9159250000	0.9718560000	10.6131810000
C	3.1882510000	0.6410550000	9.3698770000
H	2.7431850000	-0.1278680000	9.9863940000
C	2.5506690000	1.0447620000	8.2178650000
H	1.6143500000	0.6087900000	7.8950950000
C	2.6540690000	5.4287280000	7.4645580000
H	2.9859960000	4.8644630000	8.3404200000
H	3.1658770000	6.3960270000	7.4843190000
C	1.1755000000	5.6093240000	7.4946820000
H	0.5826150000	4.6976590000	7.4691770000
C	0.5634230000	6.7833300000	7.5627840000
H	1.1204700000	7.7149860000	7.5844490000
H	-0.5173900000	6.8532300000	7.5998790000

Table S15. Cartesian coordinates of **L^{Allyl}•PdI**

Pd	2.0650860000	2.6367960000	5.7111920000
I	0.6588370000	0.3112150000	5.5137780000
P	3.2661720000	4.5028090000	5.9969950000
N	1.2768550000	3.5200010000	4.0450830000
N	2.2834240000	5.4678540000	4.7811880000
N	4.6103740000	5.4397750000	5.3862800000
N	4.6102790000	3.5466470000	6.8479560000
N	3.0130320000	2.0122830000	7.4449170000
C	0.5541630000	2.8479990000	3.1340860000

H	0.3929620000	1.8022660000	3.3577440000
C	0.0468240000	3.4239290000	1.9954800000
H	-0.5241320000	2.8241360000	1.3003130000
C	0.3060190000	4.7706440000	1.7766200000
H	-0.0582280000	5.2686620000	0.8850030000
C	1.0401180000	5.4774980000	2.6960290000
H	1.2712310000	6.5176090000	2.5304170000
C	1.5270530000	4.8359660000	3.8474290000
C	2.8614060000	6.7272180000	4.5984650000
C	2.2526620000	7.9238550000	4.2603930000
H	1.1861380000	7.9625250000	4.0792640000
C	3.0190760000	9.0819100000	4.1890030000
H	2.5497180000	10.0183360000	3.9129990000
C	4.3703210000	9.0428470000	4.4920310000
H	4.9602070000	9.9508660000	4.4509330000
C	4.9811660000	7.8563660000	4.8805380000
H	6.0223940000	7.8523750000	5.1697820000
C	4.2255770000	6.6932940000	4.9281220000
C	5.8991270000	4.9895660000	5.6343340000
C	7.0777700000	5.4348000000	5.0601050000
H	7.0724600000	6.2493760000	4.3493170000
C	8.2711350000	4.7918100000	5.3732030000
H	9.1965540000	5.1455490000	4.9353130000
C	8.2757020000	3.6945190000	6.2166090000
H	9.2056230000	3.1858410000	6.4408010000
C	7.0905970000	3.2180000000	6.7664370000
H	7.1019560000	2.3258610000	7.3776380000
C	5.9015720000	3.8795760000	6.4936460000
C	4.2127170000	2.5770460000	7.7110540000
C	4.9272570000	2.2276760000	8.8629480000
H	5.8605590000	2.7281250000	9.0773800000
C	4.4010730000	1.2974010000	9.7296800000
H	4.9466400000	1.0264050000	10.6265620000
C	3.1588650000	0.7393860000	9.4595190000
H	2.7033310000	0.0095980000	10.1147110000
C	2.4979240000	1.1357320000	8.3185030000
H	1.5320030000	0.7294140000	8.0508050000
C	2.6620860000	5.4183350000	7.4877800000
H	3.0339430000	4.8677350000	8.3562410000
H	3.1503600000	6.3978820000	7.4871140000
C	1.1808990000	5.5625130000	7.5608720000
H	0.6108330000	4.6360650000	7.5584420000
C	0.5416660000	6.7212140000	7.6387060000
H	1.0750540000	7.6668400000	7.6374350000

H	-0.5390190000	6.7636410000	7.7069260000

Table S16. Cartesian coordinates of $\mathbf{L^{Bn}{\bullet}Pd^{Br}}$

Pd	7.0634770000	8.3727460000	3.0220350000
Br	9.4620810000	9.1830700000	3.1595870000
P	4.9431650000	7.6687570000	3.0683470000
N	6.3826260000	9.9291320000	4.1865990000
N	4.3151060000	9.3770180000	3.3482640000
N	3.4747800000	7.4948340000	2.1370190000
N	5.3678720000	6.0155170000	2.3237200000
N	7.5041060000	6.7616300000	1.8574170000
C	7.1653210000	10.6385180000	5.0114240000
H	8.2121110000	10.3649700000	4.9991300000
C	6.6869970000	11.6673440000	5.7905880000
H	7.3692040000	12.2210850000	6.4212510000
C	5.3295170000	11.9562280000	5.7428940000
H	4.9072040000	12.7382950000	6.3635820000
C	4.5095130000	11.2154430000	4.9244280000
H	3.4396510000	11.3617290000	4.9228840000
C	5.0605980000	10.1973570000	4.1399550000
C	3.2032370000	9.7355370000	2.6109370000
C	2.6414250000	10.9884750000	2.4087560000
H	3.0427450000	11.8628070000	2.9016920000
C	1.5671180000	11.1211650000	1.5356190000
H	1.1221430000	12.0983100000	1.3904300000
C	1.0812640000	10.0271950000	0.8412840000
H	0.2554730000	10.1426490000	0.1498890000
C	1.6660260000	8.7755930000	1.0048710000
H	1.3286750000	7.9387260000	0.4094970000
C	2.7110760000	8.6296680000	1.9012590000
C	3.1072110000	6.1732760000	1.9226340000
C	1.8303360000	5.6653140000	1.7247980000
H	0.9754010000	6.3238410000	1.6666640000
C	1.6547920000	4.2893450000	1.6402800000
H	0.6590160000	3.8931120000	1.4805000000
C	2.7275750000	3.4259530000	1.7890560000
H	2.5740940000	2.3544240000	1.7486380000
C	4.0036260000	3.9290730000	2.0161550000
H	4.8307990000	3.2531050000	2.1908700000
C	4.2000270000	5.3002700000	2.0508180000
C	6.5460060000	5.8238510000	1.6763110000

C	6.7896290000	4.7337860000	0.8232700000
H	6.0094420000	4.0108980000	0.6463640000
C	8.0039290000	4.6147220000	0.1938460000
H	8.1815980000	3.7765630000	-0.4707550000
C	8.9873360000	5.5715600000	0.4098360000
H	9.9594550000	5.5182110000	-0.0603140000
C	8.6883970000	6.6239420000	1.2402650000
H	9.4024170000	7.4101910000	1.4460430000
C	4.5793440000	6.8938930000	4.7178370000
H	3.9165720000	6.0539050000	4.4844590000
H	5.5322190000	6.4625280000	5.0310710000
C	3.9841670000	7.7241700000	5.8072630000
C	4.7572870000	8.1354630000	6.8896910000
H	5.8073510000	7.8644510000	6.9269890000
C	4.2008050000	8.8777940000	7.9208960000
H	4.8189580000	9.1819390000	8.7579540000
C	2.8592980000	9.2249480000	7.8832250000
H	2.4209640000	9.8002750000	8.6906200000
C	2.0774950000	8.8206740000	6.8089680000
H	1.0251970000	9.0797750000	6.7745560000
C	2.6350620000	8.0753020000	5.7829210000
H	2.0146650000	7.7561760000	4.9514340000

Table S17. Cartesian coordinates of $\mathbf{L^{Bn}{\bullet}Pd^{I}}$

Pd	6.9043770000	8.2705780000	3.0881040000
I	9.5168290000	8.9571330000	3.4515520000
P	4.7713190000	7.5893380000	3.0876460000
N	6.1935720000	9.8247000000	4.2605030000
N	4.1526550000	9.3014070000	3.3337410000
N	3.3095430000	7.4064820000	2.1487760000
N	5.2061280000	5.9276210000	2.3694710000
N	7.3103530000	6.7181790000	1.8305300000
C	6.9383750000	10.5279400000	5.1258690000
H	7.9784890000	10.2363710000	5.1827820000
C	6.4400050000	11.5727840000	5.8691670000
H	7.0992300000	12.1174310000	6.5314330000
C	5.0934990000	11.8878880000	5.7460640000
H	4.6532620000	12.6838120000	6.3358400000
C	4.3062790000	11.1523510000	4.8924430000
H	3.2406940000	11.3156400000	4.8329620000
C	4.8793000000	10.1167990000	4.1472760000

C	3.0620680000	9.6598050000	2.5646620000
C	2.5278380000	10.9167550000	2.3180890000
H	2.9373670000	11.7969850000	2.7935820000
C	1.4716090000	11.0443190000	1.4223600000
H	1.0476950000	12.0249080000	1.2419010000
C	0.9771790000	9.9402190000	0.7503750000
H	0.1650470000	10.0514620000	0.0423290000
C	1.5365440000	8.6833190000	0.9567640000
H	1.1935090000	7.8363150000	0.3791540000
C	2.5647720000	8.5443000000	1.8733850000
C	2.9454960000	6.0831640000	1.9421450000
C	1.6718340000	5.5714750000	1.7323940000
H	0.8165510000	6.2280550000	1.6571220000
C	1.5006550000	4.1944480000	1.6579550000
H	0.5077070000	3.7945440000	1.4899100000
C	2.5732320000	3.3337950000	1.8262370000
H	2.4216040000	2.2618120000	1.7919460000
C	3.8454500000	3.8413070000	2.0643410000
H	4.6752460000	3.1707590000	2.2494660000
C	4.0369690000	5.2126460000	2.0916830000
C	6.3587590000	5.7709160000	1.6653140000
C	6.5742620000	4.7090020000	0.7705990000
H	5.8022750000	3.9700990000	0.6256290000
C	7.7469580000	4.6393270000	0.0601990000
H	7.9032140000	3.8236000000	-0.6368070000
C	8.7139190000	5.6209550000	0.2344720000
H	9.6507580000	5.6121380000	-0.3051600000
C	8.4489090000	6.6340160000	1.1227280000
H	9.1622600000	7.4256110000	1.3043970000
C	4.3975300000	6.8368500000	4.7440650000
H	3.7227160000	6.0042150000	4.5187260000
H	5.3445530000	6.3947710000	5.0598600000
C	3.8173850000	7.6864270000	5.8264030000
C	4.6024700000	8.0993660000	6.8996750000
H	5.6486190000	7.8133320000	6.9343600000
C	4.0627620000	8.8632330000	7.9237310000
H	4.6900680000	9.1691550000	8.7532690000
C	2.7263470000	9.2298660000	7.8882070000
H	2.3012490000	9.8224580000	8.6901490000
C	1.9326170000	8.8229860000	6.8238750000
H	0.8841310000	9.0973000000	6.7918560000
C	2.4734590000	8.0564370000	5.8044380000
H	1.8441690000	7.7359930000	4.9801660000

Table S18. Cartesian coordinates of $\mathbf{L^{Ph} \bullet Pd^{I}}$

I	3.1613410000	8.1964180000	4.0137290000
Pd	3.6052770000	5.5187410000	3.7787480000
P	4.2841960000	3.3929520000	3.6052330000
N	3.0187620000	5.0775380000	5.6811500000
C	2.1903960000	5.8587730000	6.3930030000
H	1.8926320000	6.7766330000	5.9052460000
C	1.7493280000	5.5390630000	7.6537800000
H	1.0815860000	6.2111210000	8.1746790000
C	2.1809070000	4.3417170000	8.2106950000
H	1.8520000000	4.0392400000	9.1985740000
C	3.0242580000	3.5264590000	7.4972780000
H	3.3488920000	2.5805900000	7.9020660000
C	3.4435550000	3.9079230000	6.2121160000
N	4.2495340000	3.1372510000	5.4344990000
C	4.5500030000	1.8014840000	5.7244360000
C	5.1430740000	1.2762880000	6.8590010000
H	5.4181630000	1.9257470000	7.6807100000
C	5.4073210000	-0.0869890000	6.9204910000
H	5.8623780000	-0.5053880000	7.8099800000
C	5.1135600000	-0.9019410000	5.8391660000
H	5.3375490000	-1.9611720000	5.8846820000
C	4.5613590000	-0.3768470000	4.6771810000
H	4.3929570000	-1.0156160000	3.8217390000
C	4.2718860000	0.9790460000	4.6225520000
N	3.8168770000	1.7139090000	3.5341520000
C	3.2601820000	1.2370350000	2.3560630000
C	2.6062320000	0.0338110000	2.1532650000
H	2.5138370000	-0.6853720000	2.9550510000
C	2.0304600000	-0.2250780000	0.9133380000
H	1.5304340000	-1.1713650000	0.7464190000
C	2.0733910000	0.7259350000	-0.0909990000
H	1.6063470000	0.5263770000	-1.0479700000
C	2.6964200000	1.9518320000	0.1179510000
H	2.6744450000	2.7051310000	-0.6575100000
C	3.3127740000	2.2016150000	1.3360040000
N	3.9100540000	3.3562090000	1.8012930000
C	4.2066280000	4.4798950000	1.0942550000
C	4.5924420000	4.4570600000	-0.2499730000
H	4.6481030000	3.5094690000	-0.7656240000
C	4.9502150000	5.6309490000	-0.8719500000
H	5.2565890000	5.6197750000	-1.9118470000
C	4.9429180000	6.8142100000	-0.1465640000

H	5.2213270000	7.7605900000	-0.5897040000
C	4.5742000000	6.7670160000	1.1788380000
H	4.5496300000	7.6569350000	1.7935550000
N	4.1899830000	5.6387610000	1.7911190000
C	6.1038760000	3.3885720000	3.4601170000
C	6.8584680000	4.2957510000	4.1976160000
H	6.3581940000	4.9974310000	4.8566190000
C	8.2395040000	4.3105500000	4.0861660000
H	8.8182020000	5.0263860000	4.6584290000
C	8.8779350000	3.4111770000	3.2438680000
H	9.9586680000	3.4198800000	3.1594560000
C	8.1315130000	2.5019690000	2.5089640000
H	8.6267550000	1.7952540000	1.8529810000
C	6.7485190000	2.4945620000	2.6119420000
H	6.1699630000	1.7837390000	2.0319460000

Table S19. Cartesian coordinates of **L^{CCPh}•Pd^{Br}**

Br	0.0986170000	6.5269810000	10.1736930000
Pd	1.7730250000	7.7809800000	8.7500180000
P	3.3152910000	8.7680650000	7.4848370000
N	3.3072550000	6.5544920000	9.3599960000
C	3.3266150000	5.9237410000	10.5405310000
H	2.4307520000	6.0365320000	11.1366060000
C	4.3908890000	5.1536040000	10.9550150000
H	4.3457320000	4.6477460000	11.9097670000
C	5.4996310000	5.0597600000	10.1249080000
H	6.3678070000	4.4838160000	10.4252110000
C	5.5052160000	5.7297420000	8.9219460000
H	6.3765200000	5.7284780000	8.2832080000
C	4.3835700000	6.4769950000	8.5482160000
N	4.3184140000	7.2235130000	7.4131140000
C	4.8784950000	6.9337790000	6.1852780000
C	5.5101860000	5.7750550000	5.7559140000
H	5.6747520000	4.9488890000	6.4337650000
C	5.9038890000	5.6669090000	4.4264810000
H	6.4075660000	4.7665290000	4.0955730000
C	5.6388680000	6.6841460000	3.5268560000
H	5.9320990000	6.5841910000	2.4889510000
C	4.9712080000	7.8326260000	3.9406530000
H	4.7079300000	8.5949280000	3.2207280000
C	4.6114000000	7.9642560000	5.2705490000

N	3.8720950000	8.9892960000	5.8490050000
C	3.8306540000	10.3216560000	5.4476690000
C	4.6345100000	10.9601120000	4.5154770000
H	5.3783920000	10.4126130000	3.9550770000
C	4.5034820000	12.3325290000	4.3379150000
H	5.1286190000	12.8308360000	3.6064040000
C	3.6140030000	13.0684080000	5.1026910000
H	3.5409090000	14.1413210000	4.9725870000
C	2.8277460000	12.4363590000	6.0594190000
H	2.1761610000	13.0169350000	6.6998080000
C	2.9137180000	11.0631790000	6.2082530000
N	2.2736000000	10.2359190000	7.1349570000
C	0.9314930000	10.2012570000	7.3411650000
C	0.0485010000	11.1598100000	6.8168260000
H	0.4345470000	11.9590550000	6.2044410000
C	-1.2983310000	11.0547540000	7.0629650000
H	-1.9764600000	11.7927200000	6.6492390000
C	-1.7773250000	10.0037310000	7.8345910000
H	-2.8280630000	9.8834930000	8.0591570000
C	-0.8700590000	9.0867050000	8.3066730000
H	-1.1721520000	8.2360750000	8.9036860000
N	0.4477500000	9.1670790000	8.0666780000
C	4.4626300000	9.6454930000	8.4905630000
C	5.2801470000	10.2410210000	9.1549560000
C	6.2441410000	10.9589940000	9.9115340000
C	6.1690520000	12.3526680000	10.0051810000
H	5.3626470000	12.8725960000	9.5025870000
C	7.1191080000	13.0534650000	10.7264700000
H	7.0544340000	14.1334060000	10.7910960000
C	8.1516880000	12.3789440000	11.3637800000
H	8.8946270000	12.9315580000	11.9271450000
C	8.2315050000	10.9957370000	11.2775570000
H	9.0366690000	10.4662920000	11.7736070000
C	7.2874420000	10.2866600000	10.5562720000
H	7.3463950000	9.2075660000	10.4817540000

Table S20. Cartesian coordinates of **L^{CCPh}•Pd^{I}**

I	0.1693740000	6.5651580000	10.2517020000
Pd	1.9277490000	7.9198480000	8.6754740000
P	3.4518470000	8.8828600000	7.3588030000
N	3.5064260000	6.7397030000	9.2975230000
C	3.5943620000	6.1743070000	10.5082180000

H	2.7375180000	6.3197300000	11.1515610000
C	4.6784860000	5.4231600000	10.9051920000
H	4.6839910000	4.9706860000	11.8873890000
C	5.7377860000	5.2750980000	10.0206680000
H	6.6178500000	4.7073320000	10.3012840000
C	5.6792030000	5.8835540000	8.7871210000
H	6.5131460000	5.8441090000	8.1013820000
C	4.5423200000	6.6201250000	8.4393200000
N	4.4286220000	7.3253800000	7.2822510000
C	4.9580830000	7.0133280000	6.0468040000
C	5.5548230000	5.8368270000	5.6164650000
H	5.7129600000	5.0132380000	6.2992900000
C	5.9204450000	5.7091870000	4.2807510000
H	6.3973040000	4.7951710000	3.9475650000
C	5.6607620000	6.7250680000	3.3777340000
H	5.9321160000	6.6098170000	2.3354710000
C	5.0266860000	7.8918070000	3.7934040000
H	4.7676890000	8.6540140000	3.0718300000
C	4.6949160000	8.0417780000	5.1286480000
N	3.9884850000	9.0851440000	5.7146670000
C	3.9449680000	10.4124800000	5.2994480000
C	4.7325090000	11.0372180000	4.3441910000
H	5.4623050000	10.4801010000	3.7746820000
C	4.6037560000	12.4082480000	4.1554980000
H	5.2160860000	12.8965240000	3.4066160000
C	3.7331340000	13.1560670000	4.9306810000
H	3.6625830000	14.2279510000	4.7913880000
C	2.9632390000	12.5374000000	5.9090980000
H	2.3250550000	13.1265200000	6.5553850000
C	3.0462770000	11.1656930000	6.0698180000
N	2.4153560000	10.3511980000	7.0161790000
C	1.0702500000	10.3056790000	7.2092200000
C	0.1896340000	11.2517480000	6.6591470000
H	0.5819740000	12.0474320000	6.0461650000
C	-1.1608410000	11.1390610000	6.8786540000
H	-1.8367310000	11.8671350000	6.4443370000
C	-1.6447040000	10.0899500000	7.6490570000
H	-2.6984700000	9.9583130000	7.8521820000
C	-0.7379350000	9.1887390000	8.1507870000
H	-1.0526990000	8.3444010000	8.7491750000
N	0.5854200000	9.2771180000	7.9430550000
C	4.6121070000	9.7505970000	8.3559750000
C	5.4200700000	10.3254970000	9.0496820000
C	6.3650710000	11.0128360000	9.8566780000

C	6.2372580000	12.3884250000	10.0757580000
H	5.4075220000	12.9197250000	9.6258770000
C	7.1635000000	13.0574640000	10.8556970000
H	7.0571780000	14.1236140000	11.0187490000
C	8.2244950000	12.3686270000	11.4275240000
H	8.9482820000	12.8963370000	12.0376840000
C	8.3570380000	11.0030040000	11.2164130000
H	9.1841100000	10.4624020000	11.6617730000
C	7.4368150000	10.3258960000	10.4362340000
H	7.5354990000	9.2606190000	10.2662240000

c) Natural localized molecular orbital analysis

Table S21. Composition of NLMOs representing Pd-P bonds in $\mathbf{L^{R}{\bullet}Pd^{X}}$ (R = Allyl, Bn, Ph, CCPh; X = Cl, Br, I). The s-, d-, and p-characters are represented in percentages (%).

	$\mathbf{L^{Allyl}{\bullet}Pd^{Cl}}$	$\mathbf{L^{Allyl}{\bullet}Pd^{Br}}$	$\mathbf{L^{Allyl}{\bullet}Pd^{I}}$	$\mathbf{L^{Bn}{\bullet}Pd^{Br}}$	$\mathbf{L^{Bn}{\bullet}Pd^{I}}$	$\mathbf{L^{Ph}{\bullet}Pd^{I}}$	$\mathbf{L^{CCPh}{\bullet}Pd^{Br}}$	$\mathbf{L^{CCPh}{\bullet}Pd^{I}}$
% Pd	44.053	45.052	45.996	44.927	45.921	45.850	43.935	44.906
% P	53.613	52.639	51.677	52.768	51.731	51.708	53.446	52.409
Pd %s	13.06	13.09	12.93	13.1	12.92	12.72	13.15	13.00
Pd %d	86.85	86.82	86.99	86.81	87.00	87.20	86.76	86.92
P %s	44.11	43.8	43.56	44.08	43.84	42.80	42.49	42.13
P %p	55.25	55.55	55.79	55.27	55.51	56.58	56.89	57.25

Table S22. Composition of NLMOs representing P-C bonds in $\mathbf{L^{R}{\bullet}Pd^{X}}$ (R = Allyl, Bn, Ph, CCPh; X = Cl, Br, I). The s-, d-, and p-characters are represented in percentages (%).

	$\mathbf{L^{Allyl}{\bullet}Pd^{Cl}}$	$\mathbf{L^{Allyl}{\bullet}Pd^{Br}}$	$\mathbf{L^{Allyl}{\bullet}Pd^{I}}$	$\mathbf{L^{Bn}{\bullet}Pd^{Br}}$	$\mathbf{L^{Bn}{\bullet}Pd^{I}}$	$\mathbf{L^{Ph}{\bullet}Pd^{I}}$	$\mathbf{L^{CCPh}{\bullet}Pd^{Br}}$	$\mathbf{L^{CCPh}{\bullet}Pd^{I}}$
% P	37.514	37.513	37.534	37.511	37.551	36.860	35.728	35.728
% C	60.474	60.478	60.449	60.326	60.288	61.194	62.905	62.902
P %s	34.43	34.76	34.78	34.95	35.08	34.36	31.59	31.65
P %p	64.91	64.57	64.56	64.39	64.26	64.90	67.45	67.39
C %s	24.62	24.63	24.60	23.97	24.04	27.43	42.44	42.42
C %p	75.01	75.00	75.02	75.7	75.63	72.32	57.35	57.37

Table S23. Visualization of P-C and Pd-P bond NLMOs of **L^{R}•Pd^{I}**(R = Allyl, Bn, Ph, CCPh) with IboView. Each orbital isosurface contains 80% of the integrated electron density of the orbirals.

	**Pd-P bond**	**P-C bond**
**L^{Allyl}•Pd^{I}**	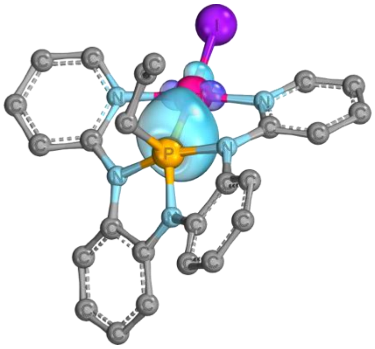	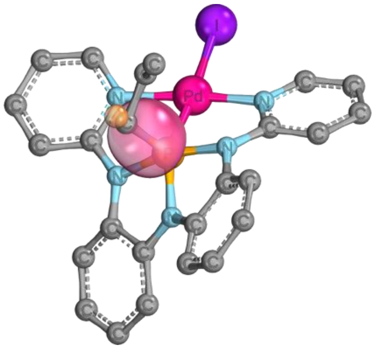
**L^{Bn}•Pd^{I}**	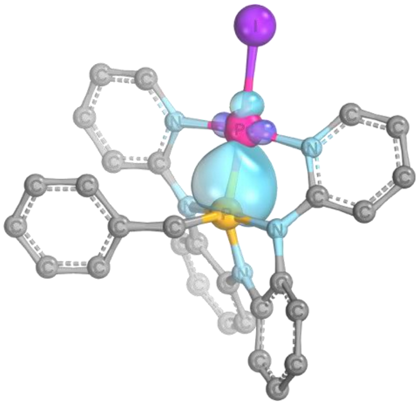	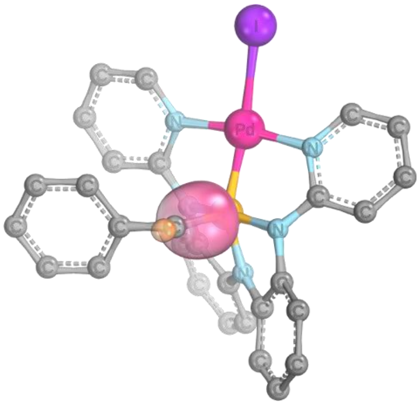
**L^{Ph}•Pd^{I}**	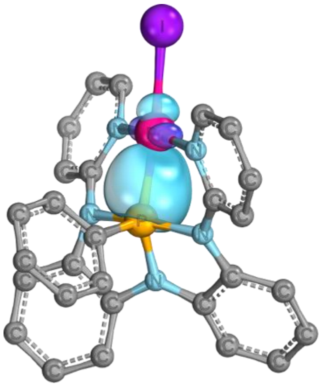	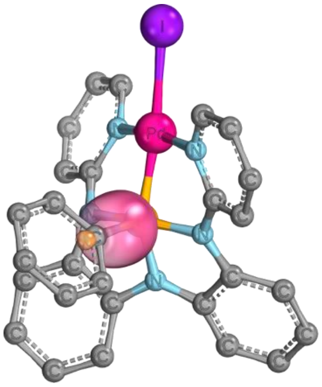
**L^{CCPh}•Pd^{I}**	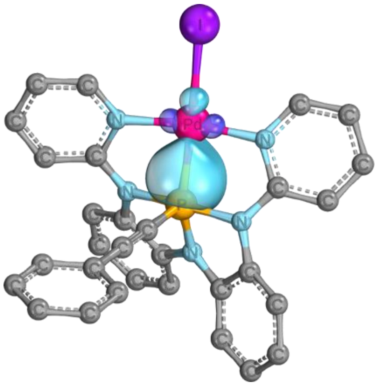	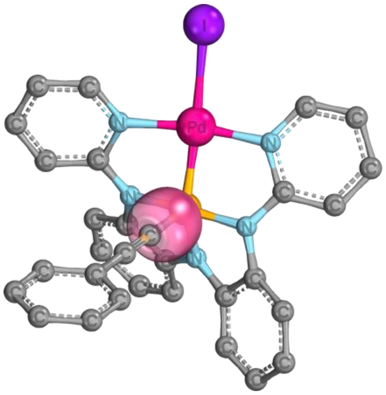

VI. Details of Low Temperature In-Situ ^{31}P NMR Experiments

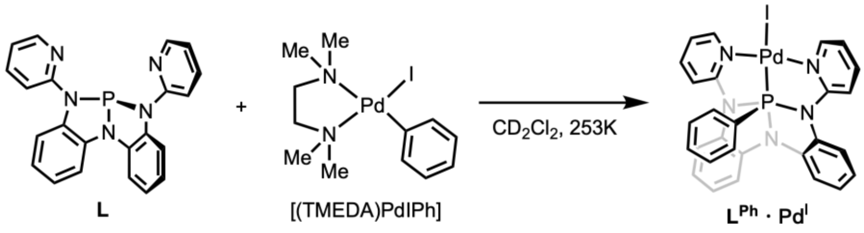

In an N_2 glovebox, **L** (6.0 mg, 0.016 mmol) was dissolved in CD_2Cl_2 (0.5 mL), and the resulting clear solution was transferred into an NMR tube with a screw-thread cap with silicon septum. [(TMEDA)PdI(C_6H_5)] (6.7 mg, 0.016 mmol) was dissolved in CD_2Cl_2 (0.5 mL) and the resulting orange-red solution was transferred into a 1 mL plastic syringe with a needle. Both solutions were then transferred out of the glovebox and stored in an ice bucket packed with dry ice. A $^{31}P\{^1H\}$ spectrum of the CD_2Cl_2 solution of **L** was taken at 253K on a 500MHz NMR spectrometer. After withdrawing the NMR tube from the spectrometer, the CD_2Cl_2 solution of [(TMEDA)PdI(C_6H_5)] was quickly injected into the NMR sample via the silicone septum. Subsequently, time course $^{31}P\{^1H\}$ NMR spectra (Figure S44) of the resulting orange mixture were acquired at 253K to monitor the formation of $\mathbf{L^{Ph}{\bullet}Pd^{I}}$.

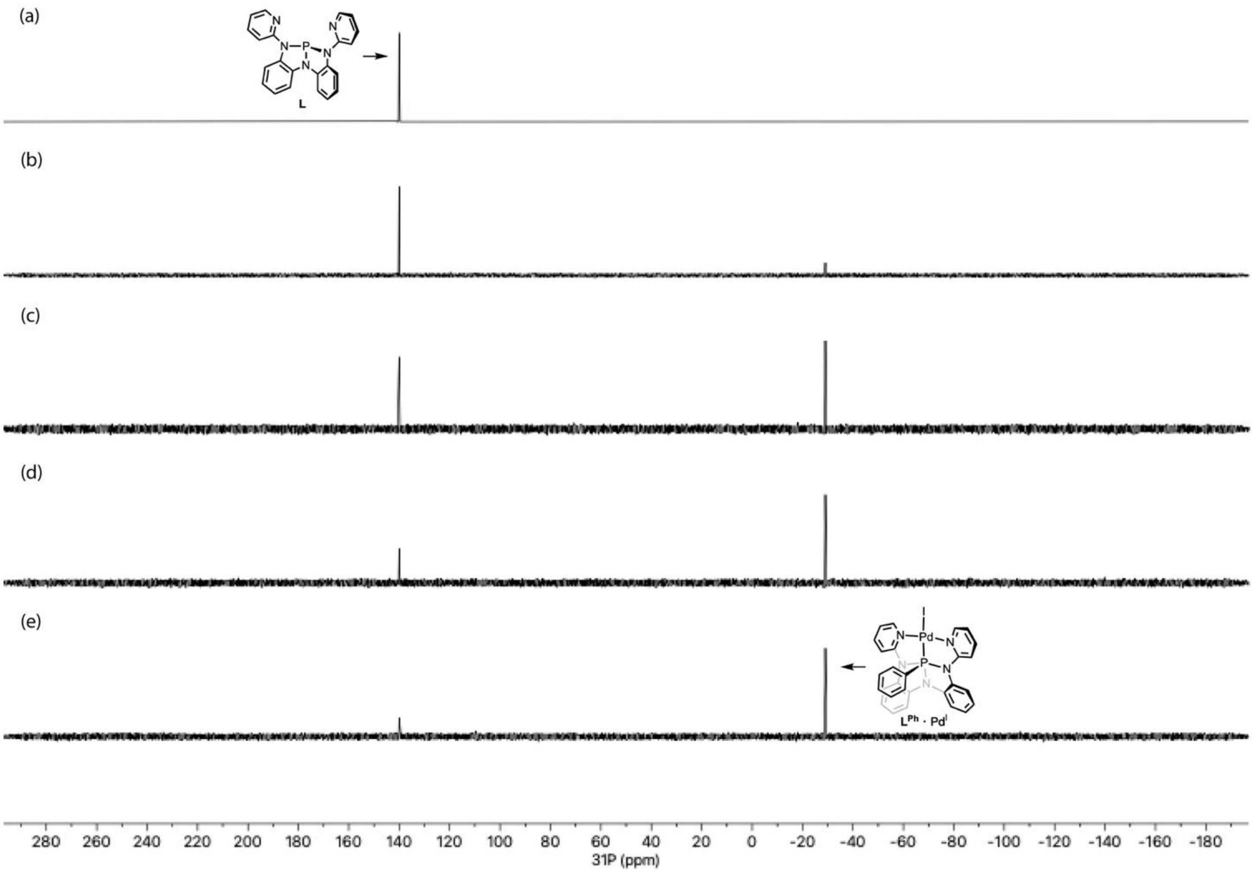

Figure S44. Time course $^{31}P\{^{1}H\}$ NMR spectra (202 MHz, CD_2Cl_2) monitoring the formation of $\mathbf{L^{Ph}•Pd^{I}}$ at 253K. (a) **L** only. (b) 5 min (c) 25 min (d) 45 min (e) 65 min after the addition of [(TMEDA)PdI(C_6H_5)] to **L**.

VII. Computational Details for Mechanistic Studies

General Information

All structure optimizations and single point energies were calculated using the Orca 6.1.0 program package.[S19] Computed molecular structures were visualized with CYLview.[S20] Intrinsic bond orbitals (IBOs) were plotted using IboView v20211019-RevA.[S18,S21]

Reaction Profiles

The composite method r^{2}SCAN-3c[S22] was applied for geometry optimization and frequency calculations. Stationary points were confirmed as either local minima (zero imaginary frequencies) or first order saddle points (one imaginary frequency for transition states). Thermochemistry data to obtain Gibbs free energies under standard conditions (298.15 K and 1 atm) were obtained using the rigid-rotor harmonic oscillator (RRHO) approximation and the Quasi-RRHO approach by Grimme[S23] for low frequencies as implemented in the default settings of Orca 6.1.0. For transition state structures, an intrinsic reaction coordinate (IRC)[S24] calculation was conducted to ensure transit between desired reactant and product minima.

Relaxed Surface Scan

The composite method r^{2}SCAN-3c[S22] was applied for constrained geometry optimization across two variables corresponding to the Pd---N(pyr) distance (3.416 A > d > 2.055 A) and P---C(phenyl) distance (2.991 A > d > 1.826 A). Ten increments were selected for each variable, corresponding to a two-dimensional grid comprising 100 data points.

a) Cartesian Coordinates for r^{2}scan-3c Stationary Points

Table S24. Cartesian coordinates of **L•Pd^{PhCI}**.

Pd	6.0221234453	10.0831992278	10.0954666139
Cl	8.0616184546	10.4096671704	11.2164941387
P	4.1746477955	9.6297552134	9.0415154757
N	5.9291216089	10.3362406574	6.7994090958
C	7.0392777171	10.8403586815	6.2507015416
H	7.8773409578	10.1522499745	6.1541949645
C	7.1449237138	12.1556842028	5.8209905959
H	8.0639198463	12.5138027636	5.3691706264
C	6.0547197032	13.0003679796	6.0136571391
H	6.1120740460	14.0493395603	5.7388580594
C	4.9055018500	12.5072092155	6.6084517950
H	4.0739506640	13.1576664347	6.8547086436
C	4.8906653085	11.1586571863	6.9670355026
N	3.8085402464	10.5674059010	7.6446887262
C	2.4591500004	10.9398963857	7.5714040326
C	1.8394628485	11.6930859519	6.5827994006
H	2.4023364767	12.0593464332	5.7316887665
C	0.4741348433	11.9522309113	6.6952252157
H	-0.0158493866	12.5486607248	5.9321453275
C	-0.2574812263	11.4590816725	7.7689003762
H	-1.3171062027	11.6775064456	7.8522399659
C	0.3579391697	10.6783865332	8.7482242983
H	-0.2190439104	10.2976017249	9.5810005295
C	1.7118501766	10.4056872286	8.6348406724
N	2.4943794441	9.5769517946	9.4817776857
C	2.1006403777	8.2472961544	9.7938483039
C	0.9271888541	7.8609057720	10.4197675759
H	0.2113517905	8.6015014497	10.7533185554
C	0.6969020446	6.5046896692	10.6531897603
H	-0.2221073173	6.1950818417	11.1398412322
C	1.6504296360	5.5615657017	10.2952310110
H	1.4789222436	4.5099223053	10.5020932621
C	2.8467421443	5.9454975268	9.6876180956
H	3.5936826568	5.1992878080	9.4452935629
C	3.0660067217	7.2915851778	9.4289525706
N	4.2291895614	7.8896332152	8.8840101122
C	5.4981930265	7.2854752977	8.9472573725
C	5.7547349632	6.0660832546	8.3252598329
H	4.9815948870	5.5897803722	7.7334502656
C	7.0216566451	5.5136926270	8.4434499754
H	7.2447820994	4.5627664707	7.9687012865

C	8.0018108463	6.2030909387	9.1481859595
H	9.0045746729	5.8058317856	9.2606107163
C	7.6863714859	7.4382043076	9.6930045904
H	8.4115337103	8.0491785568	10.2272190392
N	6.4570806538	7.9668549872	9.6062205862
H	2.9913916766	14.1997994583	10.7827900219
H	3.5424313148	11.8140648583	10.9520452361
C	3.9746913070	13.8572027235	10.4701251376
C	4.2897343137	12.5004976610	10.5600947887
C	4.9111820382	14.7639335866	9.9849352532
H	4.6668529822	15.8204623471	9.9160665434
C	5.5482293776	12.0342491456	10.1666864580
C	6.1693209659	14.3061683111	9.6028830655
C	6.4888291698	12.9525991528	9.6918901432
H	6.9161884519	15.0069190616	9.2369723703
H	7.4789784701	12.6145401125	9.4023492447

Table S25. Cartesian coordinates of **L•Pd^{PhCl}-iso**.

Pd	6.2718100000	10.0700600000	10.2233000000
Cl	5.6157500000	12.2482200000	10.3506900000
P	4.2475400000	9.2681200000	9.5864300000
N	5.3884400000	9.2936100000	6.9865100000
C	6.2896700000	9.5434700000	6.0433800000
H	7.1577000000	8.8911300000	6.0304400000
C	6.1543300000	10.5599000000	5.1155100000
H	6.9054800000	10.7119600000	4.3513300000
C	5.0417500000	11.3842500000	5.2133800000
H	4.9127400000	12.2185900000	4.5336300000
C	4.1088100000	11.1593100000	6.2063200000
H	3.2705600000	11.8259200000	6.3483100000
C	4.3196700000	10.0797900000	7.0622200000
N	3.4846600000	9.7559200000	8.1299200000
C	2.1188300000	10.0011800000	8.2814800000
C	1.2099800000	10.4366300000	7.3322200000
H	1.5149700000	10.6112400000	6.3098100000
C	-0.1154200000	10.6281600000	7.7071700000
H	-0.8268400000	10.9812100000	6.9703600000
C	-0.5265600000	10.3838400000	9.0055800000
H	-1.5568300000	10.5555100000	9.2918900000
C	0.3774700000	9.9160300000	9.9534100000
H	0.0521600000	9.7310700000	10.9668600000
C	1.6906900000	9.7048300000	9.5831500000

N	2.7236200000	9.1550800000	10.3710100000
C	2.5749700000	7.9165000000	11.0327900000
C	1.5829200000	7.5658000000	11.9266300000
H	0.8280000000	8.2817200000	12.2173700000
C	1.5846500000	6.2886000000	12.4784500000
H	0.8072100000	6.0099400000	13.1790700000
C	2.5844600000	5.3899400000	12.1554600000
H	2.5942900000	4.4039000000	12.6041100000
C	3.5971800000	5.7410400000	11.2679600000
H	4.3869500000	5.0370000000	11.0461500000
C	3.5877400000	7.0038200000	10.7042600000
N	4.5417500000	7.5834000000	9.8476000000
C	5.8576500000	7.1505700000	9.7155800000
C	6.1574700000	5.8482300000	9.3355000000
H	5.3518400000	5.1657700000	9.1041100000
C	7.4794600000	5.4675500000	9.2137400000
H	7.7284200000	4.4547100000	8.9192000000
C	8.4719500000	6.4049400000	9.4454500000
H	9.5216300000	6.1583500000	9.3570700000
C	8.1006100000	7.6908000000	9.7836500000
H	8.8348000000	8.4645200000	9.9645300000
N	6.8248000000	8.0648700000	9.9312400000
H	10.2277800000	9.9125100000	13.4661800000
H	7.9672200000	9.3783100000	12.6789000000
C	9.8696600000	10.3423800000	12.5360900000
C	8.5871200000	10.0456500000	12.0871300000
C	10.6866400000	11.1886200000	11.8009400000
H	11.6879600000	11.4197600000	12.1473000000
C	8.0940200000	10.5947800000	10.9037100000
C	10.2048000000	11.7482700000	10.6260600000
C	8.9164800000	11.4633500000	10.1887100000
H	10.8305100000	12.4231800000	10.0506700000
H	8.5098200000	11.5617200000	8.8172100000

Table S26. Cartesian coordinates of **TS-I**.

Pd	6.2725500000	10.2297600000	10.1475400000
Cl	8.3060400000	10.8411100000	11.2303900000
P	4.4137200000	9.9582900000	9.2125000000
N	6.0617400000	9.9150100000	6.8588200000
C	7.1616100000	10.0513700000	6.1175000000
H	7.9261000000	9.2883400000	6.2583400000
C	7.3481000000	11.0899100000	5.2146100000

H	8.2533800000	11.1505600000	4.6204500000
C	6.3521000000	12.0578700000	5.1242300000
H	6.4717900000	12.9122200000	4.4641600000
C	5.2121900000	11.9525200000	5.9068600000
H	4.4695800000	12.7394100000	5.9082900000
C	5.0984800000	10.8411200000	6.7527700000
N	4.0360100000	10.6162800000	7.6296100000
C	2.7454800000	11.1651100000	7.6321600000
C	2.1119100000	11.9154100000	6.6458800000
H	2.5862600000	12.1094500000	5.6928400000
C	0.8192000000	12.3867700000	6.8799000000
H	0.3309600000	12.9777300000	6.1110400000
C	0.1612800000	12.1243500000	8.0739900000
H	-0.8301100000	12.5271900000	8.2531200000
C	0.7725700000	11.3315100000	9.0465300000
H	0.2715000000	11.1179300000	9.9837300000
C	2.0360800000	10.8268500000	8.8048600000
N	2.7365200000	9.9010100000	9.6377700000
C	2.2599800000	8.5588400000	9.7332300000
C	0.9854600000	8.1859300000	10.1243600000
H	0.2376700000	8.9369400000	10.3486700000
C	0.6789000000	6.8302600000	10.2457800000
H	-0.3204300000	6.5334100000	10.5468400000
C	1.6551700000	5.8705900000	10.0141300000
H	1.4248600000	4.8177300000	10.1436300000
C	2.9420100000	6.2384200000	9.6218500000
H	3.6957400000	5.4760900000	9.4695900000
C	3.2382300000	7.5874500000	9.4501500000
N	4.4588100000	8.1778500000	9.0879300000
C	5.7027300000	7.5438300000	9.0506200000
C	5.8958000000	6.3155000000	8.4154400000
H	5.0757400000	5.8446600000	7.8870300000
C	7.1624600000	5.7508700000	8.4170900000
H	7.3257800000	4.7982600000	7.9215000000
C	8.2188100000	6.4217800000	9.0257000000
H	9.2207800000	6.0076600000	9.0443100000
C	7.9686000000	7.6609600000	9.5936500000
H	8.7420000000	8.2677600000	10.0623100000
N	6.7447800000	8.2026100000	9.6122000000
H	3.0116400000	13.3619000000	12.5629700000
H	3.8679600000	11.1246800000	11.9705900000
C	3.6067700000	13.2321200000	11.6634100000
C	4.0972600000	11.9750800000	11.3340800000
C	3.8789700000	14.3224600000	10.8415900000

H	3.4889400000	15.3041800000	11.0953500000
C	4.8736500000	11.7852400000	10.1787100000
C	4.6528700000	14.1561900000	9.6955800000
C	5.1456500000	12.9000300000	9.3676500000
H	4.8817100000	15.0095600000	9.0632400000
H	5.7629700000	12.7824800000	8.4818800000

Table S27. Cartesian coordinates of **INT**.

Pd	6.2446600000	10.2804400000	10.1224900000
Cl	8.2628800000	10.9969700000	11.1730600000
P	4.3172500000	10.0336200000	9.2322000000
N	6.0314200000	9.8995300000	6.8377700000
C	7.1296000000	10.0409700000	6.0954800000
H	7.8991500000	9.2835200000	6.2424000000
C	7.3126800000	11.0743500000	5.1850800000
H	8.2204400000	11.1395700000	4.5949500000
C	6.3031900000	12.0273600000	5.0798200000
H	6.4123500000	12.8784300000	4.4133400000
C	5.1594800000	11.9105800000	5.8546000000
H	4.4029400000	12.6844400000	5.8375300000
C	5.0546300000	10.8097200000	6.7178100000
N	3.9862000000	10.5934500000	7.5917800000
C	2.6787900000	11.1050500000	7.5491800000
C	2.0501300000	11.8130700000	6.5282100000
H	2.5463400000	11.9981700000	5.5843200000
C	0.7362300000	12.2483900000	6.7091300000
H	0.2565100000	12.8064100000	5.9107800000
C	0.0470900000	11.9921800000	7.8870100000
H	-0.9613400000	12.3680300000	8.0257400000
C	0.6520200000	11.2406100000	8.8952900000
H	0.1279500000	11.0294000000	9.8210400000
C	1.9381800000	10.7697400000	8.7078700000
N	2.6251700000	9.8744800000	9.5873400000
C	2.1841800000	8.5269000000	9.6831600000
C	0.9110600000	8.1203700000	10.0460000000
H	0.1355400000	8.8541500000	10.2323300000
C	0.6397400000	6.7586000000	10.1825600000
H	-0.3600100000	6.4361200000	10.4566300000
C	1.6529500000	5.8258200000	9.9972700000
H	1.4537400000	4.7675400000	10.1350200000
C	2.9392200000	6.2282000000	9.6407900000
H	3.7184800000	5.4859900000	9.5244900000

C	3.2019800000	7.5824300000	9.4513100000
N	4.4107200000	8.2076400000	9.1286400000
C	5.6575700000	7.6120700000	9.0863900000
C	5.8929000000	6.3715100000	8.4857700000
H	5.0827600000	5.8609100000	7.9808500000
C	7.1752100000	5.8487600000	8.4862400000
H	7.3611900000	4.8885100000	8.0137800000
C	8.2177700000	6.5682600000	9.0659000000
H	9.2324600000	6.1850100000	9.0885200000
C	7.9362000000	7.8157500000	9.5970800000
H	8.6932900000	8.4604300000	10.0397400000
N	6.6918300000	8.3197700000	9.6096900000
H	3.5045600000	13.4208900000	12.8895600000
H	3.6094100000	11.1451800000	11.9119600000
C	3.9367200000	13.2710700000	11.9035800000
C	3.9974200000	11.9961300000	11.3580500000
C	4.4313100000	14.3602700000	11.1860900000
H	4.3767700000	15.3596100000	11.6126500000
C	4.5103000000	11.7998200000	10.0638200000
C	5.0003500000	14.1714700000	9.9294800000
C	5.0434400000	12.8981500000	9.3688000000
H	5.4053500000	15.0193300000	9.3840700000
H	5.4490200000	12.7612800000	8.3696500000

Table S28. Cartesian coordinates of **TS-II**.

Pd	2.1856200000	0.3867200000	0.1786000000
Cl	4.5068800000	1.0027800000	0.3793400000
P	-0.0475400000	0.0999600000	0.1283500000
N	1.8089500000	0.6372800000	-2.2457100000
C	2.9182100000	1.0617700000	-2.8726400000
H	3.7258300000	0.3379100000	-2.9427400000
C	3.0518900000	2.3395200000	-3.3890100000
H	3.9784300000	2.6382300000	-3.8683300000
C	1.9886400000	3.2246400000	-3.2444300000
H	2.0622900000	4.2435100000	-3.6122400000
C	0.8391500000	2.8086500000	-2.5877300000
H	0.0052200000	3.4809200000	-2.4201400000
C	0.7979800000	1.5023300000	-2.0946300000
N	-0.3152000000	1.0469000000	-1.3478100000
C	-1.6213900000	1.0482900000	-1.8419500000
C	-2.0728700000	1.5767000000	-3.0459800000
H	-1.3903100000	2.0819700000	-3.7183500000

C	-3.4188600000	1.4330000000	-3.3835300000
H	-3.7824600000	1.8681700000	-4.3094700000
C	-4.2809600000	0.7308100000	-2.5530600000
H	-5.3247300000	0.6117700000	-2.8284700000
C	-3.8233500000	0.1546900000	-1.3668100000
H	-4.4998600000	-0.4348800000	-0.7652700000
C	-2.4901200000	0.3271500000	-1.0001700000
N	-1.8041600000	-0.2193000000	0.0785500000
C	-2.2210500000	-1.2127600000	0.9600800000
C	-3.4932800000	-1.4284600000	1.4822100000
H	-4.3212400000	-0.7863600000	1.2114600000
C	-3.6828000000	-2.4502900000	2.4124600000
H	-4.6745700000	-2.6191000000	2.8192900000
C	-2.6128100000	-3.2212200000	2.8509600000
H	-2.7624300000	-3.9874600000	3.6048900000
C	-1.3295800000	-2.9929500000	2.3574200000
H	-0.4846100000	-3.5529700000	2.7422700000
C	-1.1410400000	-2.0082600000	1.3957900000
N	0.0748900000	-1.5645700000	0.8661900000
C	1.1871900000	-2.3515500000	0.6296800000
C	1.1763300000	-3.7538000000	0.6380500000
H	0.2557300000	-4.2920400000	0.8187200000
C	2.3549000000	-4.4304100000	0.3716000000
H	2.3579300000	-5.5165000000	0.3755900000
C	3.5200200000	-3.7226200000	0.0877100000
H	4.4575800000	-4.2235400000	-0.1266600000
C	3.4564900000	-2.3417900000	0.0655700000
H	4.3130900000	-1.7099000000	-0.1555100000
N	2.3184600000	-1.6817200000	0.3314500000
C	0.2469400000	1.2927500000	1.4838600000
C	0.5483200000	2.6287500000	1.1475200000
H	0.3958500000	2.9806900000	0.1340000000
C	1.0003200000	3.5152100000	2.1233000000
H	1.2566100000	4.5316700000	1.8417700000
C	1.1282300000	3.1009700000	3.4401200000
H	1.4928600000	3.7916700000	4.1964800000
C	0.7918300000	1.7918700000	3.7926300000
H	0.8742600000	1.4749000000	4.8278000000
C	0.3745200000	0.8853800000	2.8296900000
H	0.1438000000	-0.1350800000	3.1172200000

Table S29. Cartesian coordinates of $\mathbf{L^{Ph}{\bullet}Pd^{CI}}$.

Cl	2.9820379402	8.1588182539	3.9310003198
Pd	3.4989038433	5.8100657324	3.7926495839
P	4.1974331078	3.6907152361	3.6280749421
N	3.0073405485	5.4208077387	5.7460675697
C	2.2707073497	6.2749537332	6.4856218267
H	2.0177198963	7.2080497925	5.9936627074
C	1.8632530684	5.9886921459	7.7711784841
H	1.2687177093	6.7105951678	8.3182884523
C	2.2166608936	4.7543921223	8.3151022763
H	1.8911497034	4.4753961381	9.3132363316
C	2.9766604414	3.8727130375	7.5779695342
H	3.2368531717	2.9006557769	7.9743862373
C	3.3879031023	4.2236570137	6.2766206414
N	4.1442682610	3.4145789255	5.4827521646
C	4.4400825553	2.0721378347	5.7621773115
C	5.0349375374	1.5414455895	6.8992352016
H	5.2986815760	2.1875169879	7.7301063948
C	5.3242239017	0.1779284287	6.9443740177
H	5.7799857122	-0.2439385232	7.8344019313
C	5.0635866624	-0.6296192790	5.8429858467
H	5.3149214955	-1.6853479235	5.8738977335
C	4.5142202826	-0.0981570809	4.6770269433
H	4.3802966432	-0.7254215212	3.8042777239
C	4.1897663884	1.2547754773	4.6410222550
N	3.7315380829	1.9909710466	3.5463916770
C	3.2256176812	1.5092335143	2.3410498180
C	2.5827933141	0.2964847618	2.1242998908
H	2.4596638315	-0.4151014212	2.9318745865
C	2.0522286005	0.0254835869	0.8624350263
H	1.5621279516	-0.9264697023	0.6851082721
C	2.1203220657	0.9750407618	-0.1499508295
H	1.6839286702	0.7660517475	-1.1216598916
C	2.7231501608	2.2129852287	0.0725666001
H	2.7147394182	2.9668632545	-0.7058393185
C	3.3013561695	2.4738732979	1.3128738433
N	3.8675890146	3.6454113167	1.7859090564
C	4.1338715163	4.7871370790	1.0872766023
C	4.5723085942	4.7905698515	-0.2453673016
H	4.7140280722	3.8461742712	-0.7567080655
C	4.8787154287	5.9908226133	-0.8549598691
H	5.2278734318	6.0000541098	-1.8834845715
C	4.7683052496	7.1764261623	-0.1313851763

H	5.0004145453	8.1390995235	-0.5717415799
C	4.3668576588	7.1109881084	1.1888235924
H	4.2708543291	7.9951543261	1.8102247493
N	4.0366182702	5.9512004696	1.7880384655
C	6.0275189362	3.6688919679	3.5051439010
C	6.7779742437	4.5846558860	4.2433291765
H	6.2705652666	5.2997822646	4.8871907309
C	8.1646291489	4.5865863245	4.1465918781
H	8.7442321854	5.3064461933	4.7170173943
C	8.8072063902	3.6684183543	3.3202615545
H	9.8910719162	3.6683532655	3.2477589849
C	8.0607447831	2.7507239977	2.5868489282
H	8.5598107523	2.0301391562	1.9454141805
C	6.6723949233	2.7532201266	2.6734863504
H	6.0915149822	2.0371899134	2.0970250390

b) Thermodynamic comparison of L•Pd^{PhCl} and L•Pd^{PhCl}-iso

The structural isomer of **L•Pd**^{PhCl}, **L•Pd**^{PhCl}**-iso**, was found to be 11.1 kcal/mol uphill in Gibbs free energy.

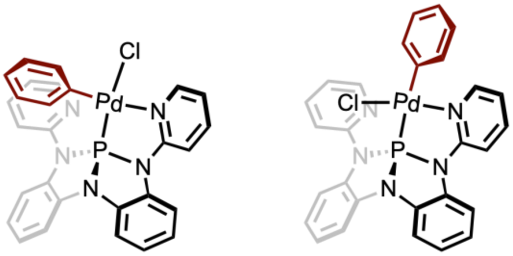

	**L · Pd**^{PhCl}	**L · Pd**^{PhCl}**-iso**
**ΔG (kcal/mol)**	0.0	11.1

VIII. References

[S1] A. Tanushi, A. T. Radosevich, "Insertion of a Nontrigonal Phosphorus Ligand into a Transition Metal Hydride: Direct Access to a Metallohydrophosphorane" *J. Am. Chem. Soc.* **2018**, *140*, 8114–8118.

[S2] W. De Graaf, J. Boersma, G. Van Koten, "Cross-coupling versus homocoupling in the reactions of dimethyl(N,N,N',N'-tetramethylethanediamine)palladium with organic halides" *Organometallics* **1990**, *9*, 1479–1484.

[S3] B. A. Markies, A. J. Canty, W. De Graaf, J. Boersma, M. D. Janssen, M. P. Hogerheide, W. J. J. Smeets, A. L. Spek, G. Van Koten, "Synthesis and structural studies of phenyl(iodo)- and methyl(phenyl)palladium(II) complexes of bidentate nitrogen donor ligands" *J. Organomet. Chem.* **1994**, *482*, 191–199.

[S4] G. Mann, D. Baranano, J. F. Hartwig, A. L. Rheingold, I. A. Guzei, "Carbon−Sulfur Bond-Forming Reductive Elimination Involving sp-, sp^2-, and sp^3-Hybridized Carbon. Mechanism, Steric Effects, and Electronic Effects on Sulfide Formation" *J. Am. Chem. Soc.* **1998**, *120*, 9205–9219.

[S5] G. R. Fulmer, A. J. M. Miller, N. H. Sherden, H. E. Gottlieb, A. Nudelman, B. M. Stoltz, J. E. Bercaw, K. I. Goldberg, "NMR Chemical Shifts of Trace Impurities: Common Laboratory Solvents, Organics, and Gases in Deuterated Solvents Relevant to the Organometallic Chemist" *Organometallics* **2010**, *29*, 2176–2179.

[S6] G. M. Sheldrick, "Crystal structure refinement with ıt SHELXL" *Acta Crystallogr., Sect. C* **2015**, *71*, 3–8.

[S7] G. M. Sheldrick, "ıt SHELXT – Integrated space-group and crystal-structure determination" *Acta Crystallogr., Sect. A* **2015**, *71*, 3–8.

[S8] P. Müller, "Practical suggestions for better crystal structures" *Crystallography Reviews* **2009**, *15*, 57–83.

[S9] S. Parsons, H. Flack, "Precise absolute-structure determination in light-atom crystals" *Acta Crystallogr., Sect. A* **2004**, *60*, s61.

[S10] P. van der Sluis, A. L. Spek, "BYPASS: an effective method for the refinement of crystal structures containing disordered solvent regions" *Acta Crystallogr., Sect. A* **1990**, *46*, 194–201.

[S11] A. L. Spek, "Structure validation in chemical crystallography" *Acta Crystallogr., Sect. D* **2009**, *65*, 148–155.

[S12] G. Sheldrick, Twinabs, *University of Göttingen, Germany* **2009**, *20*.

[S13] F. Neese, "Software update: The ORCA program system—Version 5.0" *Wiley Interdiscip. Rev.: Comput. Mol. Sci.* **2022**, *12*, e1606.

[S14] C. Adamo, V. Barone, "Toward reliable density functional methods without adjustable parameters: The PBE0 model" *J. Chem. Phys.* **1999**, *110*, 6158–6170.

[S15] F. Weigend, R. Ahlrichs, "Balanced basis sets of split valence, triple zeta valence and quadruple zeta valence quality for H to Rn: Design and assessment of accuracy" *Phys. Chem. Chem. Phys.* **2005**, *7*, 3297–3305.

[S16] E. van Lenthe, E. J. Baerends, J. G. Snijders, "Relativistic regular two-component Hamiltonians" *The J. Chem. Phys.* **1993**, *99*, 4597–4610.

[S17] E. D. Glendeni, J. K. Badenhoop, A. E. Reed, J. E. Carpenter, J. A. Bohmann, C. M. Morales, P. Karafiloglou, C. R. Landis, F. Weinhold, NBO 7.0., Theoretical Chemistry Institute, University of Wisconsin, Madison, WI (USA), **2018**.

[S18] G. Knizia, "Intrinsic Atomic Orbitals: An Unbiased Bridge between Quantum Theory and Chemical Concepts" *J. Chem. Theory Comput.* **2013**, *9*, 4834–4843.

[S19] F. Neese, "Software Update: The ORCA Program System—Version 6.0" *Wiley Interdiscip. Rev.: Comput. Mol. Sci.* **2025**, *15*, e70019.

[S20] Legault, C.Y.; CYLview, 1.0b, Université de Sherbrooke, 2009.

[S21] G. Knizia, J. E. M. N. Klein, "Electron Flow in Reaction Mechanisms—Revealed from First Principles" *Angew. Chem., Int. Ed.* **2015**, *54*, 5518–5522.

[S22] S. Grimme, A. Hansen, S. Ehlert, J.-M. Mewes, "r2SCAN-3c: A 'Swiss army knife' composite electronic-structure method" *J. Chem. Phys.* **2021**, *154*, 064103.
[S23] S. Spicher, S. Grimme, "Single-Point Hessian Calculations for Improved Vibrational Frequencies and Rigid-Rotor-Harmonic-Oscillator Thermodynamics" *J. Chem. Theory Comput.* **2021**, *17*, 1701–1714.
[S24] S. Maeda, Y. Harabuchi, Y. Ono, T. Taketsugu, K. Morokuma, "Intrinsic reaction coordinate: Calculation, bifurcation, and automated search" *Int. J. Quantum Chem.* **2015**, *115*, 258–269.
